# Mathematical modelling of stem and progenitor cell dynamics during ruxolitinib treatment of patients with myeloproliferative neoplasms

**DOI:** 10.3389/fimmu.2024.1384509

**Published:** 2024-05-07

**Authors:** Tobias Idor Boklund, Jordan Snyder, Johanne Gudmand-Hoeyer, Morten Kranker Larsen, Trine Alma Knudsen, Christina Schjellerup Eickhardt-Dalbøge, Vibe Skov, Lasse Kjær, Hans C. Hasselbalch, Morten Andersen, Johnny T. Ottesen, Thomas Stiehl

**Affiliations:** ^1^ Centre for Mathematical Modeling - Human Health and Disease, IMFUFA, Department of Science and Environment, Roskilde University, Roskilde, Denmark; ^2^ Department of Hematology, Zealand University Hospital, Roskilde, Denmark; ^3^ Institute for Computational Biomedicine and Disease Modeling, RWTH Aachen University, Aachen, Germany

**Keywords:** mathematical modelling, ordinary differential equations, myeloproliferative neoplasms (MPN), parameter estimation, JAK2 V617F, ruxolitinib, blood cancer, stem cells

## Abstract

**Introduction:**

The Philadelphia chromosome-negative myeloproliferative neoplasms are a group of slowly progressing haematological malignancies primarily characterised by an overproduction of myeloid blood cells. Patients are treated with various drugs, including the JAK1/2 inhibitor ruxolitinib. Mathematical modelling can help propose and test hypotheses of how the treatment works.

**Materials and methods:**

We present an extension of the Cancitis model, which describes the development of myeloproliferative neoplasms and their interactions with inflammation, that explicitly models progenitor cells and can account for treatment with ruxolitinib through effects on the malignant stem cell response to cytokine signalling and the death rate of malignant progenitor cells. The model has been fitted to individual patients’ data for the *JAK2* V617F variant allele frequency from the COMFORT-II and RESPONSE studies for patients who had substantial reductions (20 percentage points or 90% of the baseline value) in their *JAK2* V617F variant allele frequency (*n* = 24 in total).

**Results:**

The model fits very well to the patient data with an average root mean square error of 0.0249 (2.49%) when allowing ruxolitinib treatment to affect both malignant stem and progenitor cells. This average root mean square error is much lower than if allowing ruxolitinib treatment to affect only malignant stem or only malignant progenitor cells (average root mean square errors of 0.138 (13.8%) and 0.0874 (8.74%), respectively).

**Discussion:**

Systematic simulation studies and fitting of the model to the patient data suggest that an initial reduction of the malignant cell burden followed by a monotonic increase can be recapitulated by the model assuming that ruxolitinib affects only the death rate of malignant progenitor cells. For patients exhibiting a long-term reduction of the malignant cells, the model predicts that ruxolitinib also affects stem cell parameters, such as the malignant stem cells’ response to cytokine signalling.

## 1 Introduction

The Philadelphia chromosome-negative myeloproliferative neoplasms (MPNs) are a group of slowly progressing haematological malignancies primarily characterised by an overproduction of myeloid blood cells (1). Without treatment, they result in severe complications such as thrombosis, bleeding, infections (1), bone marrow failure, and progression to acute myelogenous leukaemia (2). The three most common MPN subtypes, essential thrombocythaemia (ET), polycythaemia vera (PV), and primary myelofibrosis (PMF), are diagnosed according to World Health Organisation (WHO) and International Consensus Classification (ICC) criteria (3), including mutational status, elevation of different cell counts (red, white, and platelets), and bone marrow morphology. A frequent common factor for the 3 subtypes of MPNs is the driver mutation *JAK2* V617F (hereinafter referred to as just *JAK2*) which is present in approximately 55% of ET patients, 98% of PV patients and 60% of PMF patients (3). Other known driver mutations in MPNs are found in the genes *CALR* and *MPL*. A subset of patients with MPN carries none of these mutations, and these patients are referred to as being triple-negative (3). In the cases where a driver mutation is present, it results in overactivation of the JAK-signal transducer and STAT-signalling (4).

The hematopoietic system is responsible for the formation of blood cells. It consists of cells of different maturity levels, starting with the least mature haematopoietic stem cells (HSCs) in the bone marrow (5), continuing with the more mature so-called progenitor and precursor cells, and ending with the fully mature cells in the peripheral blood. All haematopoietic cells are derived from the HSCs. HSC proliferation needs to fulfil two roles: maintaining the HSC pool and producing more mature committed cells that will eventually become fully mature. The hematopoietic system is subjected to a complex regulatory network which adapts the production of mature cells to the current state of the organism. It is believed that MPNs develop from a single mutated stem cell that proliferates and slowly produces both mutated stem cells, mutated progenitors, and consequently also mutated mature cells (3). If this mutated stem cell and its offspring have a proliferative advantage over the wild type cells, the mutated clone will expand and potentially cause an MPN disease. It is estimated that the time from the acquisition of the mutation to MPN diagnosis is multiple decades (3, 4). Over even longer time scales, the mutated clone may outcompete and completely eradicate the wild type cells if not treated.

The most common treatments of patients with MPN are hydroxyurea, a cytoreductive treatment that helps control the number of blood cells (2), and interferon-*α*-2a, a cytokine which is mainly depleting the bone marrow of mutated stem cells by driving them to differentiate (6). In this work, we focus on modelling the treatment with another drug: ruxolitinib (RUX), a JAK1/2 inhibitor that works by targeting the JAK1 and JAK2 kinases (4, 7) (see section 2.1.3 for more details about modelling the effects of treatment with RUX). RUX is indicated for the treatment of disease related symptoms in myelofibrosis patients and in PV patients who are resistant or intolerant to hydroxyurea, but to our knowledge its effects on the abundance of mutated cells is not yet fully understood. Studies show that RUX reduces symptom burden, spleen size, and elevated blood cell counts, thereby increasing the quality of life of the treated patients (2, 8–11), and the drug also has anti-inflammatory effects (8, 9). Mouse studies suggest that RUX primarily targets progenitors and precursor cells (12). An *in vitro* study of another JAK inhibitor, AZD1480, shows that stem cells may escape the effects of JAK inhibition (9, 13). If stem cells also escape the effects of RUX, its effects alone are insufficient to cure the disease. To cure the patients, the mutated stem cells must be eradicated (9), or, given the slow growth of the clone, reduced significantly in number. Making measurements of stem cells is neither economically nor technically practical in a routine clinical setting, and therefore it is challenging to quantify the abundance of mutated cells in the stem cell population. Clinically, the *JAK2* variant allele frequency (VAF, also called the allele burden) in the peripheral blood is used to monitor treatment response and disease progression. In patients with MPN, both heterozygous and homozygous clones are observed with ET being characterised by heterozygosity and PV by homozygosity (14). Thus, a VAF measurement of 50% could in principle mean that either 100% of cells carry a heterozygous mutation or that 50% of cells carry a homozygous mutation. In practice, a mixture of wild-type, homozygous, and heterozygous cells might be the most probable scenario. In the COMFORT-II study, the median reduction in *JAK2* VAF for 69 myelofibrosis patients during treatment with RUX was 7.0% (absolute), and 15 out of 69 had a reduction equal to or above 20% (absolute) after 48 weeks (11). In the RESPONSE study, among 104 *JAK2*-positive PV patients treated with RUX, a gradual response was seen in the mean *JAK2* VAF, and after 208 weeks the mean reduction was 40% (relative) (15).

Mechanistic mathematical modelling is a versatile tool to gain insight into complex biological processes based on limited data. Although stem cells are difficult to quantify, we can make inference about processes on the stem cell level using a mathematical model and measurements from peripheral blood. Mathematical modelling has for a long time been an important part of the study of cancers, haematopoiesis, and haematopoietic malignancies and has been employed to investigate questions such as stem cell and mature cell dynamics (16–20) and their role during disease and therapy (21), mutation acquisition and development (22, 23), clonal selection and architecture (24, 25), the role of inflammation in haematological malignancies (26–28), model-based prognostication (29, 30), therapy modelling (31, 32), and optimisation of therapy (33, 34). In this work, we extend a previous model of MPN disease dynamics and the role of inflammation in MPN, the Cancitis model (26, 28). Specifically, we extend the model by including the effects of RUX therapy in the model and by adding a progenitor compartment in the hopes of more accurately accounting for the effects of RUX on different cell types. The original Cancitis model has been successfully applied to data from patients with MPN and can capture key features of MPN progression and treatment with interferon-*α*-2a (31). Here we extend this work to model data from patients responding well to treatment with RUX.

The main objective of this work is to understand which effects of RUX treatment can explain sustained reductions in the *JAK2* VAF. In particular, we are interested in investigating whether such sustained patient responses are possible if RUX does not affect the stem cells at all, or if an effect on the stem cell level is the most straightforward explanation for the reduction observed in some patients. Determining whether or not RUX can affect and potentially eradicate the mutated stem cells is necessary to determine whether or not RUX monotherapy can potentially be a cure for MPN diseases and for predicting the patient response in case of treatment discontinuation. If the treatment does not affect the mutated stem cells, the effects of RUX are most probably palliative, and we expect that a patient discontinuing the treatment would show a disease progression. Mathematical modelling can help identify the impact of RUX on different cell types and predict patient responses to changes in the treatment schedule. A clear picture of the RUX mode of action is also important for understanding RUX’s role in combination therapy. Currently, studies of combination therapy with interferon-*α*-2a and RUX show promising results (35). To fully understand the effects of such a combination treatment with possible synergies between the drugs, a natural starting point is understanding each drug’s effects separately.

## 2 Materials and methods

The model is implemented in MATLAB version R2023b. A script simulating the model with and without treatment can be accessed on GitHub^1^.

### 2.1 Mechanistic mathematical model of MPN disease progression and RUX treatment

#### 2.1.1 Mechanistic mathematical model of MPN disease progression

**Figure 1:**
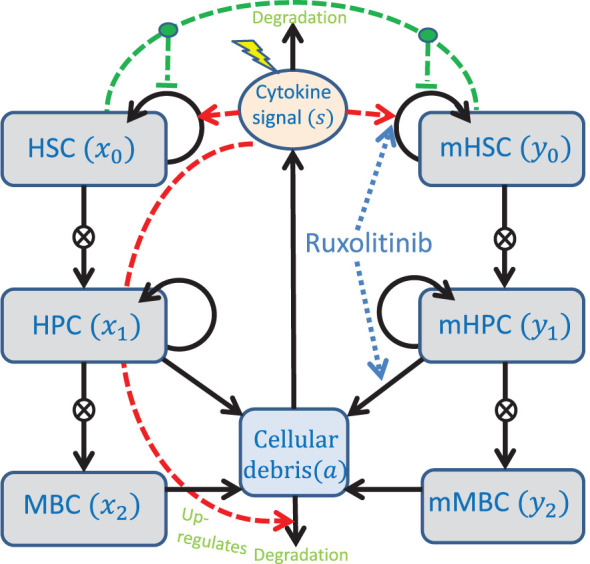
Conceptual compartment diagram of the model. See the text for further description of the model. The lightning symbol represents external factors affecting the cytokines. HSC, Haematopoietic stem cells; HPC, Haematopoietic progenitor cells; MBC, Mature blood cells; mHSC, Malignant haematopoietic stem cells; mHPC, Malignant haematopoietic progenitor cells; mMBC, Malignant mature blood cells.

We first describe the model of MPN cell dynamics in absence of treatment. The new model is a compartmental differential equation model with compartments for stem, progenitor, and mature blood cells for both healthy wild type cells and malignant cells carrying the *JAK2* mutation. In addition, there is a compartment of cellular debris from dead cells and a compartment of the cytokine signalling affecting the stem cells in the bone marrow. The compartments and their relations are depicted in **Figure 1**, an overview of the variables used is given in **Table 1**, and the equations used in the model are given in Equations (1) and (2).

**Table 1:** Overview of the variables used in the model.

Variable	Description	Expected maximal order of magnitude	Source
*x*_0_	Number of haematopoietic stem cells (HSCs)	1.0 × 10^5^	(36, 37)
*x*_1_	Number of haematopoietic progenitor cells (HPCs)	2.5 × 10^6^	(36)
*x*_2_	Number of mature blood cells (MBCs)	6.4 × 10^11^	(36)
*y*_0_	Number of malignant haematopoietic stem cells (mHSCs)	1.7 × 10^5^	Chosen
*y*_1_	Number of malignant haematopoietic progenitor cells (mHPCs)	7.6 × 10^6^	Chosen
*y*_2_	Number of malignant mature blood cells (mMBCs)	2.7 × 10^12^	Chosen
*a*	Cellular debris	1.7 × 10^3^	Chosen and (28)
*s*	Cytokine signal	2.0	Chosen

See Equations (1) and (2) for the corresponding differential equation for each variable.

All variables are considered to have unit 1, i.e. we provide total cell counts.

In the model, stem cells are capable of self-renewing as well as differentiating into progenitor cells, and progenitor cells are again capable of self-renewing [although to a lesser degree than stem cells (38)] and differentiating into mature cells. The fraction of cell divisions resulting in daughter cells adopting the same fate as the parent cell is referred to as the self-renewal fraction (38, 39). Since in reality there are many stages of cell differentiation that we lump together into “progenitors”, we include amplification factors from each maturity stage to the next. This is a well-established approach which has already been used in (16). Mature and progenitor cells can die. We assume that the death rate of stem cells is small enough as to be negligible, and therefore we exclude stem cell death from the model. We assume that the *JAK2* mutation can affect the rates of these processes, but not their kind. In other words, the wild type and malignant cell lineages are governed by the same key mechanisms, i.e. self-renewal, differentiation and death, and thus they obey equations of the same form but possibly with different parameter values.

Regulation of the haematopoietic system in our model occurs via a crowding effect on the stem cells (described in the model by the functions *ϕ_x_* and *ϕ_y_*) and feedback through cytokine signalling (described in the model by the variable *s*). The crowding effect, inspired by other modelling works (19, 20, 30, 40), models the competition for space in the stem cell niche in the bone marrow. If stem cells cannot reside in the stem cell niche, they lose stemness due to death or differentiation. In this model, this effect is described by the monotonically decreasing functions *ϕ_x_* and *ϕ_y_* in Equation (2) which are identical to the ones in (28). The cytokine feedback is modelled by saturating functions, in this case Michaelis-Menten expressions, that up-regulate the self-renewal fraction of stem cells through the bone marrow microenvironment. The cytokine level is up-regulated by the amount of cellular debris, as well as by an external inflammatory load (representing, e.g., smoking or other illnesses). Debris from dead cells accumulate and is cleared at a rate proportional to the cytokine level. It is important to note that the cytokine level, *s*, is a lumped parameter that represents different feedbacks in the body, including the immune system’s response to cell death (both at equilibrium and as a response to externally imposed cell death) and inflammation.

Using these assumptions, the differential equations describing the system are given by


(1a) $$\underset{\text{rate of change of HSCs}}{\underset{︸}{{\dot{x}}_{0}}}=\underset{\text{proliferation rate}}{\underset{︸}{\alpha_{x_{0}}}}(\underset{\text{self-renewal}}{\underset{︸}{\underset{\overset{︷}{p_{x_{0}}}}{\max.\text{self-renewal fraction}}\;\underset{\overset{︷}{\phi_{x}(x_{0},\;y_{0})}}{\text{crowding}}\;\underset{\overset{︷}{\frac{s}{s_{x_{0}}+s}}}{\text{cytokine signal}}}}-\underset{\text{differentiation}}{\underset{︸}{\left(1-p_{x_{0}}\phi_{x}(x_{0},\;y_{0})\frac{s}{s_{x_{0}}+s}\right)}})x_{0,}$$

(1b) $$\begin{array}{l} \underset{\text{rate of change of HPCs}}{\underset{︸}{{\dot{x}}_{1}}}=\underset{\text{proliferation rate}}{\underset{︸}{\alpha_{x_{1}}}}\left(\underset{\text{self-renewal}}{\underset{︸}{p_{x_{1}}}}-\underset{\text{differentiation}}{\underset{︸}{\left(1-p_{x_{1}}\right)}}\right)x_{1}+\underset{\text{amplification}}{\underset{︸}{A_{x_{0}}}}\underset{\text{influx from HSCs}}{\underset{︸}{2\alpha_{x_{0}}\left(1-p_{x_{0}}\phi_{x}(x_{0},\;y_{0})\frac{s}{s_{x_{0}}+s}\right)x_{0}}}-\underset{\text{death}}{\underset{︸}{d_{x_{1}}x_{1}}}\\ \; \end{array},$$

(1c) $$\underset{\text{rate of change of MBCs}}{\underset{︸}{{\dot{x}}_{2}}}=\underset{\text{amplification}}{\underset{︸}{A_{x_{1}}}}\underset{\text{influx from HPCs}}{\underset{︸}{2\alpha_{x_{1}}(1-p_{x_{1}})x_{1}}}-\underset{\text{death}}{\underset{︸}{d_{x_{2}}x_{2}}},$$

(1d) $$\underset{\text{rate of change of mHSCs}}{\underset{︸}{{\dot{y}}_{0}}}=\underset{\text{proliferation rate}}{\underset{︸}{\alpha_{y_{0}}}}\left(\underset{\text{self-renewal}}{\underset{︸}{\overset{\max.\text{ self-renewal fraction}}{\overset{︷}{p_{y_{0}}}}\overset{\text{crowding}}{\overset{︷}{\phi_{y}(x_{0,\;}y_{0})}}\overset{\mathrm{cytokine signal}}{\overset{︷}{\frac{s}{s_{y_{0}}+s}}}}}-\underset{\mathrm{differentiation}}{\underset{︸}{\left(1-p_{y_{0}}\phi_{y}(x_{0,\;}y_{0})\frac{s}{s_{y_{0}}+s}\right)}}\right)y_{0,}$$

(1e) $$\begin{array}{l} \underset{\text{rate of change of mHPCs}}{\underset{︸}{{\dot{y}}_{1}}}=\underset{\text{proliferation rate}}{\underset{︸}{\alpha_{y_{1}}}}\left(\underset{\text{self-renewal}}{\underset{︸}{p_{y_{1}}}}-\underset{\text{differentiation}}{\underset{︸}{\left(1-p_{y_{1}}\right)}}\right)y_{1}+\underset{\text{amplification}}{\underset{︸}{A_{y_{0}}}}\underset{\text{influx from mHSCs}}{\underset{︸}{2\alpha_{y_{0}}\left(1-p_{y_{0}}\phi_{y}(x_{0},\;y_{0})\frac{s}{s_{y_{0}}+s}\right)y_{0}}}-\underset{\text{death}}{\underset{︸}{d_{y_{1}}y_{1}}},\\ \; \end{array}$$

(1f) $$\underset{\text{rate of change of mMBCs}}{\underset{︸}{{\dot{y}}_{2}}}=\underset{\text{amplification}}{\underset{︸}{A_{y_{1}}}}\underset{\text{influx from mHPCs}}{\underset{︸}{2\alpha_{y_{1}}(1-p_{y_{1}})y_{1}}}-\underset{\text{death}}{\underset{︸}{d_{y_{2}}y_{2}}},$$

(1g) $$\underset{\text{rate of change of debris}}{\underset{︸}{\dot{a}}}=\underset{\text{dead cells}}{\underset{︸}{d_{x_{1}}x_{1}+d_{y_{1}}y_{1}+d_{x_{2}}x_{2}+d_{y_{2}}y_{2}}}-\underset{\text{degradation}}{\underset{︸}{e_{a}as}},$$

(1h) $$\underset{\text{rate of change of cytokine signal}}{\underset{︸}{\dot{s}}}=\underset{\text{production}}{\underset{︸}{r_{s}a}}-\underset{\text{degradation}}{\underset{︸}{e_{s}s}}+\underset{\text{external factors}}{\underset{︸}{I}},$$

where *ϕ_x_* and *ϕ_y_* are given by


(2a) $$\underset{\text{crowding function for HSCs}}{\underset{︸}{\phi_{x}(x_{0},\;y_{0})}}=\frac{1}{1+\underset{\text{inhibition by HSCs}}{\underset{︸}{c_{xx}x_{0}}}+\underset{\text{inhibition by mHSCs}}{\underset{︸}{c_{xy}y_{0}}}},$$

(2b) $$\underset{\text{crowding function for mHSCs}}{\underset{︸}{\phi_{y}(x_{0},\;y_{0})}}=\frac{1}{1+\underset{\text{inhibition by HSCs}}{\underset{︸}{c_{yx}x_{0}}}+\underset{\text{inhibition by mHSCs}}{\underset{︸}{c_{yy}y_{0}}}},$$

An explanation of the sources, estimations, and choices of the default parameter values used in the model is given in **section S1** of the supplementary. There, we also show a simple sensitivity analysis of the system. This shows that the model is most sensitive to changes in $p_{x_{0}}$ and $p_{y_{0}}$ followed by $c_{xx}$ , $c_{yx}$ , $\alpha_{y_{0}}$ , and $s_{x_{0}}$ . These parameters are the most sensitive because they, except $\alpha_{y_{0}}$ , determine the self-renewal fraction of the healthy and the malignant stem cells, the products $p_{x_{0}}\phi_{x}(x_{0},\;y_{0})\frac{s}{s_{x_{0}}+s}$ and, $p_{y0}\phi_{y}\left(x_{0},\;y_{0}\right)\frac{s}{s_{y_{0}}+s}$ , respectively, and that the self-renewal fraction is the main contributor to the overall fitness of each of the cell lines (25). Analogous results have been reported for other models (20, 24, 29, 30). An overview of the parameter values used in this work can be found in **Table 2**. The model is designed to be generally applicable to patients with MPN, but due to biological variation the parameter values might vary from patient to patient. In this work, the parameter values used give a typical course of the disease. For future reference, we refer to $s_{x_{0}}$ and $s_{y_{0}}$ as the half-saturation constants of the healthy and malignant, respectively, stem cell response to cytokine signalling.

**Table 2:** Parameter values for the model in Equations (1) and (2).

Parameter	Description	Value	Unit	Source
$\alpha_{x_{0}}$	Proliferation rate of HSCs	3.6 × 10^−3^	day^−1^	(37, 41)
$\alpha_{y_{0}}$	Proliferation rate of mHSCs	5.4 × 10^−3^	day^−1^	Estimated
$p_{x_{0}}$	Self-renewal fraction for HSCs	0.89	1	Estimated
$p_{y_{0}}$	Self-renewal fraction for mHSCs	0.97	1	Chosen
*c_xx_*	Constant for HSCs inhibiting HSC self-renewal	5.6 × 10^−6^	1	Estimated
*c_yx_*	Constant for HSCs inhibiting mHSC self-renewal	5.2 × 10^−6^	1	Estimated
*c_xy_*	Constant for mHSCs inhibiting HSC self-renewal	5.4 × 10^−6^	1	Estimated
*c_yy_*	Constant for mHSCs inhibiting mHSC self-renewal	5.0 × 10^−6^	1	Estimated
$s_{x_{0}}$	Half-saturation constant for cytokine signal for HSCs	1.4 × 10^−1^	1	Chosen
$s_{y_{0}}$	Half-saturation constant for cytokine signal for mHSCs	7.1 × 10^−2^	1	Chosen
$A_{x_{0}}$	Amplification factor from HSCs to HPCs	3.4 × 10^1^	1	Estimated
$A_{y_{0}}$	Amplification factor from mHSCs to mHPCs	3.4 × 10^1^	1	Estimated
$\alpha_{x_{1}}$	Proliferation rate of HPCs	1.1 × 10^−2^	day^−1^	Chosen
$\alpha_{y_{1}}$	Proliferation rate of mHPCs	1.7 × 10^−2^	day^−1^	Chosen
$p_{x_{1}}$	Self-renewal fraction for HPCs	0.445	1	Chosen
$p_{y_{1}}$	Self-renewal fraction for mHPCs	0.485	1	Chosen
$d_{x_{1}}$	Death rate of HPCs	3.7 × 10^−3^	day^−1^	Chosen
$d_{y_{1}}$	Death rate of mHPCs	3.7 × 10^−3^	day^−1^	Chosen
$A_{x_{1}}$	Amplification factor from HPCs to MBCs	3.2 × 10^6^	1	Estimated
$A_{y_{1}}$	Amplification factor from mHPCs to mMBCs	3.2 × 10^6^	1	Estimated
$d_{x_{2}}$	Death rate of MBCs	1.5 × 10^−1^	day^−1^	(36)
$d_{y_{2}}$	Death rate of mMBCs	1.5 × 10^−1^	day^−1^	(36)
*e_a_*	Degradation rate for *a*	1.2 × 10^8^	day^−1^	Estimated
*r_s_*	Production rate for *s*	8.6 × 10^−2^	day^−1^	(28)
*e_s_*	Degradation rate for *s*	7.2 × 10^1^	day^−1^	Estimated
*I*	External up-regulation of *s*	2	day^−1^	(28)

A unit of 1 means that the given parameter is dimensionless.

#### 2.1.2 Steady states of the model

Next, we present the steady states of the model to illustrate the range of behaviours that can be captured by it. The steady states of the system in Equations (1) and (2) arise as solutions of complicated rational equations which we solve numerically. We define a biologically feasible steady state as a solution to the steady state problem in which all variables are real and non-negative. For the standard parameter values given in **Table 2**, there exist 12 possible steady states of which 5 are biologically feasible. The local stabilities of these steady states are calculated numerically using the eigenvalues of the corresponding Jacobian matrices, see **Table 3**. We denote a steady state without any cells as “trivial”, a steady state with only healthy cells as “healthy”, and a steady state with only malignant cells as “malignant”.

**Table 3:** Biologically feasible steady states for the model in Equations (1) and (2) with the standard choice of parameters in **Table 2**.

*x*_0_	*x*_1_	*x*_2_	*y*_0_	*y*_1_	*y*_2_	*a*	*s*	Type	Stability
0	0	0	0	0	0	0	2.8×10^−2^	Trivial	Locally stable
3.2×10^3^	8.0×10^4^	2.1×10^10^	0	0	0	1.4×10^2^	1.9×10^−1^	Healthy	Locally unstable
9.9×10^4^	2.5×10^6^	6.3×10^11^	0	0	0	8.1×10^2^	9.9×10^−1^	Healthy	Locally unstable
0	0	0	1.6×10^2^	6.8×10^3^	2.4×10^9^	4.0×10^1^	7.6×10^−2^	Malignant	Locally unstable
0	0	0	1.7×10^5^	7.6×10^6^	2.7×10^12^	1.7×10^3^	2.0	Malignant	Locally stable

From **Table 3**, we see that for the standard parameter values in **Table 2**, there exists a locally stable trivial steady state, two locally unstable healthy steady states, and both a locally stable and a locally unstable malignant steady state. Thus, two locally stable steady states exist: a trivial one and a malignant one. However, if one considers the case with 0 malignant cells, i.e. $y_{0}=y_{1}=y_{2}$ = 0 and then disregards the equations for these variables, only the trivial and the healthy steady states remain, and in this case the healthy steady state with 9.9 × 10^4^ stem cells, for which the model was calibrated (see **section S1** of the supplementary for more details), becomes locally stable. It may seem a bit counter-intuitive that the trivial steady state is locally stable both in the case with and without malignant cells present. However, numerical experiments show that for the case of only healthy cells being present, $x_{0}$ , $x_{1}$ , and $x_{2}$ should all be below 3.24% of their locally stable healthy steady state values for the system to approach the trivial steady state, and for the case of only malignant cells being present, $y_{0}$ , $y_{1}$ , and $y_{2}$ should all be below 0.10% of their locally stable malignant steady state values for the system to approach the trivial steady state. If this is not the case, the system approaches the locally stable healthy steady state and the locally stable malignant steady state, respectively. Thus, in conclusion, with the standard choice of parameters in **Table 2**, the system approaches the locally stable malignant steady state unless extremely few cells are present. In the case of only healthy cells being present, the system instead approaches the (in that case) locally stable healthy steady state.

#### 2.1.3 Modelling patient responses to treatment with ruxolitinib

Now, we discuss how the effects of RUX can be accounted for in the model. RUX is a non-specific JAK1/2 inhibitor that targets the JAK1 and JAK2 kinases (7), and it has multiple effects on patients with MPN. In the following, we investigate potential effects of RUX on mutated cells. As a readout for therapy effects, we use the *JAK2* VAF. Studies have shown that RUX treatment reduces blood cell counts both in mice (12) and in humans (2, 8, 11). In mice, RUX is unable to target the mutated disease-initiating stem cells, but it depletes erythroid progenitors and precursors (12). As mentioned in the introduction, an *in vitro* study of another JAK inhibitor, AZD1480, shows that stem cells may escape the effects of JAK inhibition (13). Additionally, RUX gives mild reductions in the *JAK2* VAF in mice and minimal to moderate reductions in humans with high variability between patients (2, 9), and the reductions are sustained on therapy (2, 9, 11, 15).

Systematic numerical analysis of the model specified in Equations (1) and (2) reveals that a sustained reduction in *JAK2* VAF can only be achieved if treatment with RUX affects the mHSC dynamics described by Equation (1d) (see **section S3** of the supplementary for more details). Biologically, this can be interpreted as a direct effect on the mHSCs or an effect on the mHSC response to the cytokine signal for these cells. Here, we choose to interpret one effect of RUX as a reduction of the cytokine-induced up-regulation of mHSC self-renewal. This is achieved by letting RUX increase $s_{y_{0}}$ . To model the reduction of cell counts and the targeting of progenitor cells, we also let RUX affect the death rate of malignant progenitor cells, i.e. we let it increase $d_{y_{1}}$ . The numerical experiments with the model also reveal that this effect alone can give rapid reductions in the blood cell counts, and it can also reduce the *JAK2* VAF in the short term. In the long term, however, the *JAK2* VAF typically increases again when only this parameter is increased.

Let $\tilde{s_{y_{0}}}$ and $\tilde{d_{y_{1}}}$ denote the values of $s_{y_{0}}$ and $d_{y_{{\;}_{1}}}$ , respectively, for a given patient during treatment with RUX, let $\rho_{s_{y_{0}}}$ and $\rho_{d_{y_{1}}}$ denote patient specific parameters describing the strength of a given patient’s response to RUX treatment in terms of $s_{y_{0}}$ and $d_{y_{{\;}_{1}}}$ , and let $c_{R}(t)$ denote the dose of RUX that the given patient is receiving measured in mg/day. Then, we assume that the effects of RUX treatment are dose-dependent in the following way:


(3a) $$\tilde{s_{y_{0}}}(t)=(1+c_{R}(t)\rho_{s_{y_{0}}})s_{y_{0}},$$

(3b) $$\tilde{d_{y_{1}}}(t)=(1+c_{R}(t)\rho_{d_{y_{1}}})d_{y_{1}}.$$

In this work, we consider only the case of $\rho_{s_{y_{0}}}\geq \;0$ and $\rho_{d_{y_{1}}}\geq \;0$ , i.e. that RUX can increase the values of $s_{y_{0}}$ and $d_{y_{{\;}_{1}}}$ . It is worth pointing out that only relative changes in $c_{R}(t)$ matter, as a scaling of $c_{R}(t)$ can be compensated for by using the inverse scaling for $\rho_{s_{y_{0}}}$ and $\rho_{d_{y_{1}}}$ . Using these updated parameter values due to RUX treatment and collecting some terms from Equation (1) for brevity, the model takes the following form during treatment:


(4a) $${\dot{x}}_{0}=\alpha_{x_{0}}\left(2p_{x_{0}}\phi_{x}(x_{0},\;y_{0})\frac{s}{s_{x_{0}}+s}-1\right)x_{0},$$

(4b) $${\dot{x}}_{1}=\alpha_{x_{1}}(2p_{x_{1}}-1)x_{1}+2A_{x_{0}}\alpha_{x_{0}}\left(1-p_{x_{0}}\phi_{x}(x_{0},\;y_{0})\frac{s}{s_{x_{0}}+s}\right)x_{0}-d_{x_{1}}x_{1},$$

(4c) $${\dot{x}}_{2}=2A_{x_{1}}\alpha_{x_{1}}(1-p_{x_{1}})x_{1}-d_{x_{2}}x_{2},$$

(4d) $${\dot{y}}_{0}=\alpha_{y_{0}}\left(2p_{y_{0}}\phi_{y}(y_{0},\;y_{0})\frac{s}{(1+c_{R}(t)\rho_{s_{y_{0}}})s_{y_{0}}+s}-1\right)y_{0},$$

(4e) $${\dot{y}}_{1}=\alpha_{y_{1}}(2p_{y_{1}}-1)y_{1}+2A_{y_{0}}\alpha_{y_{0}}\left(1-p_{y_{0}}\phi_{y}(x_{0},\;y_{0})\frac{s}{(1+c_{R}(t)\rho_{s_{y_{0}}})s_{y_{0}}+s}\right)y_{0}-(1+c_{R}(t)\rho_{d_{y_{1}}})d_{y_{1}}y_{1},$$

(4f) $${\dot{y}}_{2}=2A_{y_{1}}\alpha_{y_{1}}(1-p_{y_{1}})y_{1}-d_{y_{2}}y_{2},$$

(4g) $$\dot{a}=d_{x_{1}}x_{1}+d_{y_{1}}y_{1}+d_{x_{2}}x_{2}+d_{y_{2}}y_{2}-e_{a}as,$$

(4h) $$\dot{s}=r_{s}a-e_{s}s+I,$$

where the *ϕ*-functions are once again given in Equation (2), and the assumed effects of RUX are highlighted in blue and cyan.

### 2.2 Data

The largest part of the data used in this work is taken from the COMFORT-II study (11). The COMFORT-II study was an open-label phase 3 randomised controlled study that investigated the safety and efficacy of ruxolitinib vs. best available therapy (BAT) in 219 patients with myelofibrosis (MF). The primary end point of the study was the percentage of patients with at least a 35% reduction in spleen volume after 48 weeks, but an exploratory response assessment included monitoring the *JAK2* VAF (42). More information about the study can be found in (11, 42)^2^. In the supplementary of (11), trajectories of the evolution of the *JAK2* VAF are presented for 18 individual patients who achieved a reduction in *JAK2* VAF of at least 20% (absolute) after 48 or 72 weeks of RUX treatment. This is approximately 16.5% of the patients from the study who were treated with RUX and were carrying the *JAK2* V617F mutation. We include these 18 patients in our study.

Additional data were obtained from the RESPONSE study (15). The RESPONSE study was an open-label phase 3 randomised controlled study that investigated the safety and efficacy of ruxolitinib vs. BAT in 222 patients with polycythaemia vera (PV). The primary end point of the study was haematocrit control through week 32 and at least a 35% reduction in spleen volume after 32 weeks (43). The study also monitored the *JAK2* VAF (15, 43)^3^. In (15), trajectories of the evolution of the *JAK2* VAF are presented for a number of patients who crossed over from interferon-*α*-2a to RUX and for patients who achieved a 90% (relative) reduction in *JAK2* VAF. From the latter category, 6 patients received only RUX, and we include data from these 6 patients in the data used in this work. We assign the numbers 19 through 24 to the patients from the RESPONSE study. These 6 patients correspond to approximately 6% of the patients from the study who were treated with RUX and were carrying the *JAK2* V617F mutation.

It is important to note that we do not have access to the full data sets from the COMFORT-II and RESPONSE studies but only to the data shown in the respective publications, which is precisely the data for patients achieving substantial (defined as above for the respective studies) reductions in their *JAK2* VAF. This is a limited subset of the patients in the respective studies, and the rest of the patients in the studies have not responded as well to the treatment. However, if the model developed here can fit to the patients experiencing the largest reductions in *JAK2* VAF, it seems reasonable to assume that the model may also fit to patients achieving a more modest response in their *JAK2* VAF as this requires less drastic changes to the parameters of the model as a result of the treatment. While it would be optimal to have data for all levels of response to the treatment, we can still learn about the most important mechanisms of RUX by considering patients responding well to the treatment.

We do not have access to changes to the dosing of RUX for the individual patients. In the COMFORT-II study, the median daily dose was 40 mg/day for patients with platelet counts above 200 × 10^9^ L^−1^ and 30 mg/day for patients with platelet counts between 100×10^9^ L^−1^ and 200×10^9^ L^−1^ (11). Both median daily doses were slightly decreasing over time during the study. Here, we compromise and assume that the dose for the available COMFORT-II patients was constant at 35 mg/day, i.e. $c_{R}(t)$ = 35 for these patients. In the RESPONSE study, the initial dose was 20 mg/day (15), and therefore we assume that $c_{R}(t)$ = 20 for these patients. We once again emphasise that the absolute value of $c_{R}(t)$ is irrelevant for each patient, and only relative changes matter. The absolute value is only used to compare the resulting values of $\tilde{s_{y_{{\;}_{0}}}}\left(t\right)$ and $\tilde{d_{y_{1}}}\left(t\right)$ between patients. Since we assume a constant daily dose of RUX for all patients, the time-dependence of $\tilde{s_{y_{{\;}_{0}}}}\left(t\right)$ and $\tilde{d_{y_{1}}}\left(t\right)$ vanishes, and hereinafter we do not write it explicitly.

All patient data used in this work were extracted from plots in the publications mentioned above using WebPlotDigitizer^4^.

### 2.3 Fitting the model to clinical data

We fit the model to the *JAK2* VAF of the patients using the patient-specific parameters $\rho_{s_{y_{0}}}$ and $\rho_{d_{y_{1}}}$ . For each patient, we compute the values of $\rho_{s_{y_{0}}}$ and $\rho_{d_{y_{1}}}$ that give model predictions the closest to their *JAK2* VAF data in a nonlinear least squares framework as described in **section S4** of the supplementary. In all calculations, the *JAK2* VAF is used as a decimal number, but it is plotted as a percentage as this is what is most commonly done in the clinic. The quality of the fits is quantified using the root mean squared error (RMSE). For data points ${\left\{y_{i}\right\}}_{i=1}^{m}$ and model predictions $\{{\hat{y}}_{i}{\left(t_{i};\;\rho_{s_{y_{0}}},\;\rho_{d_{y_{1}}}\right\}}_{i=1}^{m}$ , the RMSE is given by


$$RMSE=\sqrt{\frac{1}{m}\sum_{i=1}^{m}\left(y_{i}-\hat{y}(t_{i};\;\rho_{s_{y_{0}}},\;\rho_{d_{y_{1}}}\right){)}^{2}}.$$

The RMSE is easier to interpret than the sum of squared errors since, due to the square root, it has the same unit as the data themselves, and it gives a measure of the typical (but not the mean) error between the model and the data. For example, an RMSE-value of 0.05 (5%) means that the typical difference between the *JAK2* VAF data and the model predictions is 0.05 (5%).

We use the *JAK2* VAF as a proxy for the fraction of mutated (malignant) cells, and we make the simplifying assumption that all mutated cells are homozygous. This assumption is motivated by the observation that the average *JAK2* VAF of the 24 patients used in this work was approximately 76% at the initiation of the respective studies (see section 2.2 for further description of the data used). If we assume that all cells in a given patient are mutated and let *a* denote the fraction of mutated cells that are homozygous, the *JAK2* VAF is given by $V=\frac{1}{2}(1-a)+a=\frac{1}{2}(1+a)$ . From this expression, we can calculate that in the “worst” case where all cells are mutated, if the *JAK2* VAF is 0.76 (76%), at least the fraction 0.52 (52%) of the cells must be homozygous. If not all cells are mutated, an even higher percentage of the cells must be homozygous. Therefore, we will use the fraction


$$g(x_{2},\;y_{2})=\frac{y_{2}}{x_{2}+y_{2}},$$

with the output from the model in Equations (1) or (4) as our best estimate of the *JAK2* VAF and thus compare this quantity to the available measurements.

## 3 Results

### 3.1 Model simulations suggest that RUX must affect both stem cells and progenitor cells to achieve sustained reductions in the *JAK2* VAF

As described in section 2, our model consists of 8 ordinary differential equations [see Equations (1) and (2)] describing the time evolution of the number of healthy and malignant stem cells, healthy and malignant progenitor cells, healthy and malignant mature cells, the cellular debris, and a cytokine signal. In this model, we have interpreted the effects of RUX as affecting the half-saturation constant of the malignant stem cell response to the cytokine signal, $s_{y_{0}}$ , and the death rate of malignant progenitor cells, $d_{y_{{\;}_{1}}}$ [see Equations (1) and (3)]. As motivated in section 2.1.3, the effect on $s_{y_{0}}$ is needed for the model to achieve sustained reductions in the *JAK2* VAF on therapy (2, 9, 11, 15), and the effect on $d_{y_{{\;}_{1}}}$ models the reduction in blood cell counts (2, 8, 11) through targeting of the mutated progenitor cells (12). To show how these effects synergise, we simulate the population dynamics of healthy and malignant cells for a typical *in silico* patient with different adjustments to the default values of $s_{y_{0}}$ and $d_{y_{{\;}_{1}}}$ . Specifically, we are investigating how to achieve the (relatively) quick and monotonic reduction in *JAK2* VAF that some patients experience.

**Figure 2:**
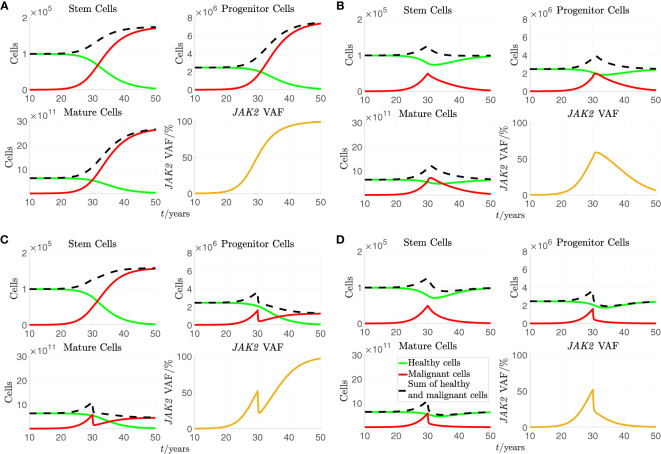
A simulation of the stem, progenitor and mature cell counts and the *JAK2* VAF based on Equations (1) and (2) with the standard parameters from **Table 2**. As initial conditions, we choose $x_{0}(0)=1.0\times 10^{5}$ , $x_{1}(0)=2.5\times 10^{6}$ , $x_{2}(0)=6.4\times 10^{11}$ , $y_{0}(0)=1$ , $y_{1}(0)=0$ , $y_{2}(0)=0$ , $a(0)=8.1\times 10^{2}$ , and s(0)=1 . For the plots of cell counts, the green curves represent the number of healthy cells, the solid red curves represent the number of malignant cells, and the dashed black curves represent the sum of healthy and malignant cells. Treatment is initiated after 30 years in the simulation. **(A)** No effect of treatment. **(B)**. $s_{y_{0}}$ increased to 6 times its standard value during treatment. **(C)** $d_{y_{1}}$ to 6 times its standard value during treatment. **(D)** $s_{y_{0}}$ and $d_{y_{1}}$ to 6 times their standard values during treatment.

We initialise the simulations with the initial conditions $x_{0}(0)=1.0\times 10^{5}$ , $x_{1}(0)=2.5\times 10^{6}$ , $x_{2}(0)=6.4\times 10^{11}$ , $y_{0}(0)=1$ , $y_{1}(0)=0$ , $y_{2}(0)=0$ , $a(0)=8.1\times 10^{2}$ , and s(0)=1 . These initial conditions approximately correspond to the second healthy steady state in **Table 3**, for which the model was calibrated (see **section S1** of the supplementary) with one malignant stem cell added. After 30 years, the *JAK2* VAF has reached approximately 50%, and we initiate treatment with RUX. Since the effects of RUX on cell kinetics are not well understood, we consider four scenarios of how the treatment may affect the parameters of the model: a) RUX has no effect on the patient. b) RUX affects only the half-saturation constant for the malignant stem cells, $s_{y_{0}}$ . c) RUX affects only the death rate of malignant progenitor cells, $d_{y_{{\;}_{1}}}$ d) RUX affects both $s_{y_{0}}$ and $d_{y_{{\;}_{1}}}$ . These scenarios are based on the hypothesised mechanisms of RUX interpreted in terms of the model (see section 2.1.3). Scenario a) illustrates the scenario of a patient not responding to the treatment. This could for example be a patient who is resistant to RUX. It also illustrates the behaviour of the model in absence of treatment. Scenarios b) and c) illustrate the model behaviour when RUX causes only one of the two hypothesised treatment effects from section 2.1.3. This illustrates the individual effect of each of the two hypothesised treatment effects in the model and could illustrate the scenarios of patients in whom the treatment affects only one of the two parameters. Finally, scenario d) illustrates the model behaviour when RUX causes both of the hypothesised treatment effects. The results of the simulations of all four scenarios are shown in **Figure 2**.

In all subfigures of **Figure 2**, we see that the number of malignant cells and the *JAK2* VAF rise from close to 0 and until treatment initiation at time 30 years. From **Figure 2A**, we see that if a) RUX has no effect on the patient, the number of malignant cells continues to increase before saturating while all the healthy cells are outcompeted, and the *JAK2* VAF increases to 100%. From **Figure 2B**, we see that if b) the treatment with RUX affects only the half-saturation constant of the mHSCs, $s_{y_{0}}$ , the mHSCs are outcompeted, and the patient is cured, but only after a considerable period of several decades. However, more mHPC and mMBC are produced initially due to increased differentiation of the mHSCs, and both the number of mHPC, the number of mMBC, and the *JAK2* VAF grow during approximately the first year of treatment before declining. In this simulation, it takes approximately 2 years before the number of mMBCs returns to its level just before treatment initiation and approximately 4 years before the *JAK2* VAF returns to its level just before treatment initiation. Thus, this type of effect might actually be harmful to the patient in the first couple of years. Furthermore, this temporary increase of the *JAK2* VAF is not observed, and thus, this effect alone cannot explain the available data. However, without affecting the stem cells directly, i.e. their proliferation rate, their maximal self-renewal fraction, their interactions with each other (the crowding effects), or introducing a death rate for them (see **section S3** of supplementary for plots showing some of these effects), adjusting the half-saturation constant of the mHSC, $s_{y_{0}}$ , is the only possibility for observing a sustained reduction in the cell counts and the *JAK2* VAF. From **Figure 2C**, we see that if c) the treatment with RUX changes only the death rate of the mHPCs, $d_{y_{{\;}_{1}}}$ , the number of mHPCs, the number of mMBCs, and the *JAK2* VAF will decrease for approximately half a year and adjust to a new quasi-steady state, but since the mHSCs are completely unaffected, the number of these continues to grow. After the initial decline due to the increased death rate of mHPCs, the number of mHPCs and the number of mMBCs grow slowly with the mHSCs, and eventually all the healthy cells are outcompeted. Thus, affecting $d_{y_{{\;}_{1}}}$ alone is not curative and cannot explain the monotonically decreasing *JAK2* VAF observed in some patients. Summing up, letting RUX affect only $s_{y_{0}}$ or $d_{y_{{\;}_{1}}}$ alone is not sufficient to explain the quick and monotonic reduction in *JAK2* VAF that some patients experience during treatment. However, in **Figure 2D** we see that if d) the treatment with RUX affects both $s_{y_{0}}$ and $d_{y_{{\;}_{1}}}$ , and the respective parameters are sufficiently increased compared to the scenario without treatment, both the number of mHSCs, mHPCs, mMBCs, and the *JAK2* VAF may all monotonically decrease during treatment, and thus the patient will experience remission in both the long and the short run. Thus, changing both $s_{y_{0}}$ and $d_{y_{{\;}_{1}}}$ simultaneously is one mechanism in the model that can explain the effect of RUX treatment.

### 3.2 The proposed model can recapitulate the response dynamics during RUX therapy

**Figure 3:**
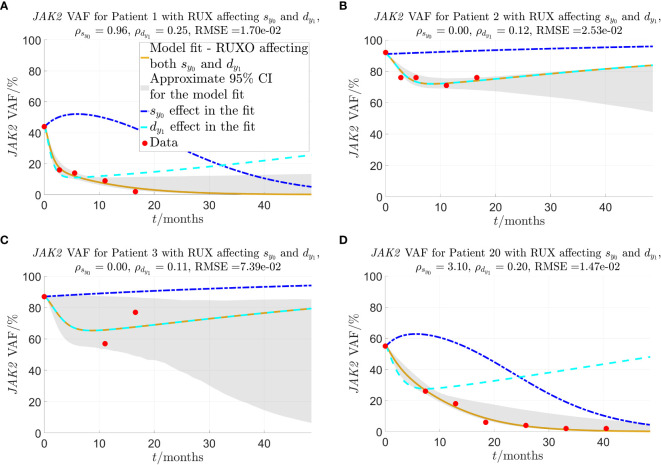
Model fit to selected individual patients as described in sections 2.2-2.3. $\rho_{s_{y_{0}}}$ and $\rho_{d_{y_{1}}}$ are the fitted parameters that quantify the strength of the given patient’s response to RUX treatment in terms of the effect on $s_{y_{0}}$ and $d_{y_{{\;}_{1}}}$ , respectively. The solid yellow curves show the optimal fit of the model to the *JAK2* VAF data. In the fit, it is assumed that both parameters $\rho_{s_{y_{0}}}$ (response of mHSCs to cytokine signal) and $\rho_{d_{y_{1}}}$ (malignant progenitor cell death) are affected by RUX at the same time. To visualise the impact of each of the two effects (changed response to cytokines and increased progenitor death) on the *JAK2* VAF dynamics, the dashed lines show the time evolution of *JAK2* VAF if either $\rho_{d_{y_{1}}}$ (blue) or $\rho_{s_{y_{0}}}$ (cyan) is set to 0 and the respective other parameter remains unchanged. The red dots are the data. **(A)** Patient 1, one of the patients for whom the model fits very well, and for whom the model predicts that RUX affects both $s_{y_{0}}$ and $d_{y_{{\;}_{1}}}$ . **(B)** Patient 2, a patient for whom the model fits quite well, and the model predicts that RUX affects only $d_{y_{{\;}_{1}}}$ ( $\rho_{s_{{\;}_{y}{\;}_{0}}}=0$ ). **(C)** Patient 3, the patient for whom the model fits worst. **(D)** Patient 20, a patient from the RESPONSE study for whom the model fits very well, and for whom the model predicts that RUX affects both $s_{y_{0}}$ and $d_{y_{1}}$ .

**Figure 4:**
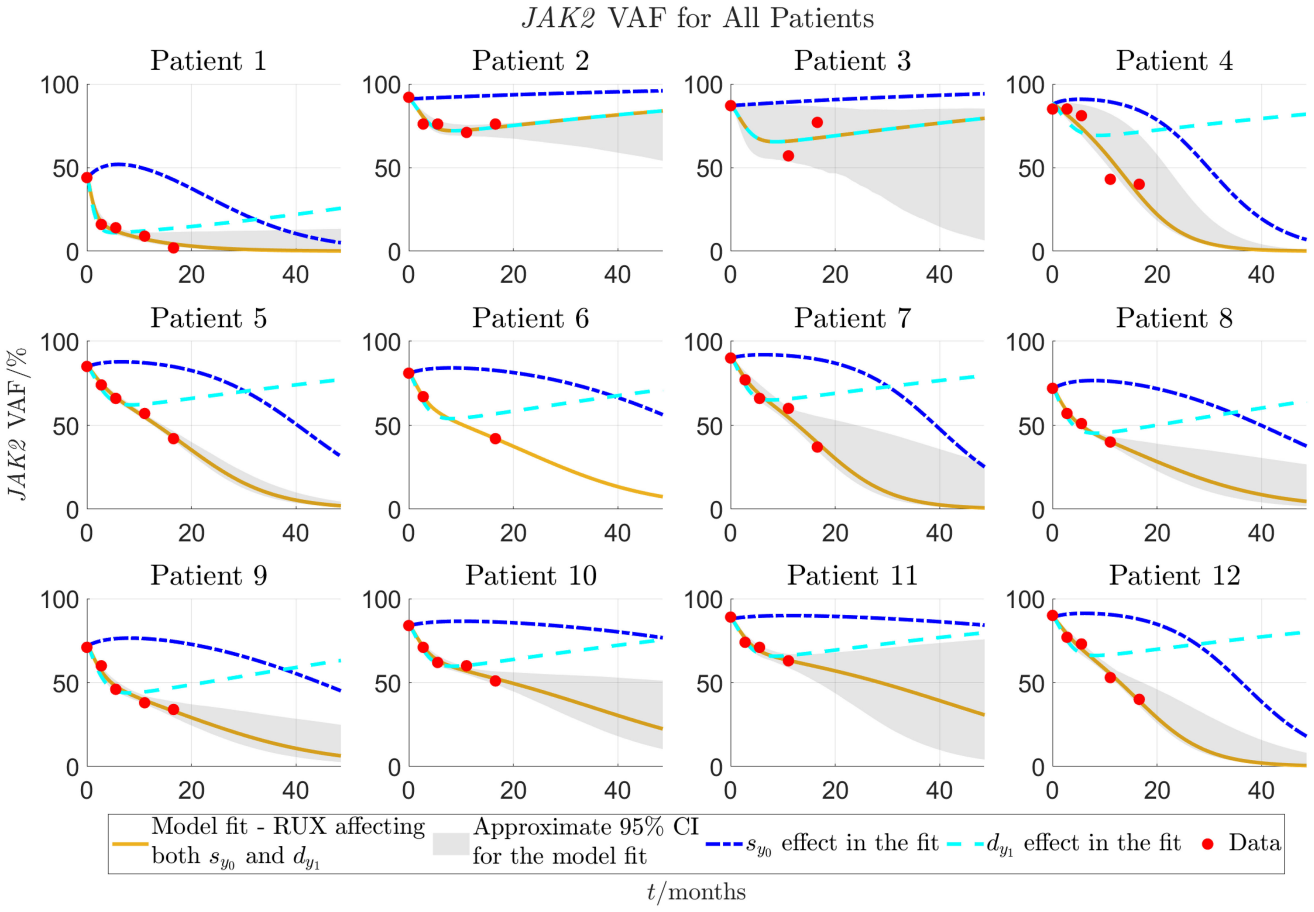
Model fit to individual patients’ data for patients 1-12 as described in sections 2.2-2.3. The solid yellow curves show the optimal fits of the model to the *JAK2* VAF data. In the fit it is assumed that both parameters $\rho_{s_{y_{0}}}$ (response of mHSCs to cytokine signal) and $\rho_{d_{y_{1}}}$ (malignant progenitor cell death) are affected by RUX at the same time. To visualise the impact of each of the two effects (changed response to cytokines and increased progenitor death) on the VAF dynamics, the dashed lines show the time evolution of JAK2 VAF if either $\rho_{d_{y_{1}}}$ (blue) or $\rho_{s_{y_{0}}}$ (cyan) is set to 0 and the respective other parameter remains unchanged. The red dots are the data. Patients 1-12 are from the COMFORT-II study.

**Figure 5:**
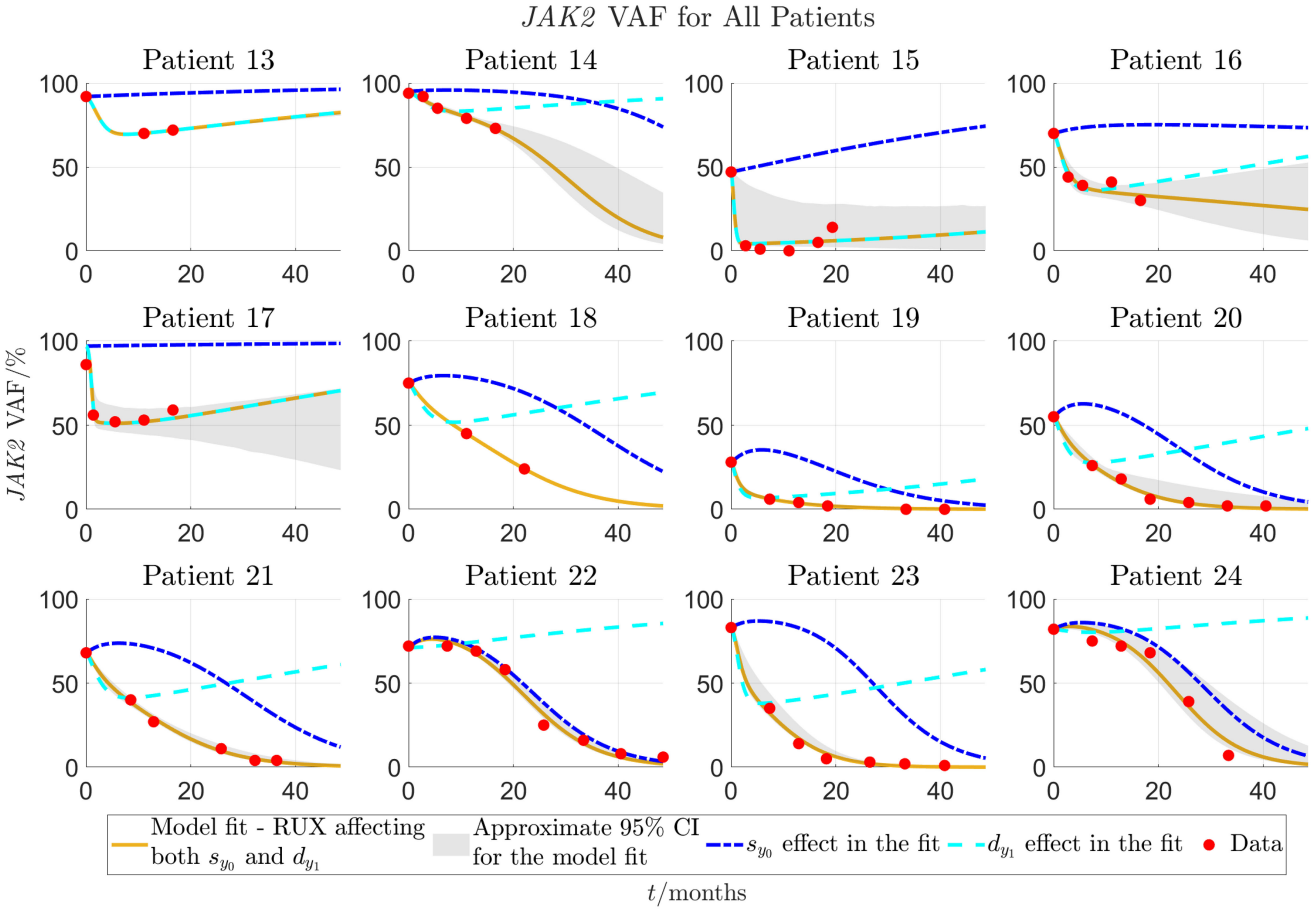
Continuation of **Figure 4** for patients 13-24. Patients 13-18 are from the COMFORT-II study, and patients 19-24 are from the RESPONSE study.

To further investigate and quantify the effects of RUX treatment on the half-saturation constant for the mHSCs’ response to the cytokine signal, $s_{y_{0}}$ , and the death rate of the mHPCs, $d_{y_{{\;}_{1}}}$ , we fit the model in Equation (4) to individual patients’ data. More precisely, we adapt the parameters $\rho_{s_{y_{0}}}$ and $\rho_{d_{y_{1}}}$ describing the size of each patient’s change in $s_{y_{0}}$ and $d_{y_{{\;}_{1}}}$ , respectively, due to the treatment with RUX to obtain the optimal fits. The results for the individual patients can be seen in detail in **section S6** of the supplementary, some representative examples of fits are shown in **Figure 3**, and all fits are presented in **Figures 4**, **5**. In these figures, time t=0 is defined as the time of the first available *JAK2* VAF measurement. Using the fits, we are able to quantify how much the affected parameters change for each patient (see **Table 4**) and to make predictions of the time dynamics of the *JAK2* VAF for each patient if the treatment is continued (see **section S6** of the supplementary). We also compare the model fits with RUX affecting both $s_{y_{0}}$ and $d_{y_{{\;}_{1}}}$ (**Figures 3**–**5**) to the cases of RUX affecting only $s_{y_{0}}$ or $d_{y_{{\;}_{1}}}$ (effectively setting $\rho_{d_{y_{1}}}=0$ and $\rho_{s_{y_{0}}}=0$ , respectively, see the figures in **section S7** of the supplementary). It should be noted that the reported approximate 95% confidence intervals (CIs) are calculated by sampling 1000 pairs of the parameters from their estimated approximate joint distribution, simulating the model with the sampled parameters, and finally taking the middle 95% predicted *JAK2* VAF values of these simulations. The sampling procedure can produce negative values of the parameters, in which case we choose to resample the corresponding samples. More details are given in **section S4** of the supplementary.

**Table 4:** Overview of the fitted parameters, $\rho_{s_{y_{0}}}$ and $\rho_{d_{y_{1}}}$ , and the parameter values $\tilde{s_{y_{0}}}$ and $\tilde{d_{y_{1}}}$ from Equation (3) during RUX treatment for all patients.

Patient	Dose/ mg/day	$\rho_{s_{y_{0}}}$ / (mg/day)^−1^ (approx. 95% CI)	$\tilde{s_{y_{0}}}$ /1	$\frac{\tilde{s_{y_{0}}}}{s_{y_{0}}}$	$\rho_{d_{y_{1}}}$ / (mg/day)^−1^ (approx. 95% CI)	$\tilde{d_{y_{1}}}$ / day^−1^	$\frac{\tilde{d_{y_{1}}}}{d_{y_{1}}}$
1	35	0.959 (-1.55, 3.46)	2.47	34.6	0.248 (0.166, 0.329)	0.0358	9.66
2	35	0 (-0.226, 0.226)	0.0714	1	0.122 (0.0805, 0.164)	0.0195	5.27
3	35	0 (-6.47, 6.47)	0.0714	1	0.11 (-1.37, 1.59)	0.018	4.86
4	35	9.13e+04 (-1.45e+10, 1.45e+10)	2.28e+05	3.19e+06	0.0994 (-0.0156, 0.214)	0.0166	4.48
5	35	0.861 (0.342, 1.38)	2.22	31.1	0.108 (0.0914, 0.125)	0.0177	4.79
6	35	0.397 (0.394, 0.399)	1.06	14.9	0.118 (0.118, 0.118)	0.019	5.12
7	35	1.43 (-1.04, 3.91)	3.66	51.2	0.155 (0.0971, 0.213)	0.0238	6.43
8	35	0.443 (-0.196, 1.08)	1.18	16.5	0.102 (0.0845, 0.119)	0.0169	4.57
9	35	0.367 (0.0253, 0.708)	0.988	13.8	0.109 (0.0859, 0.133)	0.0178	4.82
10	35	0.245 (-0.000628, 0.492)	0.685	9.59	0.112 (0.0897, 0.134)	0.0182	4.92
11	35	0.221 (-0.632, 1.07)	0.623	8.72	0.12 (0.0812, 0.159)	0.0193	5.2
12	35	1.93 (-0.735, 4.59)	4.89	68.4	0.131 (0.0956, 0.166)	0.0206	5.58
13	35	0 (-0.0839, 0.0839)	0.0714	1	0.161 (0.131, 0.192)	0.0246	6.64
14	35	0.635 (0.0722, 1.2)	1.66	23.2	0.116 (0.087, 0.144)	0.0187	5.05
15	35	0 (-0.527, 0.527)	0.0714	1	0.813 (-0.632, 2.26)	0.109	29.5
16	35	0.125 (-0.199, 0.449)	0.383	5.37	0.143 (0.0923, 0.194)	0.0223	6.01
17	35	0 (-0.173, 0.173)	0.0714	1	1.05 (0.57, 1.54)	0.14	37.9
18	35	0.765 (0.763, 0.767)	1.16	16.3	0.0882 (0.0881, 0.0883)	0.0102	2.76
19	20	1.74 (0.361, 3.12)	2.55	35.8	0.379 (0.305, 0.452)	0.0317	8.58
20	20	3.1 (-0.874, 7.07)	4.5	63	0.198 (0.148, 0.249)	0.0184	4.96
21	20	1.78 (0.784, 2.78)	2.62	36.7	0.176 (0.142, 0.21)	0.0167	4.52
22	20	4.28e+05 (-1.29e+11, 1.29e+11)	6.11e+05	8.55e+06	0.00826 (-0.0264, 0.043)	0.00431	1.17
23	20	7.38e+06 (-2.6e+13, 2.6e+13)	1.05e+07	1.48e+08	0.483 (0.274, 0.693)	0.0395	10.7
24	20	11.4 (-96.5, 119)	16.3	229	0.0268 (-0.0374, 0.0911)	0.00568	1.54

Note that patients from the COMFORT-II trial (numbered 1-18) received a different daily dose of RUX than patients from the RESPONSE study (numbered 19-24).

The fractions $\frac{\tilde{s_{y_{0}}}}{s_{y_{0}}}$ and $\frac{\tilde{d_{y_{1}}}}{d_{y_{1}}}$ are the ratios between the respective parameters during and before treatment. For the cases where the lower limit of the approximate 95% CIs of the fitting parameters $\rho_{s_{y_{0}}}$ and $\rho_{d_{y_{1}}}$ is less than 0, this should be interpreted as a lower limit of 0 as the optimal fit is calculated under the conditions $\rho_{s_{y_{0}}}$ ≥ 0 and $\rho_{d_{y_{1}}}$ ≥ 0.

**Figure 3A** shows that for patient 1, the model fits very well to the *JAK2* VAF with an RMSE-value of 0.0170 (1.70%) for the *JAK2* VAF data and approximate 95% CIs of mean width 0.0978 (9.78%) for the time shown in the plot. **Figure 3B** shows another example of a good fit for patient 2 with an RMSE-value of 0.0253 (2.53%) for the *JAK2* VAF data and approximate 95% CIs of mean width 0.141 (14.1%) for the time shown in the plot. Compared to patient 1, the model predicts that for this patient, RUX treatment affects only $d_{y_{{\;}_{1}}}$ (since $\rho_{s_{y_{0}}}=0$ ), and therefore the reduction in *JAK2* VAF is temporary, and the patient is not cured in the long run. In fact, this turns out to be the case for 5 out of the 24 patients, namely patients 2, 3, 13, 15, and 17. This shows that our model is able to classify patients in terms of their response to RUX, which has the potential to be of key clinical significance. For these 5 patients, their *JAK2* VAF is initially decreasing and then increasing at later time points. The fits for these patients are shown in **Figures 4**, **5** and in more detail in **section S6** of the supplementary. **Figure 3C** shows the worst fit of the model to the available data. This happens for patient 3 with an RMSE-value of 0.0739 (7.39%) for the *JAK2* VAF data and approximate 95% CIs of mean width 0.483 (48.3%) for the time shown in the plot. For this patient, we are therefore very uncertain about the future development of the *JAK2* VAF. **Figure 3D** shows another example of a good fit to the data for a patient from the RESPONSE study with an RMSE-value of 0.0147 (1.47%) for the *JAK2* VAF data and approximate 95% CIs of mean width 0.0853 (8.53%) for the time shown in the plot. This shows that the model and the data fitting are robust with respect to the medical studies and the diagnoses of the patients (myelofibrosis in COMFORT-II, PV in RESPONSE).

Plots showing the convergence of the fitting procedure to the final fits using all data are shown in **section S6** of the supplementary. Boxplots of the RMSE-values for all data points as function of the number of data points used in the fit is shown in **Supplementary Figure S15**. Overall, the quality of the fits improves significantly when more data points are added. The mean RMSE of the fits is 0.25 (25%) when only 2 data points are used in the calculation of the optimal fit, and 0.09 (9%), 0.05 (5%), and 0.03 (3%) when 3, 4, and 5 data points are used, respectively.

**Figure 6:**
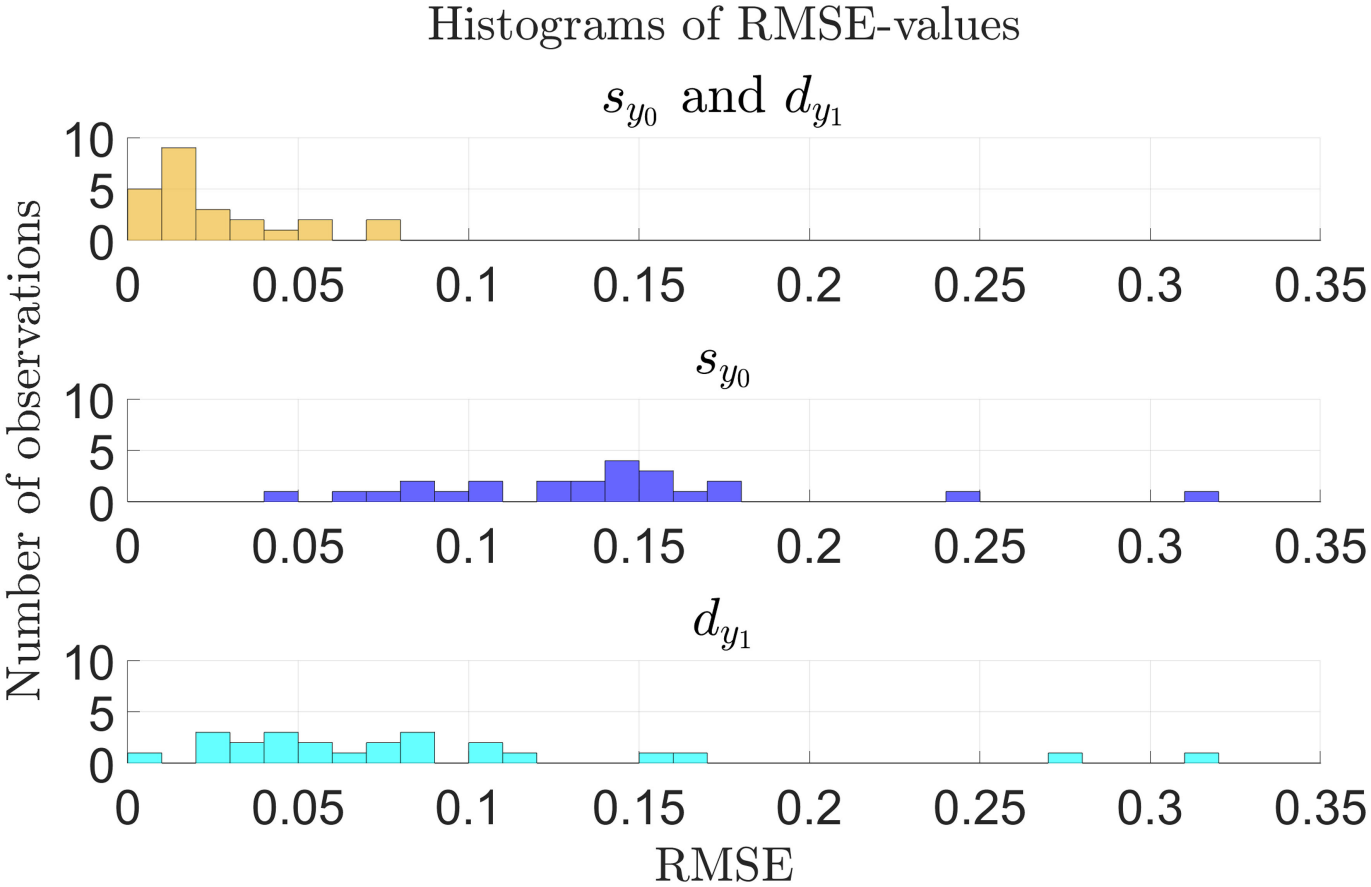
Histogram of RMSE-values for the model fitted to the individual patient *JAK2* VAF data as described in sections 2.2-2.3 for all patients (*n* = 24) as presented in **Figures 4**, **5** and in **sections S6** and **S7** of the supplementary. Yellow: Optimal fits allowing RUX treatment to affect both $s_{y_{0}}$ and $d_{y_{{\;}_{1}}}$ . Blue: Optimal fits allowing only changes in $s_{y_{0}}$ . Cyan: Optimal fits allowing only changes in $d_{y_{{\;}_{1}}}$ . There are no observations with RMSE-values outside the range shown in the plots.

**Figure 6** shows histograms of the RMSE-values from fitting the model to all 24 patients and allowing RUX treatment to affect the half-saturation constant for the mHSCs’ response to the cytokine signal, $s_{y_{0}}$ , and the death rate of the mHPCs, $d_{y_{{\;}_{1}}}$ , at the same time and either effect separately. Here, we see that the RMSE-values are much smaller when allowing RUX treatment to affect both $s_{y_{0}}$ and $d_{y_{{\;}_{1}}}$ than when allowing it to affect only one of them. We obtain a mean RMSE-value of 0.0249 (2.49%) when both $s_{y_{0}}$ and $d_{y_{{\;}_{1}}}$ can be affected at the same timed compared to 0.138 (13.8%) and 0.0874 (8.74%) when only $s_{y_{0}}$ or $d_{y_{{\;}_{1}}}$ can be affected, respectively. Additionally, when RUX is allowed to affect both $s_{y_{0}}$ and $d_{y_{{\;}_{1}}}$ , the model fits achieve an RMSE-value equal to or below 0.02 (2%) for 14 out of 24 patients (approximately 58.3% of patients) and equal to or below 0.04 (4%) for 19 out of 24 patients (approximately 79.2% of patients). The corresponding numbers are 0 (0%) and 0 (0%) when allowing RUX treatment to affect only $s_{y_{0}}$ , and 1 (approximately 4.17% of patients) and 6 (25.0% of patients) when allowing RUX treatment to affect only $d_{y_{{\;}_{1}}}$ . Thus, the model fits much better to the available data in the scenario where RUX treatment affects both $s_{y_{0}}$ and $d_{y_{{\;}_{1}}}$ compared to the scenarios where it affects only one of the parameters, supporting the hypothesis that RUX treatment affects parameters in both the equations for the number of malignant stem cells and the number of malignant progenitor cells. Plots of the optimal model fits to the data for the individual patients when allowing RUX treatment to affect only $s_{y_{0}}$ or $d_{y_{{\;}_{1}}}$ are shown in **section S7** of the supplementary.

Finally, in **Table 4** we compare the fitted half-saturation constant for the mHSCs’ response to the cytokine signal, $s_{y_{0}}$ , and the fitted death rate of the mHPCs, $d_{y_{{\;}_{1}}}$ , before and during RUX treatment. A graphical illustration with histograms of $\tilde{s_{y_{0}}}$ and $\tilde{d_{y_{{\;}_{1}}}}$ is shown in **Supplementary Figure S17**. We consider the scenario where both parameters can change in the presence of RUX. On average during treatment, $s_{y_{0}}$ is increased to 21.7 times its pre-treatment value (an increase from 7.1 × 10^−2^ to 1.6) with a standard deviation of 20.9 times, and $d_{y_{1}}$ is increased to 5.35 times its pre-treatment value (an increase from 3.7 × 10^−3^ day^−1^ to 2.0 × 10^−2^ day^−1^) with a standard deviation of 2.21 times. Thus, the treatment seems to have a substantial effect on the cell parameters of the responding patients. In the summary statistics for the changes in $s_{y_{0}}$ just mentioned, we have disregarded patients 4, 22, 23, and 24 who are considered outliers due to them having $s_{y_{0}}$ increased to 3.19 × 10^6^, 8.55 × 10^6^, 1.48 × 10^8^, and 229 times their pre-treatment values, respectively. Similarly, in the summary statistics for $d_{y_{1}}$ just mentioned, we have disregarded patients 15 and 17 who are considered to be outliers due to them having $d_{y_{1}}$ increased to 29.5 and 37.9 times their pre-treatment values, respectively. See **Table 4** for the full details. For the cases where the lower limit of the approximate 95% CIs of the fitted parameters $\rho_{s_{y_{0}}}$ and $\rho_{d_{y_{1}}}$ is less than 0, this should be interpreted as a lower limit of 0 as the optimal fit is calculated under the conditions $\rho_{s_{y_{0}}}$ ≥ 0 and $\rho_{d_{y_{1}}}$ ≥ 0.

## 4 Discussion

In this work, we have proposed a mechanistic model of RUX treatment in MPN patients. The model is able to capture quantitative *JAK2* VAF dynamics in patients showing significant VAF reductions in response to RUX. In the model, RUX affects the malignant HSCs’ response to the cytokine signal and the malignant progenitor cell death rate. The former is quantified by the half-saturation constant, $s_{y_{0}}$ , and the latter is denoted by $d_{y_{1}}$ . The mean RMSE-value of the fits is 0.0249 (2.49%) when allowing RUX treatment to affect both $s_{y_{0}}$ and $d_{y_{1}}$ . The model suggests that a RUX-dependent increase of malignant progenitor cell death and a RUX-dependent down-regulation of the response of malignant HSCs to the feedback signal are sufficient to reproduce clinical data. The results should be interpreted as model-generated hypotheses which require further experimental validation.

To achieve lasting reductions in *JAK2* VAF in the model simulations, as is seen for at least some patients, any kind of treatment must affect parameters which are linked to the stem cell population dynamics, i.e. the stem cell proliferation rates and/or their self-renewal fraction. If a treatment does not affect these quantities, the model predicts that the treatment will only cause temporary reductions in *JAK2* VAF before it starts increasing again. This is in contrast to some sources stating that RUX is not able to target the disease-initiating malignant stem cells in mice (12) and in humans (9). As 18 of the 24 patients considered in this work have not had an increase in *JAK2* VAF from one measurement to another, our model predicts that RUX could affect the mHSCs by inhibiting their response to the cytokine signalling in the bone marrow, i.e. by increasing the half-saturation constant for the mHSCs’ response to the cytokine signal, $s_{y_{0}}$ . However, as seen from the fitting to individual patients’ *JAK2* VAF data, the best fits for the 5 patients numbered 2, 3, 13, 15, and 17 are obtained by the RUX treatment not affecting $s_{y_{0}}$ but instead affecting only the death rate of the malignant progenitor cells, $d_{y_{1}}$ . Thus, it is possible that RUX treatment does not affect the stem cell parameters in some patients, but that it does so in others. The 5 patients in this data set for whom the best fits are obtained by having RUX not affecting stem cell parameters are precisely the ones experiencing an initial reduction in *JAK2* VAF followed by a monotonic increase at the later time points. The final patient experiencing an increase in *JAK2* VAF from one measurement to another is patient 16. For this patient, the *JAK2* VAF increases from measurement 3 to measurement 4, but then decreases again from measurement 4 to measurement 5. Therefore, the model predicts that RUX treatment also affects $s_{y_{0}}$ for this patient. Thus, our hypothesis from this data fitting is that if a patient experiences an initial reduction in *JAK2* VAF followed by monotonic growth, RUX affects only progenitor cell parameters for this patient. If this is not the case, most typically due to monotonic reductions in the *JAK2* VAF in this data set, the model predicts that RUX affects some stem cell parameter for the given patient, for example $s_{y_{0}}$ . This hypothesis can theoretically be tested by making measurements of cell lines and in animal models. The model predicts that the sustained reductions in *JAK2* VAF are due to a reduction in the number of malignant stem cells. Thus, the model predicts that for the patients experiencing sustained reductions in *JAK2* VAF, continued treatment with RUX may ultimately result in a complete eradication of malignant cells.

It is important to point out that the model here is fitted to data from patients who achieved a reduction in *JAK2* VAF of at least 20% (absolute) after 48 or 72 weeks of treatment in the COMFORT-II study and at least 90% (relative) in the RESPONSE study, and these constitute only a subset of the cohorts (approximately 16.5% and 6% of *JAK2* positive patients treated with RUX, respectively). Since we do not have access to the data of the rest of the patients in these studies, it is impossible to fit the model to their data. However, since these patients have experienced only modest reductions in their *JAK2* VAF (or maybe even increases), it seems reasonable to assume that the model could possibly fit to these patients without changing $s_{y_{0}}$ in response to RUX treatment. Thus, it is possible that for the majority of patients, RUX treatment does not affect the stem cell parameters, but for a minority of patients it does so in addition to affecting the progenitor parameters. In the latter case we observe monotonically decreasing *JAK2* VAF dynamics.

For patients 4, 22, 23, and 24, the model predicts that $s_{y_{0}}$ should be increased to 3.19 × 10^6^, 8.55 × 10^6^, 1.48 × 10^8^, and 229 times its pre-treatment value, respectively, to obtain the optimal fits. This seems excessive, but due to the Michaelis-Menten functional form in which $s_{y_{0}}$ appears, $\frac{s}{s_{y_{0}}+s}$ , these increases all effectively reduce the self-renewal fraction of the mHSCs, $p_{y_{0}}\phi_{y}(y_{0},\;y_{0})\frac{s}{s_{y_{0}}+s}$ , to 0. If $s_{y_{0}}$ is sufficiently high, the self-renewal fraction becomes insensitive to changes of this parameter.

Processes not considered in the model can lead to disagreements between data and simulations. Some major potential sources of model error are the following:

- *Biological variation between patients:* To avoid overfitting and to keep the model as simple and interpretable as possible, we have fitted the model to the data by letting RUX affect only two parameters and letting all other parameters be equal for all patients. In reality, RUX may affect more than the two parameters investigated. Furthermore, the remaining parameters most probably differ between patients and may even vary over time for each specific individual, e.g. due to differences in age, sex, BMI, etc. However, changing some parameters, for example $e_{a}$ , $r_{s}$ , $e_{s}$ , and I , results in only minor effects on the cell counts and the *JAK2* VAF (see **sections S2** (sensitivity analysis) and **S3** (numerical experiments) of the supplementary for more details). Therefore, we believe that we have captured the most important effects of RUX in this model.
- *Assuming constant daily doses of RUX:* In the model, we assume that the patients have received constant daily doses of RUX. However, in reality, each patient has most likely received a varying daily dose of RUX dependent on their response to the drug, including side effects, their doctors’ recommendations, etc. A varying dosing of RUX will most likely have an impact on the fitted parameter values.
- *Comorbidities and other conditions:* The patients might have been affected by comorbidities and other conditions during the studies. For example, since it is believed that inflammation affects the development of MPNs (44, 45), inflammatory processes might impact on the treatment response.
- *Modelling precisely one malignant clone:* MPNs are known to be one of the cancers with the lowest number of mutations (14), making these diseases well suited for this type of model with only one malignant clone. However, some patients may have multiple competing malignant clones. To account for different mutations, the model has to be extended accordingly.
- *Resistance to RUX:* The model only implicitly accounts for potential resistance to RUX. One study has shown that 16 out of 39 MF patients were considered to be resistant to RUX, of which 4 were considered to be primary resistant (46). In other studies, the percentage of patients being primary resistant to RUX was estimated to be 2-5% (47). In the COMFORT-II study, approximately 15% of patients discontinued treatment with RUX due to disease progression (11), signifying either primary or secondary resistance. Resistance to RUX will be reflected by low values of $\rho_{s_{y_{0}}}$ and $\rho_{d_{y_{1}}}$ in the optimal fit. However, this neglects that resistance can develop over time. Letting $\rho_{s_{y_{0}}}$ and $\rho_{d_{y_{1}}}$ be time-dependent would result in a much more complicated model and in a higher risk for overfitting.

Another source of error are the measurement errors, the size of which is unknown to us, but which depends on the equipment and techniques used in the laboratory. In both the COMFORT-II and the RESPONSE studies, the *JAK2* VAF was measured using qPCR methods (11, 15). Though we do not know the exact size of the measurement errors, one study of qPCR methods has shown that for one particular set of equipment and techniques, the standard deviations of the measurements were 0.012 (1.2%) in a reference sample with a *JAK2* VAF of 0.045 (4.5%) and 0.035 (3.5%) in a reference sample with a *JAK2* of 0.13 (13%) (48). These standard deviations are close to the mean RMSE of the model fitted to the available data (0.0249), and thus the deviations between the model and the data are of a reasonable order of magnitude. In the data fitting, we have assumed that the overall errors, i.e. the sum of the model errors and the measurements errors, are normally distributed with 0 mean and some variance, *σ* ^2^. This convenient assumption makes the statistical analysis of the results simple (compared to the alternatives, see **section S4** of the supplementary for more details), but it is hard to either verify or refute this assumption based on 3-8 data points per patient. The previously mentioned study of different qPCR methods suggests that the size of the measurement errors might depend on the true value of the of the *JAK2* VAF (48). However, simple experiments using weighted least squares fitting gave almost identical results for all patients except patient 15, and therefore we have chosen to use the simpler ordinary least squares approach here. Furthermore, least square fitting approaches can be susceptible to outliers. However, by inspecting the data visually, we have no reason to believe that any one point is an obvious outlier.

In the results presented, we have chosen to fit the data from each patient individually, and thus every patient is completely independent of the other patients. This assumption reduces the computational costs of the fitting procedure. Fitting parameters using the framework of mixed effect models is theoretically possible, however, it increases the computational complexity.

Besides merely testing whether or not specific hypotheses about RUX effects are compatible with clinical data, the model provides a quantitative estimate of the size of a given patient’s response to RUX treatment, uncertainty quantification on these estimates, and predictions of the future development of the *JAK2* VAF. The patient-specific parameters, $\rho_{s_{y_{0}}}$ and $\rho_{d_{y_{1}}}$ , can potentially be used to predict how a given patient will respond to changes in the doses of RUX. For example, it can be used to predict the future development of the *JAK2* VAF if the patient continues or discontinues a specific treatment protocol (for example due to side effects), and it can be used to calculate a critical dose that must be given to the patient to achieve eventual remission. However, such an estimate has to be carefully validated on real world data, and further refinement of the model, e.g., with respect to resistance development, might be required.

## Data availability statement

Publicly available datasets were analysed in this study. This data can be found here: In the supplementary of Cervantes et al. (2013), doi.org/10.1182/blood-2013-02-485888 (11), and in the main text of Vannucchi et al. (2017), doi.org/10.1007/s00277-017-2994-x (15).

## Ethics statement

Ethical approval was not required for the study involving humans in accordance with the local legislation and institutional requirements. Written informed consent to participate in this study was not required from the participants or the participants’ legal guardians/next of kin in accordance with the national legislation and the institutional requirements.

## Author contributions

TB: Conceptualization, Formal analysis, Investigation, Methodology, Software, Validation, Visualization, Writing – original draft, Writing – review & editing. JS: Conceptualization, Writing – review & editing. JG-H: Conceptualization, Writing – review & editing. ML: Conceptualization, Writing – review & editing. TK: Conceptualization, Writing – review & editing. CE-D: Conceptualization, Writing – review & editing. VS: Conceptualization, Writing – review & editing. LK: Conceptualization, Writing – review & editing. HH: Conceptualization, Writing – review & editing. MA: Conceptualization, Formal analysis, Investigation, Methodology, Project administration, Supervision, Validation, Visualization, Writing – review & editing. JO: Conceptualization, Formal analysis, Investigation, Methodology, Project administration, Supervision, Validation, Visualization, Writing – review & editing. TS: Conceptualization, Formal analysis, Funding acquisition, Investigation, Methodology, Project administration, Supervision, Validation, Visualization, Writing – review & editing.

## Footnotes

1
https://github.com/TobiasBoklund/Math_Modelling_of_RUX_Treatment_in_MPN.git.

2
https://clinicaltrials.gov/study/NCT00934544.

3
https://clinicaltrials.gov/study/NCT01243944.

4
https://automeris.io/WebPlotDigitizer/.
