# Supplementary material for: Mathematical modelling of stem and progenitor cell dynamics during ruxolitinib treatment of patients with myeloproliferative neoplasms
Source: Front Immunol. 2024 May 7;15:1384509. doi: 10.3389/fimmu.2024.1384509 (PMC11154009; doi:10.3389/fimmu.2024.1384509)
Supplement: Supplementary file 1 [file Presentation_1.pdf]

# Supplementary Material for Boklund et. al - Mathematical Modelling of Ruxolitinib Treatment

## 1 PARAMETERISATION

In this section, we describe the parametrization of the proposed model in the absence of treatment with RUX. To ease the reading, we first repeat the model here (collecting some terms for brevity):

$$\dot{x}_0 = \alpha_{x_0} \left( 2p_{x_0}\phi_x(x_0, y_0)\frac{s}{s_{x_0}+s} - 1 \right) x_0, \tag{S1a}$$

$$\dot{x}_1 = \alpha_{x_1} (2p_{x_1} - 1) x_1 + 2A_{x_0}\alpha_{x_0} \left( 1 - p_{x_0}\phi_x(x_0, y_0)\frac{s}{s_{x_0}+s} \right) x_0 - d_{x_1}x_1, \tag{S1b}$$

$$\dot{x}_2 = 2A_{x_1}\alpha_{x_1} (1 - p_{x_1}) x_1 - d_{x_2}x_2, \tag{S1c}$$

$$\dot{y}_0 = \alpha_{y_0} \left( 2p_{y_0}\phi_y(y_0, y_0)\frac{s}{s_{y_0}+s} - 1 \right) y_0, \tag{S1d}$$

$$\dot{y}_1 = \alpha_{y_1} (2p_{y_1} - 1) y_1 + 2A_{y_0}\alpha_{y_0} \left( 1 - p_{y_0}\phi_y(x_0, y_0)\frac{s}{s_{y_0}+s} \right) y_0 - d_{y_1}y_1, \tag{S1e}$$

$$\dot{y}_2 = 2A_{y_1}\alpha_{y_1} (1 - p_{y_1}) y_1 - d_{y_2}y_2, \tag{S1f}$$

$$\dot{a} = d_{x_1}x_1 + d_{y_1}y_1 + d_{x_2}x_2 + d_{y_2}y_2 - e_a a s, \tag{S1g}$$

$$\dot{s} = r_s a - e_s s + I, \tag{S1h}$$

where $\phi_x$ and $\phi_y$ are given by

$$\phi_x(x_0, y_0) = \frac{1}{1 + c_{xx}x_0 + c_{xy}y_0}, \tag{S2a}$$

$$\phi_y(x_0, y_0) = \frac{1}{1 + c_{yx}x_0 + c_{yy}y_0}. \tag{S2b}$$

The model is designed to recapitulate important disease dynamics. Due to the lack of high resolution personalised data we assume that a subset of parameters are identical for all individuals. The other parameters are fitted based on individual patients' data. This approach might reduce the applicability of the model to individuals which are not well represented by the chosen parameters. However, the model still captures general features of the disease and provides good fits for a large number of patients. Our approach for the parameterisation of the model is to first calibrate the model to the case of healthy haematopoiesis. Afterwards, we choose the parameters of mutated cells such that the model exhibits realistic dynamics.

- The proliferation rate of healthy stem cells is estimated to be approximately once every 40 weeks in one source (1), and the time between two self-renewal divisions of HSCs is estimated to range from 2 to 20 months (approximately 8.7-87 weeks) in another source (2). Therefore, the proliferation rate is set to $\alpha_{x_0} = \frac{1}{280}$ day$^{-1}$ $= 3.6 \times 10^{-3}$ day$^{-1}$.

- We set the value of $s_{x_0}$ to be $\frac{1}{7}$. This choice is arbitrary. Additionally, contrary to the original Cancitis model (3, 4), we choose that $s \approx 1$ at steady state. Since $s$ is an abstract quantity and only relative changes in $s$ are relevant, we believe that this makes more sense than the choice of $s \approx 3.61$ at steady state in the original Cancitis model. For the choices $s \approx 1$ and $s_{x_0} = \frac{1}{7} \approx 1.4 \times 10^{-1}$ at steady state, the Michaelis-Menten quantity $\frac{s}{s_{x_0}+s}$ approximately takes the value 0.88.
- The parameter values in $\phi_x$ and $\phi_y$ from eq. (S2) are harder to estimate. Changing these parameters over different orders of magnitude shows that to achieve a realistic number of HSCs at a healthy steady state for a value of $p_{x_0}$ between 0 and 1, $c_{xx}$, $c_{xy}$, $c_{yx}$, and $c_{yy}$ in eq. (S2) should be of the order of $10^{-6}$. Inspired by the earlier work with the Cancitis model, (3, 4), we here choose $c_{xx} = 5.6 \times 10^{-6}$, $c_{xy} = 5.4 \times 10^{-6}$, $c_{yx} = 5.2 \times 10^{-6}$, and $c_{yy} = 5.0 \times 10^{-6}$, which is a difference of a factor 10 compared to the original Cancitis model. In the original Cancitis model, it was assumed that the number of HSCs at steady state was $10^4$, but newer results show that a healthy person has 50,000-200,000 stem cells actively contributing to blood cell formation (2), which implies a reduction of $c_{xx}$, $c_{xy}$, $c_{yx}$, and $c_{yy}$ by a factor of 10. It is important to note that $c_{xx} \geq c_{xy} \geq c_{yx} \geq c_{yy}$ since we assume that malignant cells are impacted less by competition than healthy cells and that the malignant cells impact the healthy more than vice versa. For the case of the HSCs being at the healthy steady state in absence of mHSC, i.e. $x_0 = 10^5$ and $y_0 = 0$, the crowding function takes the value $\phi_x(10^5, 0) \approx 0.64$. Thus, the crowding function and the Michaelis-Menten constant which accounts for cytokine effects are of the same order of magnitude.
- For the healthy stem cells, we now only have one parameter left to estimate, the self-renewal fraction, $p_{x_0}$. Using the estimate of $x_0 = 10^5$ at steady state gives $p_{x_0} \approx 0.89$.
- We will now continue with the death rate of the mature cells. Here, we choose to let the mature cells represent the neutrophils. The lifespan of neutrophils is estimated to be 6.6 days (5), and using this, the death rate for the mature cells in our model is $d_{x_2} = \frac{1}{6.6}$ day$^{-1} \approx 1.52 \times 10^{-1}$ day$^{-1}$.
- We now continue with the progenitor cells. There exist different estimates for the number of neutrophil precursors. We choose a progenitor population of the order of $10^6$ cells which is a value between the abundance of HSCs and long-term culture-initiating cells (LTC-ICs) in humans (6). This choice is consistent with estimates in mice, where a population of $1.6 \times 10^4$ stem cells gives rise to a population of $4.0 \times 10^5$ common myeloid progenitors (CMPs) (5). Thus, the compartment size growths with a factor 25 from HSCs to CMPs. If we assume that the ratio between both compartments is the same in humans and in mice, we obtain that the $10^5$ stem cells in humans will give rise to $2.5 \times 10^6$ CMPs in humans.
- We still need to estimate the fractions of self-renewal and differentiation of the progenitor cells, which are difficult to measure. Overall, it is believed that more mature cells divide more often and are more prone to differentiation than self-renewal compared to the stem cells (7). Therefore, we will here assume that the overall proliferation rate of the CMPs is 3 times as large as the proliferation rate of the HSCs, i.e. $\alpha_{x_1} = 3\alpha_{x_0} \approx 1.1 \times 10^{-2}$ day$^{-1}$, and that the self-renewal fraction is half as large for the CMPs as for the HSCs, i.e. $p_{x_1} = \frac{1}{2}p_{x_0} \approx 0.445$. Different choices of these parameters lead to different amplification factors. Key dynamic features of the model, however, do not change.
- Due to lack of information, we arbitrarily choose the death rate of the progenitor cells to be one third of their proliferation rate, i.e. $d_{x_1} = \frac{1}{3}\alpha_{x_1} \approx 3.7 \times 10^{-3}$day$^{-1}$. Thus, on average an HPC will divide 3 times before dying.

- Now, this leaves only $A_{x_0}$ and $A_{x_1}$ undetermined in equations (S1b) and (S1c). Assuming a total number of $2.5 \times 10^6$ (from above) HPCs and $6.4 \times 10^{11}$ HMCs in the healthy steady state (5) gives $A_{x_0} = 3.4 \times 10^1$ and $A_{x_1} = 3.2 \times 10^6$.
- By the principle of parsimony, we will assume that most of the parameters of the malignant cells are equal to those of their healthy counterparts. The *JAK2*-mutated malignant cells have a proliferative advantage (8), and thus, some parameters must be different for the malignant cells. The competition parameters in $\phi_x$ and $\phi_y$ in eq. (S2) are already different for the malignant and the healthy stem cells, but numerical experiments (with the parameter values for the $a$- and $s$-equations described below) show that if all other parameters are the same for the malignant cells as for the healthy cells, it takes more than 300 years for the mHSC compartment to grow from one malignant cell to the same size as the HSC compartment, and thus this growth rate seems unreasonable. Therefore, the mHSCs must have an additional proliferative advantage as well. The most important parameter for determining the proliferative advantage is the self-renewal fraction (9). In this model, the self-renewal fraction for the mHSCs is given by the expression $p_{y_0}\phi_y(y_0, y_0)\frac{s}{s_{y_0}+s}$. Here, we arbitrarily choose that the malignant cells have $p_{y_0} = 0.97 > p_{x_0}$ to ensure a proliferative advantage for the mHSCs in the absence of crowding or cytokine signalling. We also choose to set $s_{y_0} = \frac{1}{2}s_{x_0} = \frac{1}{14} \approx 7.1 \times 10^{-2}$, hereby letting the mHSCs have an even higher fraction self-renewal since they respond more effectively to the cytokine signalling. Similarly to the choice $p_{x_1} = \frac{1}{2}p_{x_0}$, we also choose $p_{y_1} = \frac{1}{2}p_{y_0} = 0.485$.
- Now, we must choose $\alpha_{y_0}$ in such a way that the development of the malignant clone matches data and earlier models. Based on systematic simulations, one such choice is $\alpha_{y_0} = 1.5\alpha_{x_0} = \frac{1.5}{280} = 5.4 \times 10^{-3}\,\text{day}^{-1}$. As we chose $\alpha_{y_0} = 1.5\alpha_{x_0}$, we will also choose $\alpha_{y_1} = 1.5\alpha_{x_1} \approx 1.65 \times 10^{-2}\,\text{day}^{-1}$. With this choice of parameters, it takes the mHSC compartment approximately 31.5 years to go from one malignant stem cell to the same size as the HSC compartment, and it takes approximately 10.5 years to go from a *JAK2* VAF of 1% to a *JAK2* VAF of 33%. This time span is between other estimates of the growth rate of *JAK2* clones (10, 11), but we do note that a large interindividual variability for the growth rates of *JAK2* clones has been reported (11).
- Finally, we must choose the parameters for equations (S1g) and (S1h). These are more arbitrary than most other parameters, and here we choose $e_s = 7.2 \times 10^1\,\text{day}^{-1}$ and $I = 2\,\text{day}^{-1}$ which are scaled versions of the values used the original Cancitis model (12), taking into account that we here use $s \approx 1$ instead of $s \approx 3.61$. However, we choose different values of $e_a$ and $r_s$, namely $e_a = 1.2 \times 10^8\,\text{day}^{-1}$ and $r_s = 8.6 \times 10^{-2}\,\text{day}^{-1}$. These are quite different from the values in the original Cancitis model ($e_a = 2 \times 10^5\,\text{day}^{-1}$ and $r_s = 3 \times 10^{-4}\,\text{day}^{-1}$), but this choice makes sure that given the rest of the parameters, $s$ reaches the assumed steady state value of $s \approx 1$ as we assumed earlier, and $a$ reaches a steady state value of approximately $8.4 \times 10^2$ at the healthy steady state.

Note that the healthy steady state in the main part of the paper with $1.0 \times 10^5$ stem cells gives very slightly different numbers of healthy cells at steady state than the values used for the parameterisation above. This is due to rounding errors when choosing the parameters. An overview of the parameter values can be found in table S1.

## 2 SENSITIVITY ANALYSIS

We now present a simple sensitivity analysis of the parameters of the model in eq. (S1). We do this by in turn increasing/decreasing each parameter by 10% of its standard value given in table S1 and looking at the resulting changes. We are mostly interested in the parameters' effects on the development of the *JAK2*

| Parameter | Description | Value | Unit | Source |
|---|---|---|---|---|
| $\alpha_{x_0}$ | Proliferation rate of HSCs | $3.6 \times 10^{-3}$ | day$^{-1}$ | (1, 2) |
| $\alpha_{y_0}$ | Proliferation rate of mHSCs | $5.4 \times 10^{-3}$ | day$^{-1}$ | Estimated |
| $p_{x_0}$ | self-renewal fraction for HSCs | 0.89 | 1 | Estimated |
| $p_{y_0}$ | self-renewal fraction for mHSCs | 0.97 | 1 | Chosen |
| $c_{xx}$ | Constant for HSCs inhibiting HSC self-renewal | $5.6 \times 10^{-6}$ | 1 | Estimated |
| $c_{yx}$ | Constant for HSCs inhibiting mHSC self-renewal | $5.2 \times 10^{-6}$ | 1 | Estimated |
| $c_{xy}$ | Constant for mHSCs inhibiting HSC self-renewal | $5.4 \times 10^{-6}$ | 1 | Estimated |
| $c_{yy}$ | Constant for mHSCs inhibiting mHSC self-renewal | $5.0 \times 10^{-6}$ | 1 | Estimated |
| $s_{x_0}$ | Half-saturation constant for cytokine signal for HSCs | $1.4 \times 10^{-1}$ | 1 | Chosen |
| $s_{y_0}$ | Half-saturation constant for cytokine signal for mHSCs | $7.1 \times 10^{-2}$ | 1 | Chosen |
| $A_{x_0}$ | Amplification factor from HSCs to HPCs | $3.4 \times 10^{1}$ | 1 | Estimated |
| $A_{y_0}$ | Amplification factor from mHSCs to mHPCs | $3.4 \times 10^{1}$ | 1 | Estimated |
| $\alpha_{x_1}$ | Proliferation rate of HPCs | $1.1 \times 10^{-2}$ | day$^{-1}$ | Chosen |
| $\alpha_{y_1}$ | Proliferation rate of mHPCs | $1.7 \times 10^{-2}$ | day$^{-1}$ | Chosen |
| $p_{x_1}$ | self-renewal fraction for HPCs | 0.445 | 1 | Chosen |
| $p_{y_1}$ | self-renewal fraction for mHPCs | 0.485 | 1 | Chosen |
| $d_{x_1}$ | Death rate of HPCs | $3.7 \times 10^{-3}$ | day$^{-1}$ | Chosen |
| $d_{y_1}$ | Death rate of mHPCs | $3.7 \times 10^{-3}$ | day$^{-1}$ | Chosen |
| $A_{x_1}$ | Amplification factor from HPCs to HMCs | $3.2 \times 10^{6}$ | 1 | Estimated |
| $A_{y_1}$ | Amplification factor from mHPCs to mHMCs | $3.2 \times 10^{6}$ | 1 | Estimated |
| $d_{x_2}$ | Death rate of HMCs | $1.5 \times 10^{-1}$ | day$^{-1}$ | (5) |
| $d_{y_2}$ | Death rate of mHMCs | $1.5 \times 10^{-1}$ | day$^{-1}$ | (5) |
| $e_a$ | Degradation constant for $a$ | $1.2 \times 10^{8}$ | day$^{-1}$ | Estimated |
| $r_s$ | Production constant for $s$ | $8.6 \times 10^{-2}$ | day$^{-1}$ | (4) |
| $e_s$ | Degradation constant for $s$ | $7.2 \times 10^{1}$ | day$^{-1}$ | Estimated |
| $I$ | External up-regulation of $s$ | 2 | day$^{-1}$ | (4) |

**Table S1.** Parameter values for the model in equations (S1) and (S2).

VAF, and therefore we first plot the relative change in the time from one malignant cancer cell to a *JAK2* VAF of 50%. These results can be seen in figures S1 and S2. Next, we plot the time evolution of the stem cells, progenitor cells, mature cells, and *JAK2* VAF over time for the different parameter variations. These results can be seen in figs. S3 to S10. In each simulation, we use the standard parameters from table S1 except for the one which is increased/decreased by 10%, and the simulation starts from the healthy steady state with one added malignant cell, i.e the initial conditions are $x_0(0) = 1.0 \times 10^5$, $x_1(0) = 2.5 \times 10^6$, $x_2(0) = 6.4 \times 10^{11}$, $y_0(0) = 1$, $y_1(0) = 0$, $y_2(0) = 0$, $a(0) = 8.1 \times 10^2$, and $s(0) = 1$. In the plots with cells, the solid green curves represent the number of healthy cells, the solid red curves represent the number of malignant cells, and the dashed black curves represent the sum of healthy and malignant cells. From figures S1 to S10, it is clear that the most sensitive parameters of the model are $p_{x_0}$ and $p_{y_0}$ followed by $c_{xx}$, $c_{yx}$, $\alpha_{y_0}$, and $s_{x_0}$. These parameters are the most sensitive because they, except $\alpha_{y_0}$, determine the self-renewal fraction of the healthy and the malignant stem cells, the products $p_{x_0}\phi_x(x_0, y_0)\frac{s}{s_{x_0}+s}$ and $p_{y_0}\phi_y(x_0, y_0)\frac{s}{s_{y_0}+s}$ respectively, and that the self-renewal is the main contributor to the overall fitness of each of the cell types (9). Unfortunately, these are also parameters that are determined arbitrarily. However, they are chosen as a coherent set of parameters, and not as individual values, in such a way that the model produces both a reasonable number of healthy cells at steady state and a reasonable growth rate of the

malignant clone as explained in section S1. Thus, we should not put too much emphasis on the sensitivities of these parameters when affecting each on them individually, but rather on the effect of affecting all of them together in such a way that the healthy steady state, growth rate of malignant clone, etc. are similar. Investigating this is non-trivial and comprehensive work, and here we refrain from investigating it in detail. The reason why $\alpha_{y_0}$ is also sensitive is that it determines the proliferation rate of the mHSCs. Thus, it contributes to determining how quickly the mHSC compartment grows and therefore also in turn how quickly the *JAK2* VAF grows.

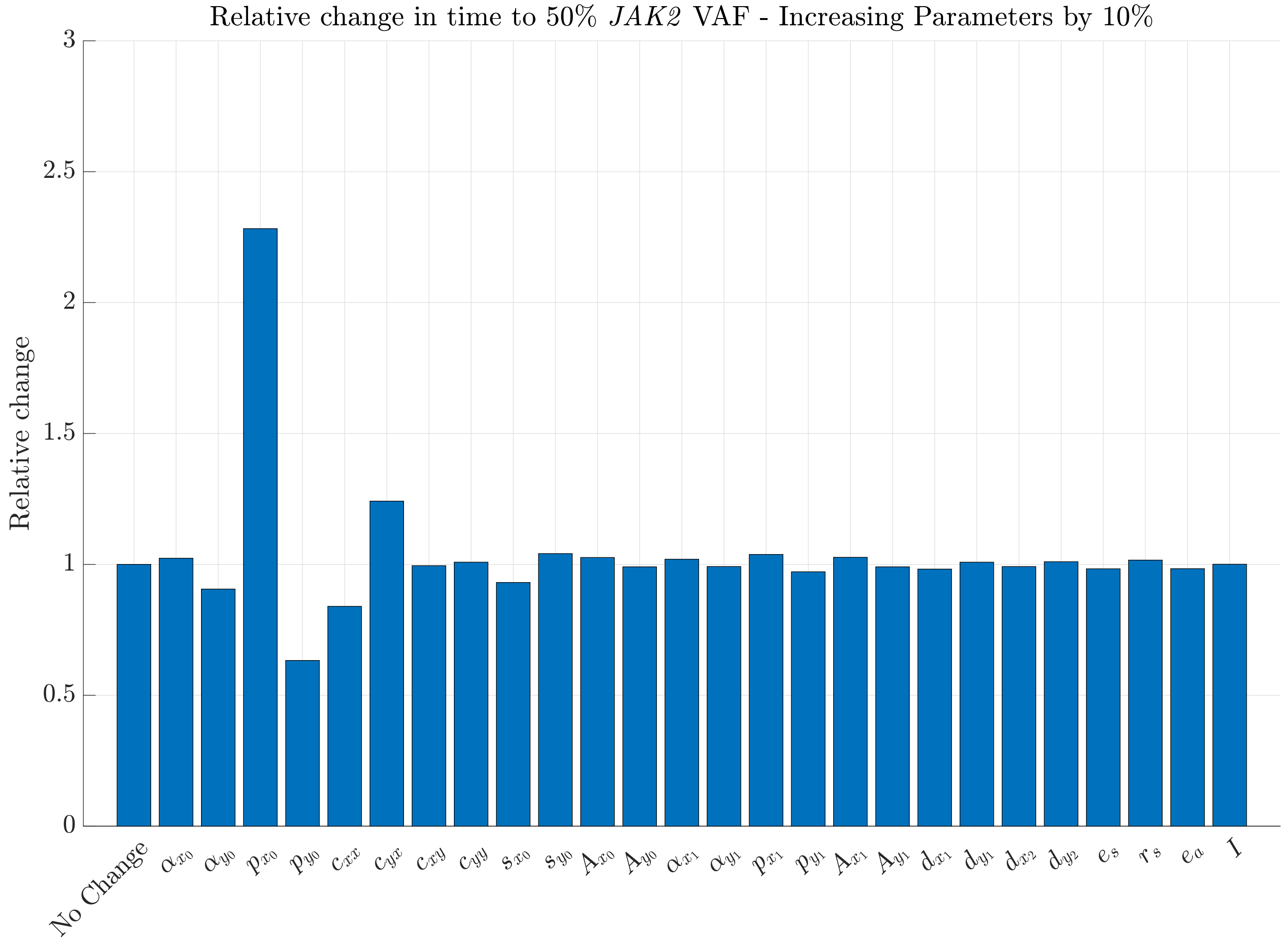


Figure S1: Relative change in time from one malignant cancer cell to a *JAK2* VAF of 50% when varying the parameters. The parameters are in turn increased by 10% of their standard value in table S1.

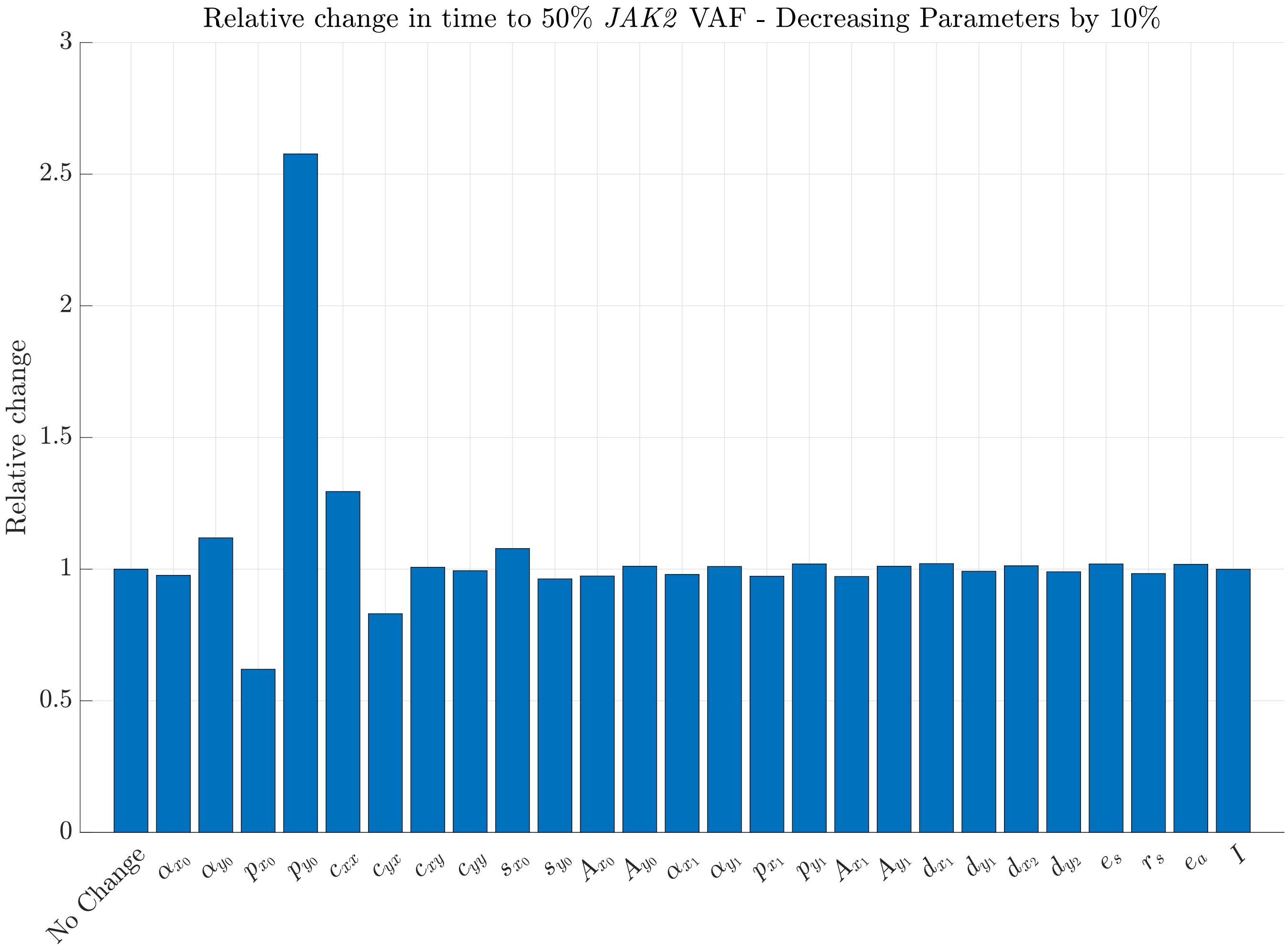


Figure S2: Relative change in time from one malignant cancer cell to a *JAK2* VAF of 50% when varying the parameters. The parameters are in turn decreased by 10% of their standard value in table S1.

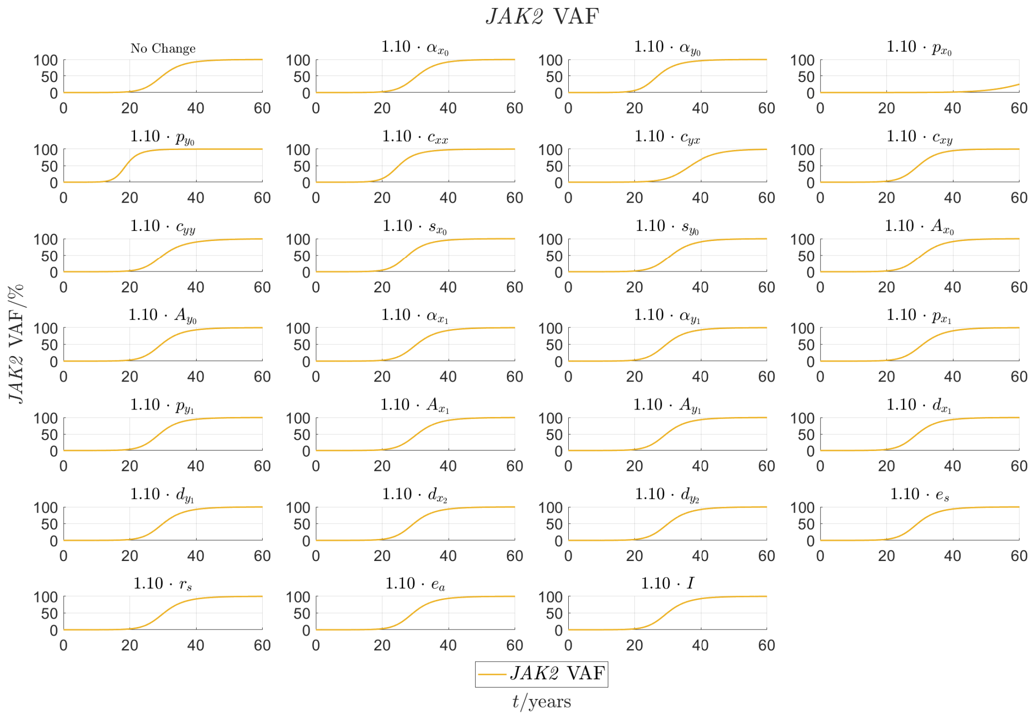


Figure S3: Plots of the *JAK2* VAF when varying the parameters. The parameters are in turn decreased by 10% of their standard value in table S1 as indicated in the plot titles.

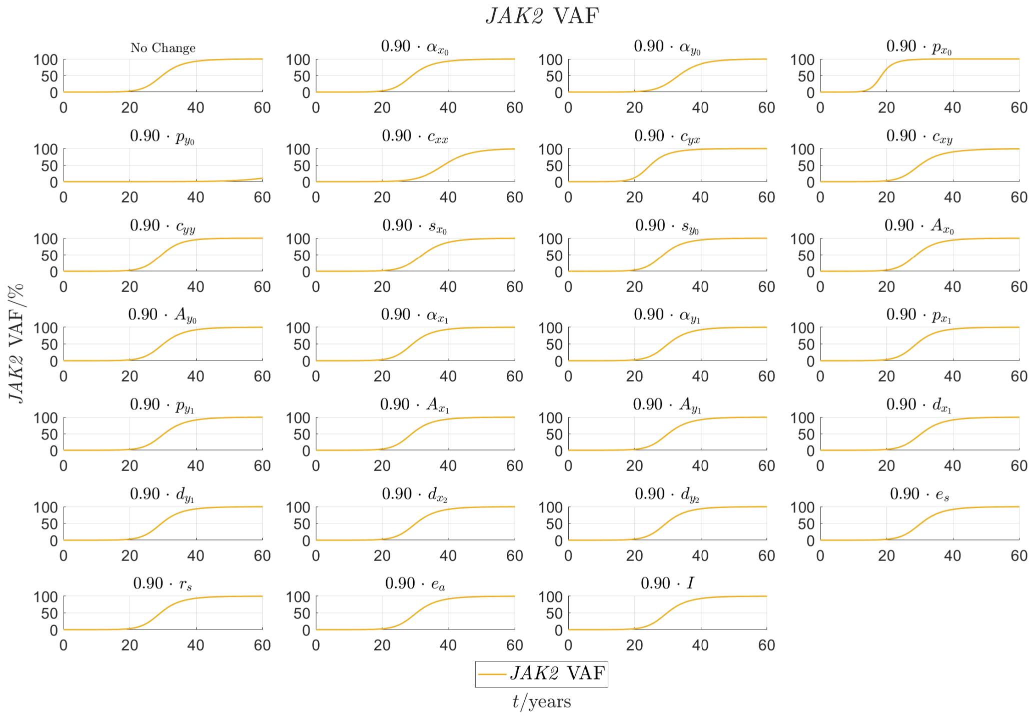


Figure S4: Plots of the *JAK2* VAF when varying the parameters. The parameters are in turn decreased by 10% of their standard value in table S1 as indicated in the plot titles.

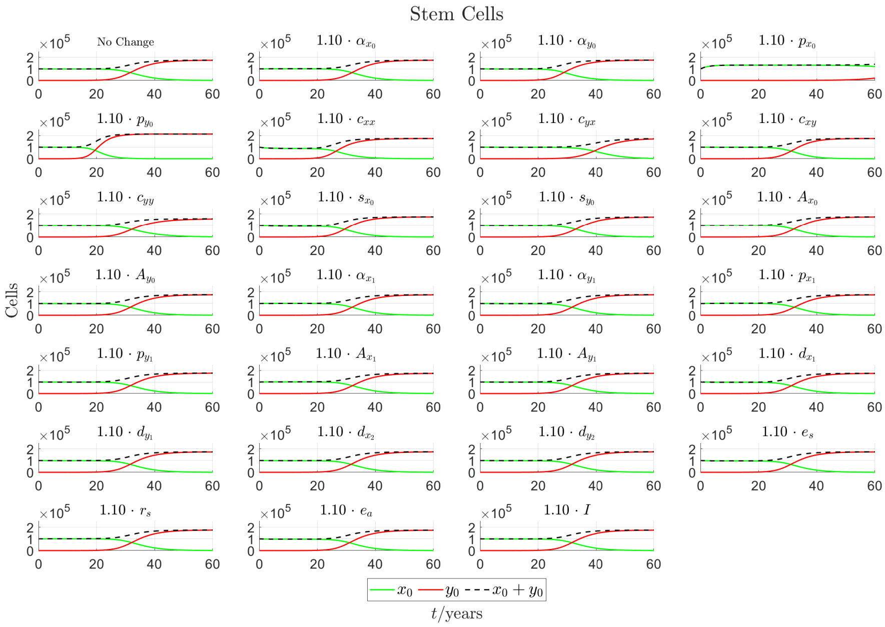

Figure S5: Plots of the stem cells when varying the parameters. The parameters are in turn increased by 10% of their standard value in table S1 as indicated in the plot titles. The solid green curves represent the number of healthy cells, the solid red curves represent the number of malignant cells, and the dashed black curves represent the sum of healthy and malignant cells.

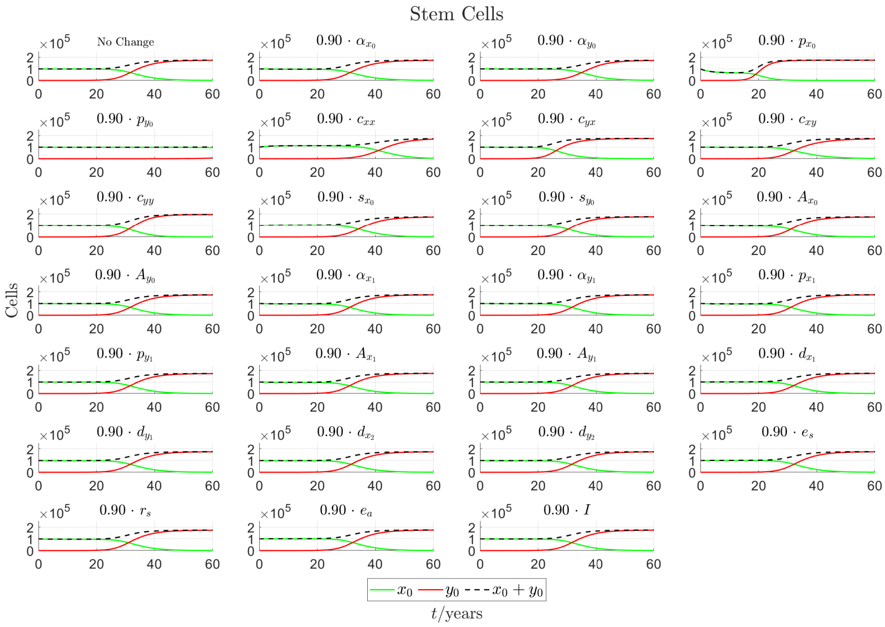

Figure S6: Plots of the stem cells when varying the parameters. The parameters are in turn decreased by 10% of their standard value in table S1 as indicated in the plot titles. The solid green curves represent the number of healthy cells, the solid red curves represent the number of malignant cells, and the dashed black curves represent the sum of healthy and malignant cells.

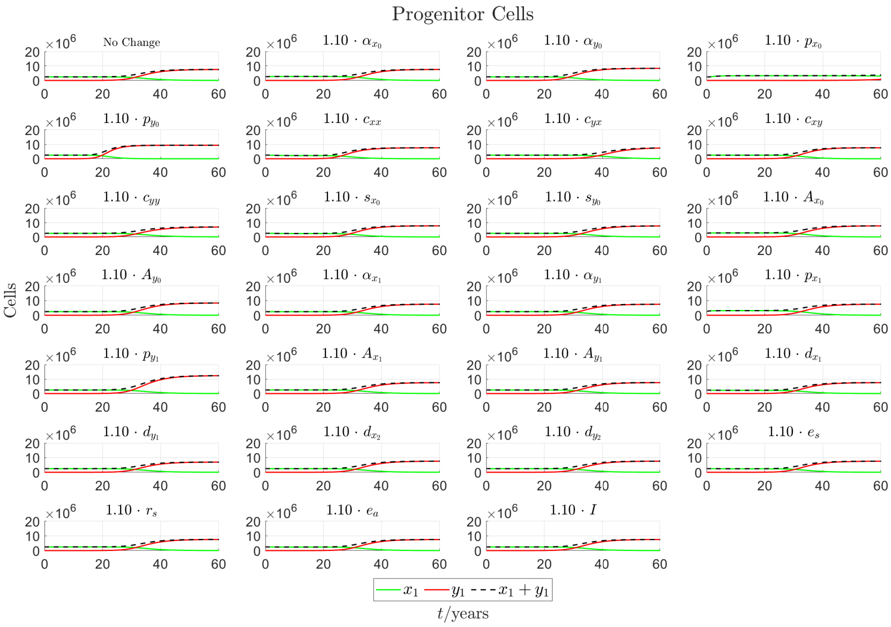


Figure S7: Plots of the progenitor cells when varying the parameters. The parameters are in turn decreased by 10% of their standard value in table S1 as indicated in the plot titles. The solid green curves represent the number of healthy cells, the solid red curves represent the number of malignant cells, and the dashed black curves represent the sum of healthy and malignant cells.

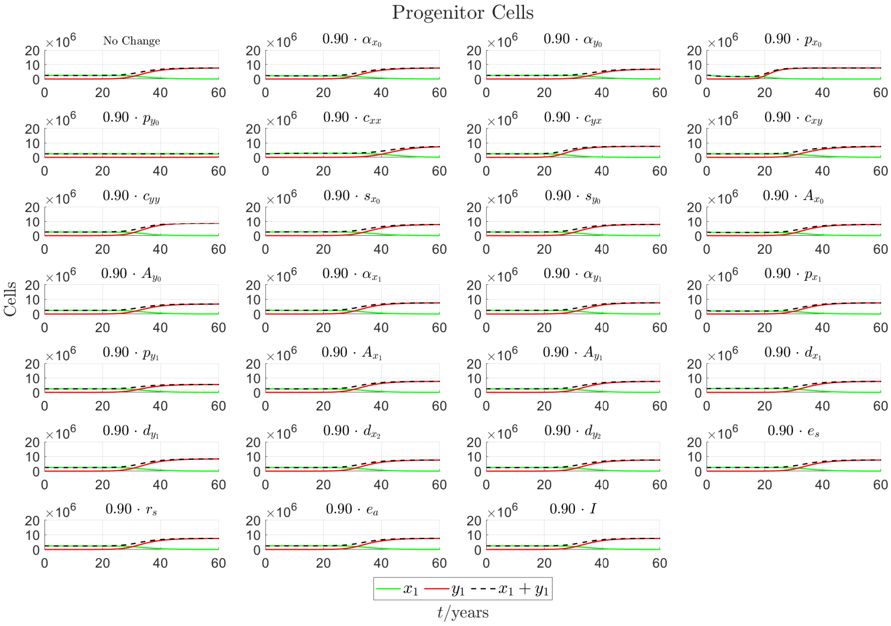


Figure S8: Plots of the progenitor cells when varying the parameters. The parameters are in turn decreased by 10% of their standard value in table S1 as indicated in the plot titles. The solid green curves represent the number of healthy cells, the solid red curves represent the number of malignant cells, and the dashed black curves represent the sum of healthy and malignant cells.

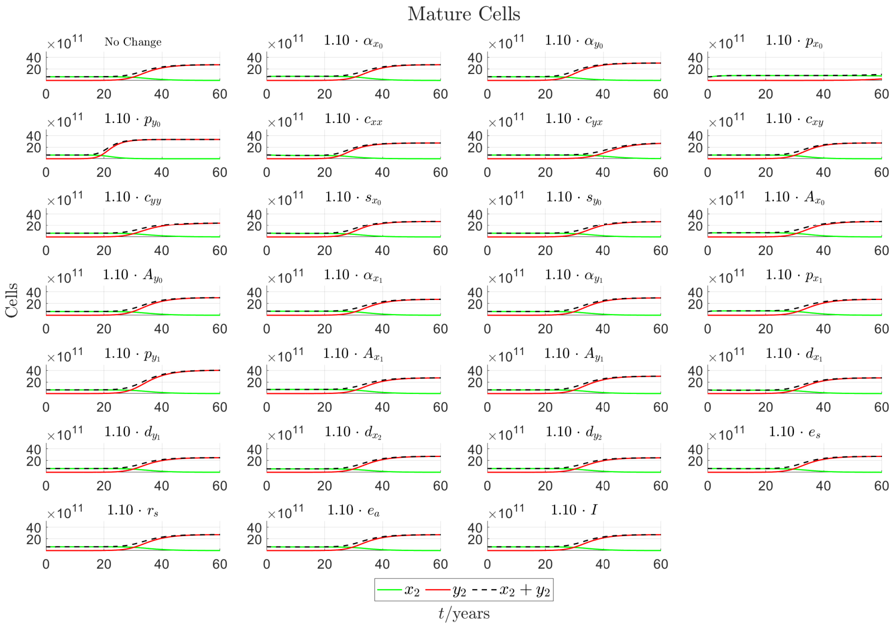


Figure S9: Plots of the mature cells when varying the parameters. The parameters are in turn decreased by 10% of their standard value in table S1 as indicated in the plot titles. The solid green curves represent the number of healthy cells, the solid red curves represent the number of malignant cells, and the dashed black curves represent the sum of healthy and malignant cells.

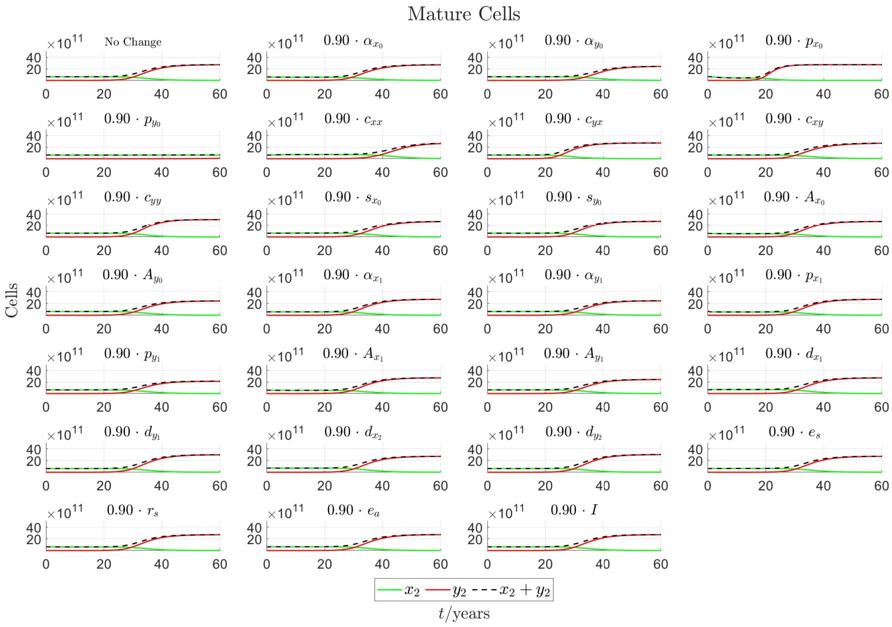


Figure S10: Plots of the mature cells when varying the parameters. The parameters are in turn decreased by 10% of their standard value in table S1 as indicated in the plot titles. The solid green curves represent the number of healthy cells, the solid red curves represent the number of malignant cells, and the dashed black curves represent the sum of healthy and malignant cells.

# 3 NUMERICAL EXPERIMENTS WITH TREATMENT EFFECTS

In this section, we present plots of the model simulations with different parameters for the malignant cells changing after 30 years to test possible treatment effects. From these simulations it seems clear that to achieve sustained reductions in the *JAK2* VAF, a given patient must achieve reductions in the number of mHSCs (relative to the HSCs), and this can only be realised by the treatment affecting the self-renewal fraction, i.e. affecting one of the parameters of the expression $p_{y_0}\phi_y(y_0, y_0)\frac{s}{s_{y_0}+s}$.

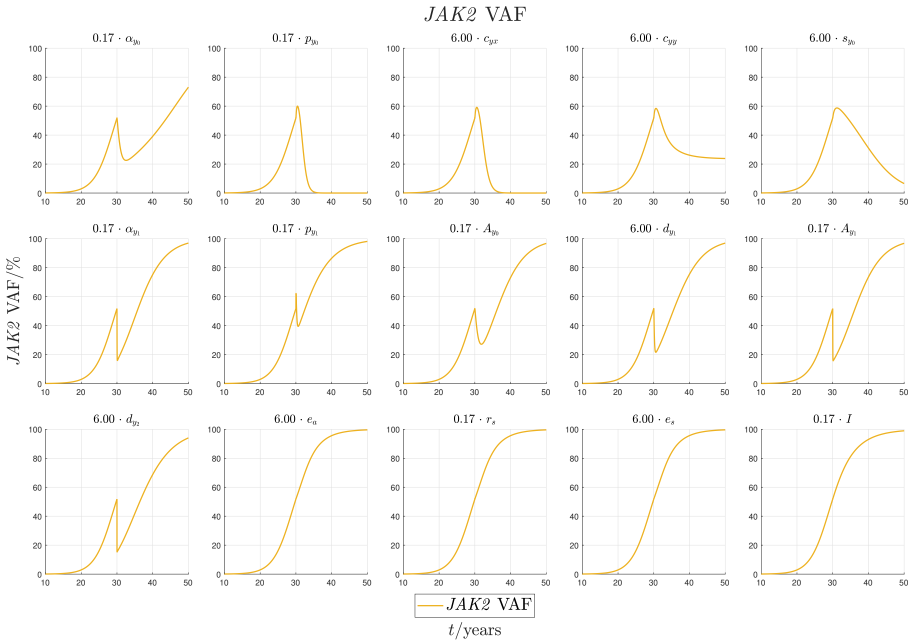


Figure S11: Plots of the *JAK2* VAF when changing one parameter after 30 years. The parameter changed is indicated in the plot titles.

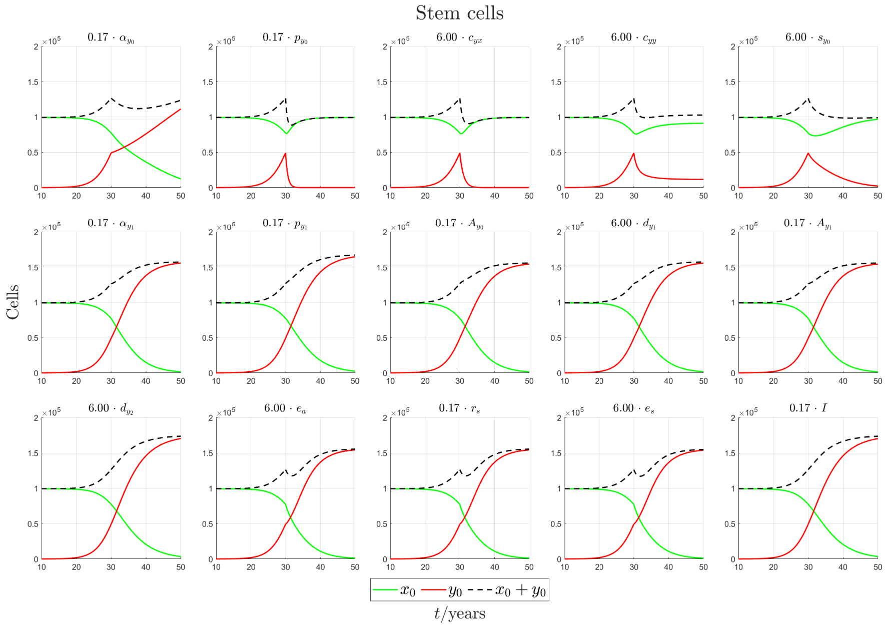


Figure S12: Plots of the stem cells when changing one parameter after 30 years. The parameter changed is indicated in the plot titles. The solid green curves represent the number of healthy cells, the solid red curves represent the number of malignant cells, and the dashed black curves represent the sum of healthy and malignant cells.

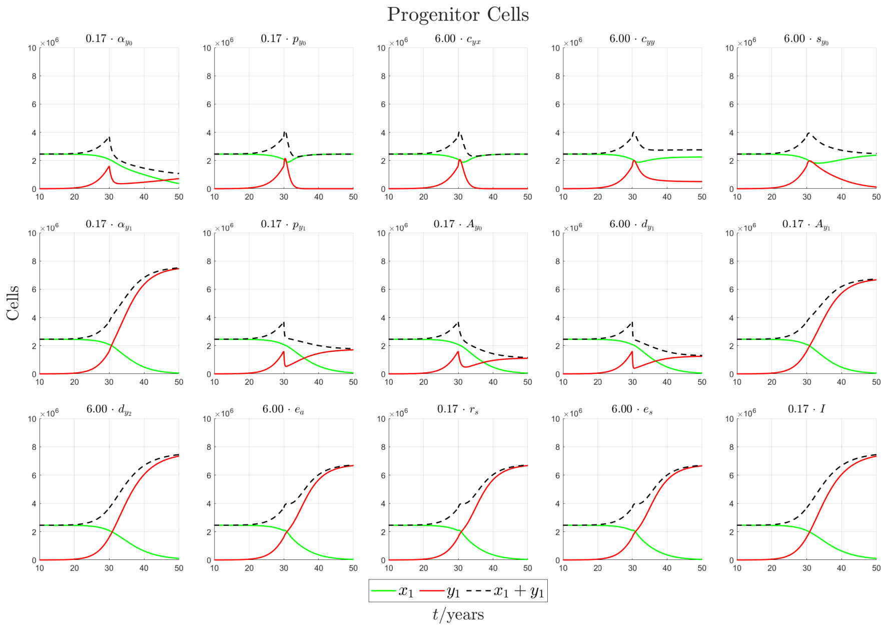


Figure S13: Plots of the progenitor cells when changing one parameter after 30 years. The parameter changed is indicated in the plot titles. The solid green curves represent the number of healthy cells, the solid red curves represent the number of malignant cells, and the dashed black curves represent the sum of healthy and malignant cells.

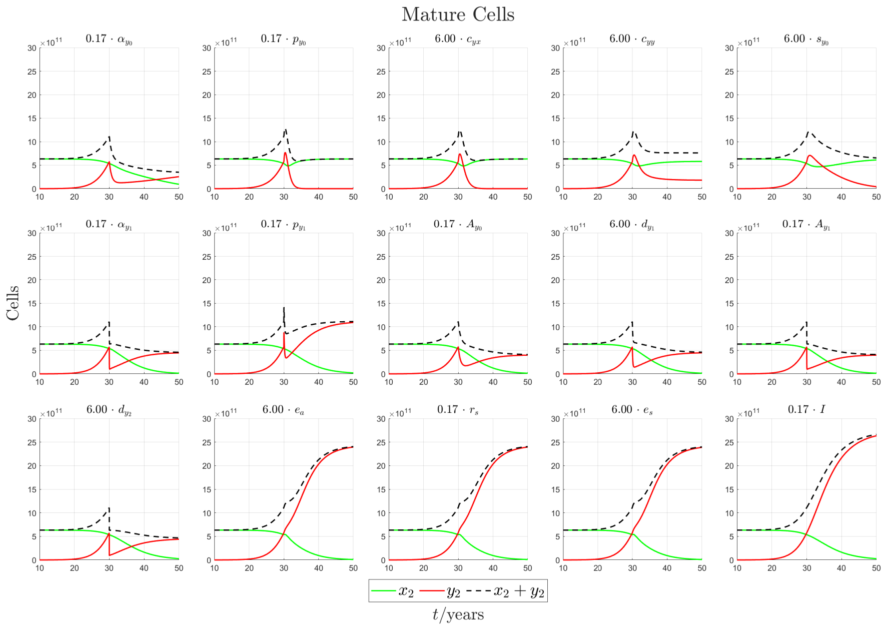


Figure S14: Plots of the mature cells when changing one parameter after 30 years. The parameter changed is indicated in the plot titles. The solid green curves represent the number of healthy cells, the solid red curves represent the number of malignant cells, and the dashed black curves represent the sum of healthy and malignant cells.

## 4 NONLINEAR LEAST SQUARES DATA FITTING IN ODES

The following description of the nonlinear least squares data fitting problem for ODEs is largely based on the lectures notes (13) and the corresponding slides (by John Bagterp Jørgensen) from the 2019 edition of course 02610 Optimisation and Data Fitting at the Technical University of Denmark which the first author, T.I.B., followed. In the following, $x$ and $y$ are general vector quantities and thus do not refer to the variables from the model in eq. (S1).

Consider the case where we at the $m$ time points, $\{t_i\}_{i=1}^m$, have the $m$ data points, $\{y_i\}_{i=1}^m$. These data points are measurements of some time-dependent quantity, $\hat{y}(t;\rho)$, that depends on the vector of parameters, $\rho$, and with added noise, $e(t)$. We will assume that this noise is normally distributed with mean 0 and variance $\sigma^2$, i.e. we have

$$y_i = \hat{y}(t_i;\rho) + e(t_i), \quad e(t_i) \sim N(0,\sigma^2). \tag{S3}$$

In our case, $\{y_i\}_{i=1}^m$ is the collection of *JAK2* VAF measurements for a given patient, and $\{\hat{y}(t_i;\rho)\}_{i=1}^m$ are the model predictions of the *JAK2* VAF for the given patient. In the following, we want to find the value of the parameter vector, $\rho$, that minimises the deviation between the measurements, $\{y_i\}_{i=1}^m$, and the model predictions, $\{\hat{y}(t_i;\rho)\}_{i=1}^m$. In our case, $\rho$ will be the vector that describes the effects of RUX on a given patient, i.e. $\rho = (\rho_{s_{y_0}}, \rho_{d_{y_1}})$. We will find the optimal value of $\rho$ using a least squares objective function, and the entire problem is described in eqs. (S4a) to (S4e). An explanation of each equation follows below:

$$\min_{\rho\in\mathbb{R}^{n_\rho}} \phi = \frac{1}{2}\sum_{i=1}^{m} \|y_i - \hat{y}(t_i;\rho)\|_2^2, \tag{S4a}$$

$$\text{s.t.} \quad \frac{\mathrm{d}x}{\mathrm{d}t}(t) = f(t,x(t);\rho), \tag{S4b}$$

$$x(t_0) = x_0, \tag{S4c}$$

$$\hat{y}(t;\rho) = g(x(t),\rho), \tag{S4d}$$

$$\rho_l \le \rho \le \rho_u. \tag{S4e}$$

Equation (S4a) describes the nonlinear least squares objective function that we want to minimise. The factor of $\frac{1}{2}$ is included for historical and conventional reasons, but it makes no difference for the optimal value of $\rho$. $\rho$ has dimension $n_\rho$. In our case, we have $\rho = (\rho_{s_{y_0}}, \rho_{d_{y_1}})$ for determining a patient's response to the treatment in terms of changes to $s_{y_0}$ and $d_{y_1}$, respectively, and $n_\rho = 2$. Equation (S4b) describes that the variables, $x(t)$, of the system are governed by some system of differential equations, $f(t,x(t);\rho)$. In our case, $x(t)$ will be the vector consisting of the variables $x_0(t)$, $x_1(t)$, $x_2(t)$, $y_0(t)$, $y_1(t)$, $y_2(t)$, $a(t)$, and $s(t)$, and $f(t,x(t),\rho)$ will be the system of ODEs given in eq. (S1) with modifications to the parameters affected by the RUX treatment, i.e. using $\widetilde{s_{y_0}} = (1 + c_R(t)\rho_{s_{y_0}})s_{y_0}$ and $\widetilde{d_{y_1}} = (1 + c_R(t)\rho_{d_{y_1}})d_{y_1}$ instead of $s_{y_0}$ and $d_{y_1}$, respectively, during treatment (see equations (3) and (4) of the main text). Equation (S4c) describes the initial condition for the variables. How these are chosen will be described later. Equation (S4d) describes that the model estimate for the measurements, $\hat{y}(t;\rho)$, is given by some function, $g(x(t);\rho)$, of the variables, $x(t)$. In our case, we have $g(x(t)) = \frac{y_2(t)}{x_2(t)+y_2(t)}$ as described in the main text. Finally, eq. (S4e) describes that the values of $\rho$ are (or might be) constrained within some interval, here denoted by the bounds $\rho_l$ and $\rho_u$. In our model, we have $0 \le \rho_{s_{y_0}} < \infty$ and $0 \le \rho_{d_{y_1}} < \infty$. When quantifying the quality of the fits, we will use the root mean square error (RMSE) instead of the sum of squared errors in

eq. (S4a). The RMSE is given by

$$\text{RMSE} = \sqrt{\frac{1}{m}\sum_{i=1}^{m} \|y_i - \hat{y}(t_i;\rho)\|_2^2}. \tag{S5}$$

The RMSE is easier to interpret than the sum of squared errors since, due to the square root, it has the same unit as the data themselves. Additionally, instead of giving the sum of all errors, it gives a measure for the typical error between the model and the data. Finally, dividing by the number of data points, $m$, and taking the square root does not alter the optimal value of $\rho$, and thus the optimal values of both the sum of squared errors in eq. (S4a) and the RMSE are obtained for the same value of $\rho$. However, the sum of squared errors in eq. (S4a) has properties that make it easier to optimise than the RMSE, and therefore we will choose to optimise the sum of squared errors and report the RMSE.

Let $\hat{\rho}$ be the estimated parameter vector and $\rho^*$ the true parameter vector. Then, the residuals from the optimisation problem in eq. (S4) are defined by

$$r_i = y_i - \hat{y}(t_i;\hat{\rho}), \quad i = 1, 2, \ldots, m. \tag{S6}$$

To quantify the uncertainties in the fitting procedure, we are interested in knowing how $r_i$ changes when we adjust $\rho$. More formally, we are interested in in calculating the derivatives $\frac{\partial r_i}{\partial \rho_j} = -\frac{\partial \hat{y}}{\partial \rho_j}$. We can collect all the residuals in a vector and all the derivatives of the residuals with respect to the parameters in a matrix, called the Jacobian, to get

$$r(\rho) = \begin{bmatrix} r_1(\rho) \\ r_2(\rho) \\ \vdots \\ r_m(\rho) \end{bmatrix}, \tag{S7}$$

$$J(\rho) = \begin{bmatrix} \frac{\partial \hat{y}}{\partial \rho_1}(t_1;\rho) & \frac{\partial \hat{y}}{\partial \rho_2}(t_1;\rho) & \cdots & \frac{\partial \hat{y}}{\partial \rho_{n_\rho}}(t_1;\rho) \\ \frac{\partial \hat{y}}{\partial \rho_1}(t_2;\rho) & \frac{\partial \hat{y}}{\partial \rho_2}(t_2;\rho) & \cdots & \frac{\partial \hat{y}}{\partial \rho_{n_\rho}}(t_2;\rho) \\ \vdots & \vdots & \ddots & \vdots \\ \frac{\partial \hat{y}}{\partial \rho_1}(t_m;\rho) & \frac{\partial \hat{y}}{\partial \rho_2}(t_m;\rho) & \cdots & \frac{\partial \hat{y}}{\partial \rho_{n_\rho}}(t_m;\rho) \end{bmatrix}. \tag{S8}$$

Additionally, we define:

$$H = H(\hat{\rho}) = J(\hat{\rho})^T J(\hat{\rho}). \tag{S9}$$

When $\hat{\rho}$ is reasonably close to $\rho^*$, we may use the approximation

$$\hat{y}(t;\hat{\rho}) \approx \hat{y}(t;\rho^*) + J(\rho^*)(\hat{\rho} - p^*). \tag{S10}$$

An unbiased estimate of the noise variance is given by

$$\hat{\sigma}^2 = \frac{1}{m - n_\rho} \sum_{i=1}^{m} (y_i - \hat{y}(t_i;\hat{\rho}))^2 . \tag{S11}$$

Now, using eqs. (S9) to (S11), it can be shown (13) that $\hat{\rho}$ approximately follows a normal distribution with mean $p^*$ and variance $\hat{\sigma}^2 H^{-1}$, i.e.

$$\hat{\rho} \sim N(\rho^*, \hat{\sigma}^2 H^{-1}). \tag{S12}$$

Note that his results only holds asymptotically for $m$ large enough. From eq. (S12), it can be shown (13) that an approximate $100(1-\alpha)\%$ confidence interval (CI) for the parameters is given by

$$\hat{\rho}_i \pm t_{m-n_\rho}\left(\frac{\alpha}{2}\right) \hat{\sigma}\sqrt{C_{ii}}, \tag{S13a}$$

$$C_{ii} = [H^{-1}]_{ii}, \tag{S13b}$$

where $t_{m-n_\rho}(\frac{\alpha}{2})$ is the probability density function of the $t$-distribution with $m - n_\rho$ degrees of freedom evaluated at $\frac{\alpha}{2}$.

To calculate an approximate 95% CI for the model output of the *JAK2* VAF, we sample 1000 pairs of the parameters from their estimated approximate joint distribution in eq. (S12), then simulate the model with the sampled parameters, and finally take the middle 95% predicted *JAK2* VAF values of these simulations. The sampling procedure can produce negative values of the parameters, in which case we choose to resample the corresponding samples. This is a problem when the estimate of $\rho_{s_{y_0}}$ and/or $\rho_{d_{y_1}}$ is 0 or is close to 0 (compared to its standard deviation), in which case the approximate distribution in eq. (S12) is questionable, and many resamples may be needed. An alternative would be to use the approximate CI for the model predictions given by

$$\hat{y}(t;\hat{\rho}) \pm t_{m-n_\rho}\left(\frac{\alpha}{2}\right) \hat{\sigma} \left( \left[\frac{\partial \hat{y}}{\partial \rho}(t;\hat{\rho})\right]^T H^{-1} \left[\frac{\partial \hat{y}}{\partial \rho}(t;\hat{\rho})\right] \right). \tag{S14}$$

However, as we, due to accuracy requirements in the ODE solvers for this model, can only calculate $\frac{\partial \hat{y}}{\partial \rho}(t;\hat{\rho})$ numerically at the time points at which we have the data, this would only provide point estimates of the CIs. Additionally, eq. (S14) is also an asymptotic approximation. As the approximate normal distribution of $\rho$ in eq. (S12) only holds asymptotically for large enough $m$, we choose to only compute the approximate 95% CI using the sampling technique in the fits in which all available data points are used, though this may still be for small values of $m$ for some patients.

Note that both the sampling technique and eq. (S14) gives an approximate CI for the model output, not the predictions. Thus, it is not necessarily expected that the measurements including the measurement errors falls within this CI most of the time, but that the underlying model output falls within this CI most of the time. This is especially important when considering the initial conditions. As the initial conditions for the optimisation problem in eq. (S4) are assumed to be given and do not depend on $\rho$, the model output at time $t_0$ will always be given by the initial conditions, and the resulting CI at time $t_0$ will have width 0.

However, due to model and measurement errors, the measurements might still be different from the initial conditions used. Examples of this can be seen in section 3 of the main text and sections S6 and S7.

In the simplest of cases, the system of differential equations in eq. (S4b) with initial conditions in eq. (S4c) can be solved analytically, and we can obtain an analytical expression for $\hat{y}(t;\rho)$ via eq. (S4d). However, in practice, we will almost always have to solve the system of differential equations in eq. (S4b) with initial conditions in eq. (S4c) numerically. In this case, the derivatives in $\frac{\partial \hat{y}}{\partial \rho}(t;\hat{\rho})$ must also be calculated numerically. Here we have chosen to do so using final differences with a step size of $10^{-4}$.

In section 3 of the main text and sections S6 and S7, we present the results of solving the problem in eqs. (S4a) to (S4e) for the case of the model presented in eq. (S1) with patient-specific parameters determining the response to the treatment as described in section 2.1.3 of the main text, fitted to individual patients' data as described in sections 2.2 and 2.3 of the main text. For solving the problem in eqs. (S4a) to (S4e) numerically we use the `trust-region-reflexive` algorithm as implemented in MATLAB's `lsqnonlin` [1]. For solving the system of differential equations numerically, we will use MATLAB's `ode15s` [2].

We choose to fit the model to the *JAK2* VAF as described in the main text using the general nonlinear least-squares framework described above. The initial condition for the system of differential equations in eq. (S1) used in the fit is chosen to give the best possible fits in the following way: The model in eq. (S1) is solved from time $t = 0$ to time $t = 80 \times 365$ (80 years) with the standard parameters given in table S1 and without treatment. The initial conditions for this solution are chosen to approximately correspond to the healthy steady state (see section 2.1.2 of the main text for more details) with one additional mHSC added, i.e. $x_0(0) = 1.0 \times 10^5$, $x_1(0) = 2.5 \times 10^6$, $x_2(0) = 6.4 \times 10^{11}$, $y_0(0) = 1$, $y_1(0) = 0$, $y_2(0) = 0$, $a(0) = 8.1 \times 10^2$, and $s(0) = 1$. This produces a discrete sequence of values of all the variables at the different discrete time points of the numerical solution which we will refer to as the "master curve". From these, a corresponding sequence of the *JAK2* VAF at these time points can be calculated as $\frac{y_2(t)}{x_2(t)+y_2(t)}$, and numerical experiments show that this sequence ranges from 0 to 0.9999. As the *JAK2* VAF is monotonically increasing for the standard parameter values, each value of the *JAK2* VAF corresponds to unique values of the 8 variables. Thus, to find the best fit for a given patient, we could in principle solve the nonlinear least-squares optimisation problem in eq. (S4) for every single set of variable values from the master curve and choose the one which provided the best fit according to some measure as our optimal fit. However, this would be computationally very expensive. Instead, for a given patient we define time $t = 0$ for the fit as the time of the first measurement of *JAK2* VAF. Denote this value $g_0$ (measured as a decimal number and not a percentage). Then, we solve the optimisation problem in eq. (S4) for initial values of the variables of the model in eq. (S1) corresponding to values of the initial *JAK2* VAF starting from $\max(g_0 - 0.2, 0)$ and incrementing by 0.01 until reaching a value of $\min(g_0 + 0.2, 1)$. Then, the fit with the smallest RMSE-value is picked as the optimal fit. This procedure is repeated for every patient for which we have data.

When solving the optimisation problem numerically, we use the initial parameter values $(\rho_{s_{y_0}}^{(0)}, \rho_{d_{y_1}}^{(0)}) = (0, 0)$, and numerical experiments show changing this initial value only slightly influences the optimal values found numerically for most patients. For example, using the initial values $(1, 1)$ instead gives very similar fits with the largest difference in RMSE being approximately $6.3 \times 10^{-2}\%$ (relatively), mean difference being $2.6 \times 10^{-3}\%$ (relatively), and 0 patients having a relative difference greater than $0.1\%$.

[1] `https://www.mathworks.com/help/optim/ug/lsqnonlin.html`

[2] `https://www.mathworks.com/help/matlab/ref/ode15s.html`

Further, the largest differences in $\rho_{s_{y_0}}$ and $\rho_{d_{y_1}}$ are $33\%$ and $16\%$ respectively, the mean differences are $2.3\%$ and $0.65\%$ respectively, and only 4 and 21 patients have relative differences greater than $0.1\%$ respectively.

## 5 FIGURES RELATED TO THE OPTIMAL FITS

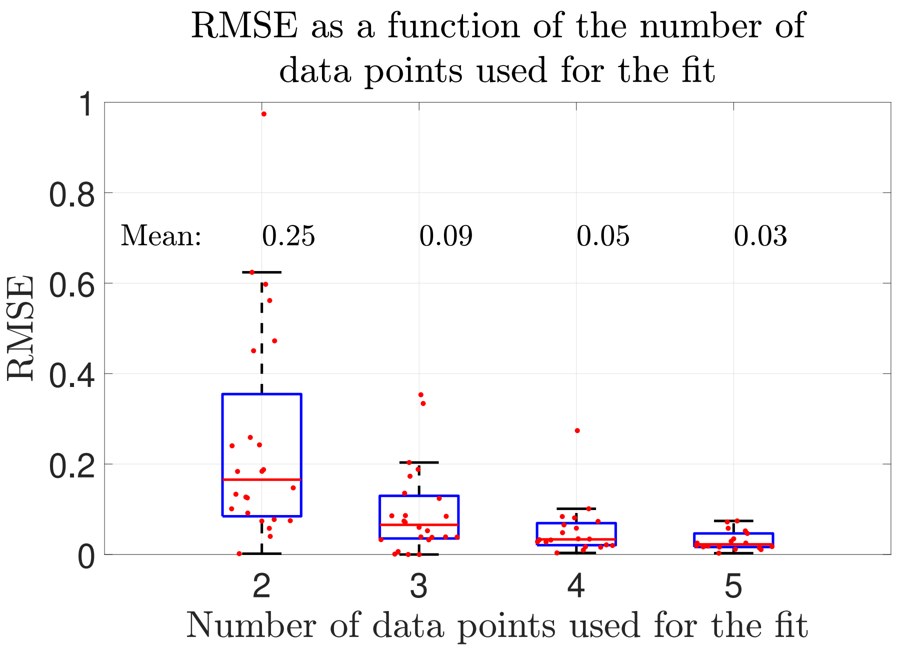


Figure S15: Boxplots of the RMSE-values for all data points as a function of the number of data points used in the fit. The red dots around the box plots are the individual RMSE-values. Patients with fewer than $m$ observations are excluded from the boxplots for $m$ data points used. Only 4 patients had more than 5 data points available, and therefore boxplots are shown only for $m \leq 5$.

Figure S15 shows boxplots of the RMSE-values for all data points as a function of the number of used data points used in the fit. The reason for using all data points for the RMSE-values is that when quantifying the model's predictive properties, the result would be overly optimistic if we were including only the data on which it is fitted. Figure S15 shows that the model, as expected, fits better to the available data, and thus has better predictive properties, the more data points are used to calculate the optimal fit. The mean RMSE for all data points is 0.25 (25%) when only 2 data points are used in the calculation of the optimal fit, but this mean decreases rapidly to 0.09 (9%), 0.05 (5%), and 0.03 (3%), when 3, 4, and 5 data points are used, respectively.

Figure S16 shows how the model predictions of the *JAK2* VAF varies for patient 1 when using different values of the fitting parameters, $\rho_{s_{y0}}$ and $\rho_{d_{y1}}$. Here, we take the optimal values of $\rho_{s_{y0}}$ and $\rho_{d_{y1}}$, scale them by the factors 0, 0.5, 1.0, and 1.5, and look at the resulting effects on the model predictions. From figure S16, we see that when $\rho_{s_{y0}}$ is lower than the optimal value from the fitting, the model has difficulties capturing the later data points of the patient. On the other hand, when $\rho_{d_{y1}}$ is lower than the optimal value from the fitting, the model has difficulties capturing the earlier data points of the patient. However, when the drug-induced impact on the parameters is either decreased or increased via the non-zero multiplication factors on $\rho_{s_{y0}}$ and/or $\rho_{d_{y1}}$, the model still produces reasonable fits. As explained in section 2.1.3 of the main text, a scaling of the fitting parameters, $\rho_{s_{y0}}$ and $\rho_{d_{y1}}$, is interchangeable with the same scaling of the dosing, $c_R(t)$. Thus, the plots in the diagonal of figure S16 also illustrate the model predictions of scaling the RUX dose for patient 1. Interestingly, the model predicts that this patient will eventually be cured even if only half of the RUX dose is administered. Numerical experiments can estimate a *critical*

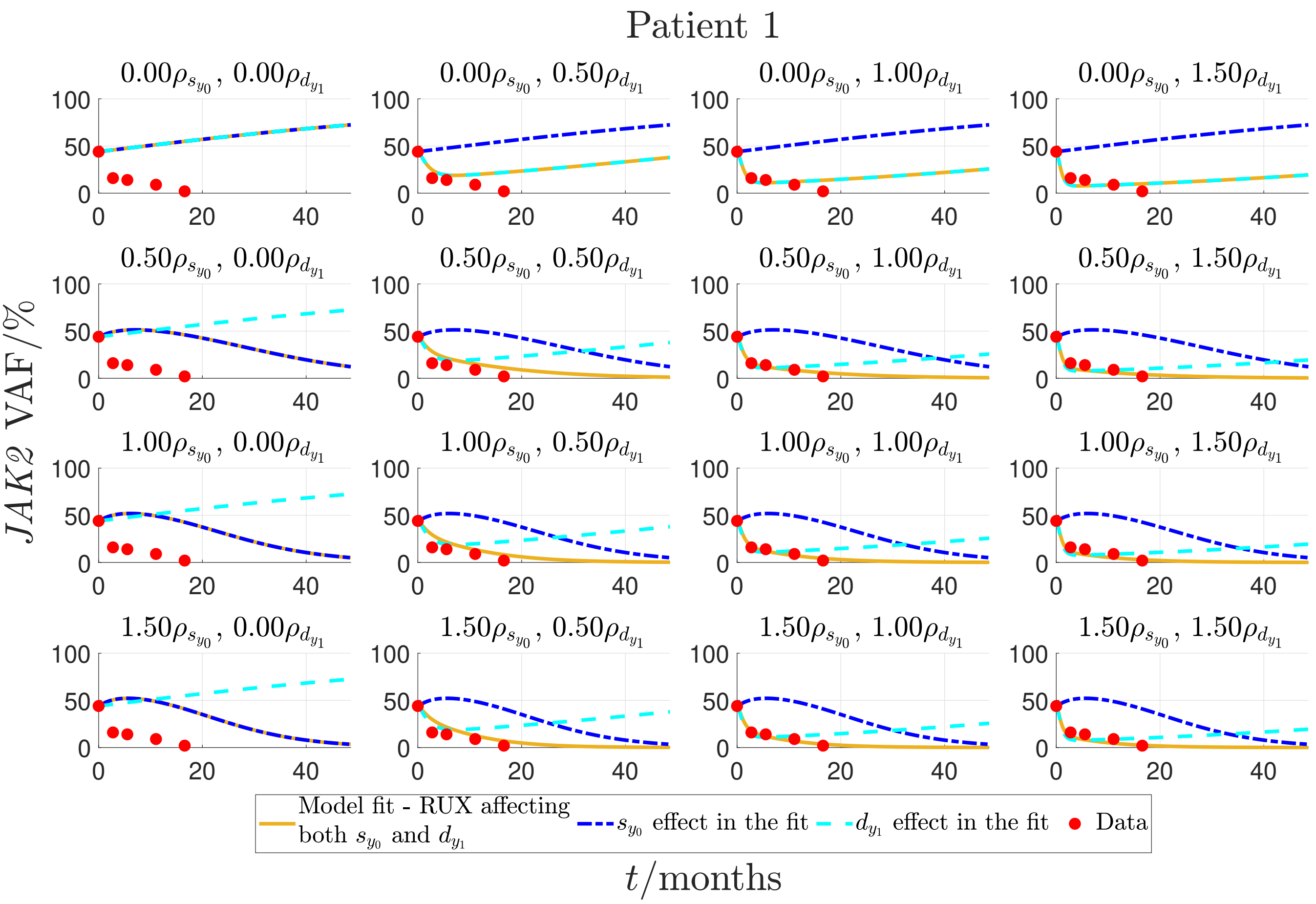


Figure S16: Model predictions for multiplying the optimal fitting parameters, $\rho_{s_{y0}}$ and $\rho_{d_{y1}}$, by the factors 0, 0.5, 1.0, and 1.5 for patient 1. The solid yellow curves are the model fitted to the data, the red dots are the data points, the dashed blue lines are the effect of varying only $s_{y0}$ (setting $\rho_{d_{y1}} = 0$) in the optimal fits, and the dashed cyan lines are the effect of varying only $d_{y1}$ (setting $\rho_{s_{y0}} = 0$) in the optimal fits.

*dose*, above which the model predicts that the patient will eventually be cured. Further, increasing the dose by a factor 1.5 (bottom right corner of the figure) seems to not provide a huge improvement in the *JAK2* VAF response, and thus the gain in *JAK2* VAF reduction when increasing the dose might not be worth the (potential) increased side effects of such a dose increase. When interpreting these results, one has to be aware of the simplifying assumptions made in the pharmacokinetic/pharmacodynamic model of RUX. The proposed model can quantify how changes of cell parameters impact the course of the disease. How changes of drug doses are quantitatively related to changes of cell parameters is a complex question and beyond the scope of this work.

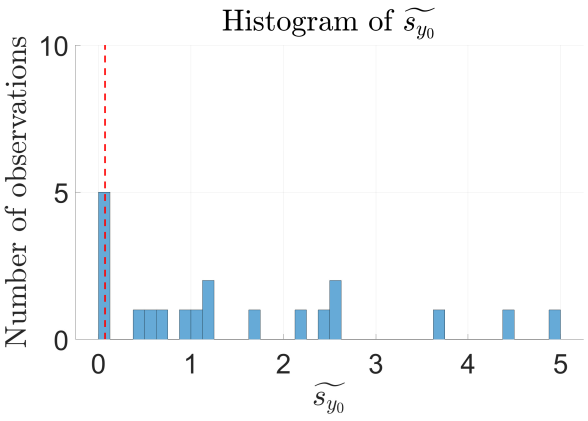


Figure 17a

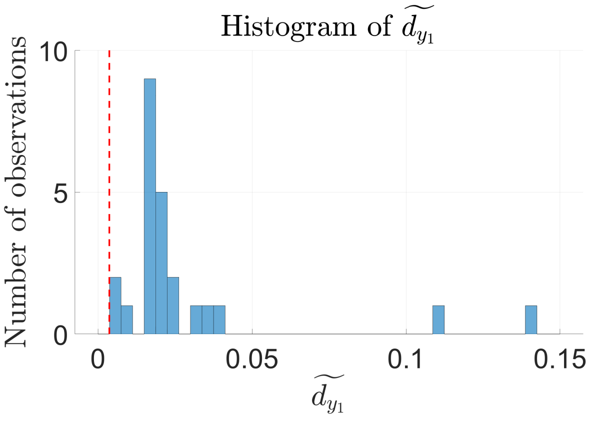


Figure 17b

Figure S17: Histogram of the parameter values $\widetilde{s_{y0}}$ and $\widetilde{d_{y1}}$ for all 24 patients during RUX treatment calculated using equation (3) from the main text. The dashed red lines show the corresponding standard parameter values before treatment from table 2 from the main text. **(a)** $\widetilde{s_{y0}}$. 4 outliers (patients 4, 22, 23 and 24) with $\widetilde{s_{y0}} > 16$ are not shown in the figure. See table 4 from the main text for more details. **(b)** $\widetilde{d_{y1}}$. There are no observations outside the shown range.

## 6 PLOTS OF INDIVIDUAL PATIENT FITS TO DATA

Here, we present the plots of the fitting of the model to the individual patient data for all 24 patients. First we present a plot of the full output of the model, and then we present the convergence of the fitting procedure when adding the data points sequentially. In the plots of the cells, the solid green curves represent the number of healthy cells, the solid red curves represent the number of malignant cells, and the dashed black curves represent the sum of healthy and malignant cells. For the plots of the *JAK2* VAF, the yellow curve is the model predictions, the filled red dots are the data points used in the fit, the empty red dots are the data points not used for the fit, the grey areas are the approximate 95% CI for the model predictions calculated using the sampling technique as explained in section S4, the dotted blue line is the model prediction when varying only $s_{y_0}$ in the optimal fit (i.e. setting $\rho_{d_{y_1}} = 0$), and the dotted cyan curve is the model prediction when varying only $d_{y_1}$ in the optimal fit (i.e. setting $\rho_{s_{y_0}} = 0$). For the sequential fits, the individual sequential fits are optimised with respect to the non-linear least-squares objective function for only the points used in the data fitting procedure (the filled red dots), but the reported RMSE-value is calculated using all data points (the filled red dots and the empty red dots). This is to show how the fits of the model improve when adding more data and to illustrate the model's predictive properties. If we looked only at the RMSE-values with respect to only the data points used in the fit, the model performance would be overly optimistic.

## 6.1 Patient 1

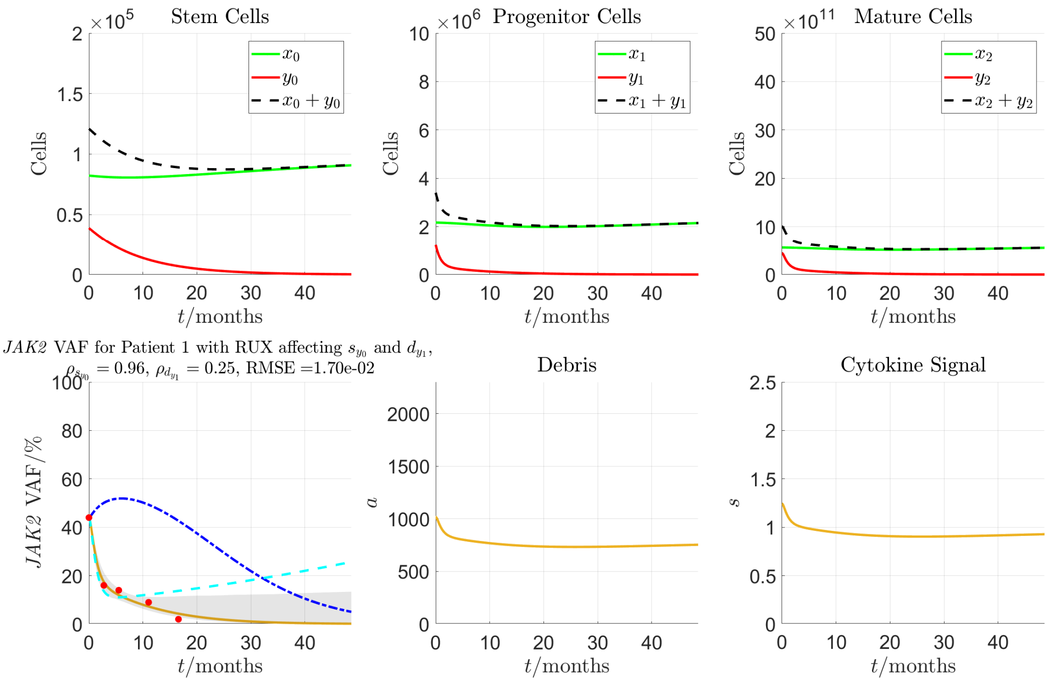

Stem Cells
Progenitor Cells
Mature Cells
Cells
$\times 10^5$
$\times 10^6$
$\times 10^{11}$
$x_0$
$y_0$
$x_0 + y_0$
$x_1$
$y_1$
$x_1 + y_1$
$x_2$
$y_2$
$x_2 + y_2$
$t$/months
*JAK2* VAF for Patient 1 with RUX affecting $s_{y_0}$ and $d_{y_1}$,
$\rho_{s_{y_0}} = 0.96$, $\rho_{d_{y_1}} = 0.25$, RMSE =1.70e-02
*JAK2* VAF/%
Debris
$a$
Cytokine Signal
$s$


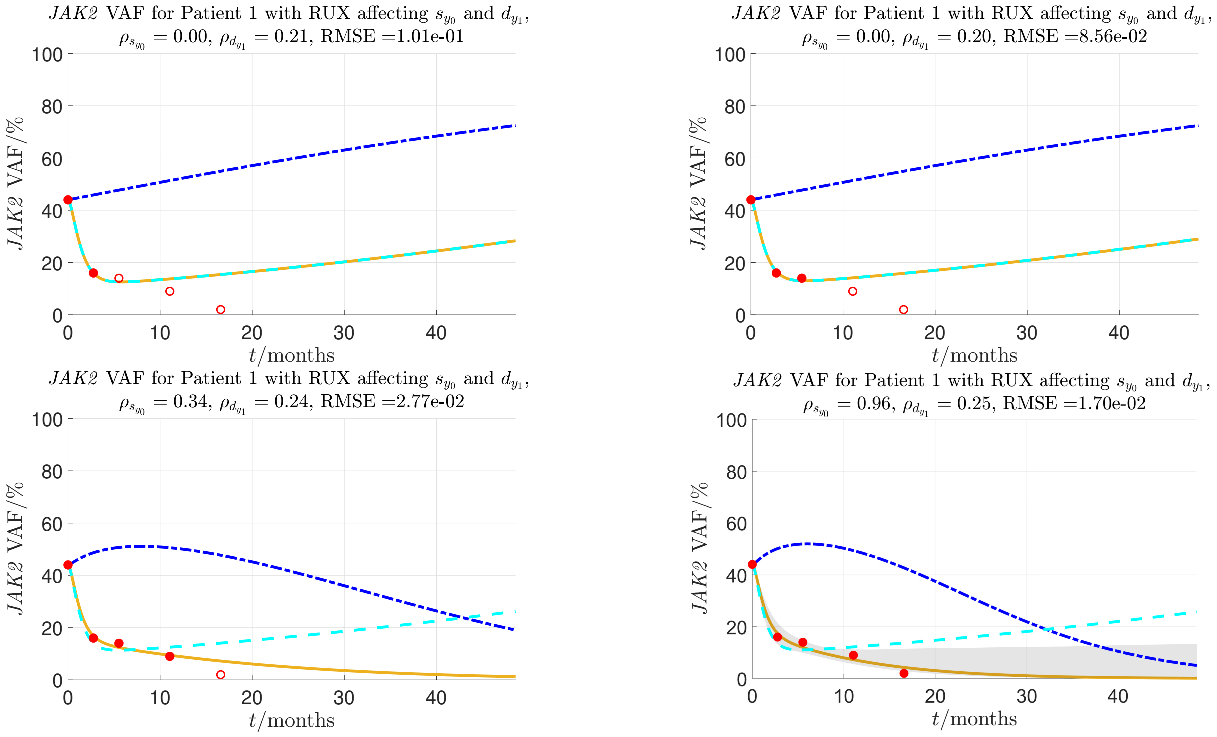

*JAK2* VAF for Patient 1 with RUX affecting $s_{y_0}$ and $d_{y_1}$,
$\rho_{s_{y_0}} = 0.00$, $\rho_{d_{y_1}} = 0.21$, RMSE =1.01e-01
*JAK2* VAF for Patient 1 with RUX affecting $s_{y_0}$ and $d_{y_1}$,
$\rho_{s_{y_0}} = 0.00$, $\rho_{d_{y_1}} = 0.20$, RMSE =8.56e-02
*JAK2* VAF for Patient 1 with RUX affecting $s_{y_0}$ and $d_{y_1}$,
$\rho_{s_{y_0}} = 0.34$, $\rho_{d_{y_1}} = 0.24$, RMSE =2.77e-02
*JAK2* VAF for Patient 1 with RUX affecting $s_{y_0}$ and $d_{y_1}$,
$\rho_{s_{y_0}} = 0.96$, $\rho_{d_{y_1}} = 0.25$, RMSE =1.70e-02
*JAK2* VAF/%
$t$/months

## 6.2 Patient 2

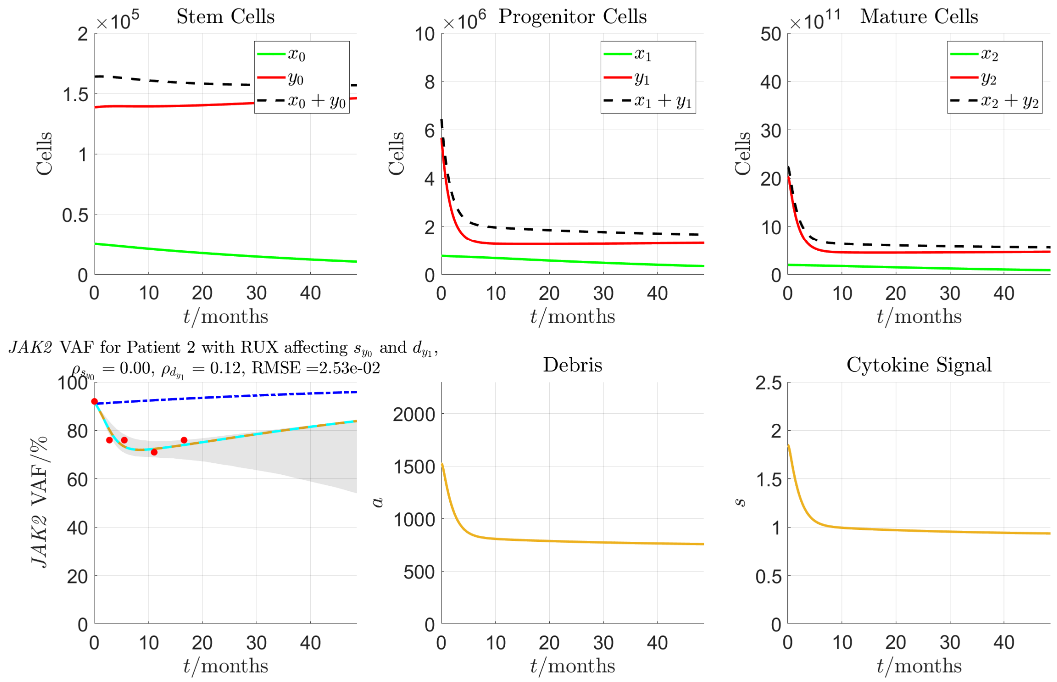
Stem Cells
$\times 10^5$
$x_0$
$y_0$
$x_0 + y_0$
Cells
$t$/months
Progenitor Cells
$\times 10^6$
$x_1$
$y_1$
$x_1 + y_1$
Cells
$t$/months
Mature Cells
$\times 10^{11}$
$x_2$
$y_2$
$x_2 + y_2$
Cells
$t$/months
*JAK2* VAF for Patient 2 with RUX affecting $s_{y_0}$ and $d_{y_1}$,
$\rho_{s_{y_0}} = 0.00$, $\rho_{d_{y_1}} = 0.12$, RMSE =2.53e-02
*JAK2* VAF/%
$t$/months
Debris
$a$
$t$/months
Cytokine Signal
$s$
$t$/months

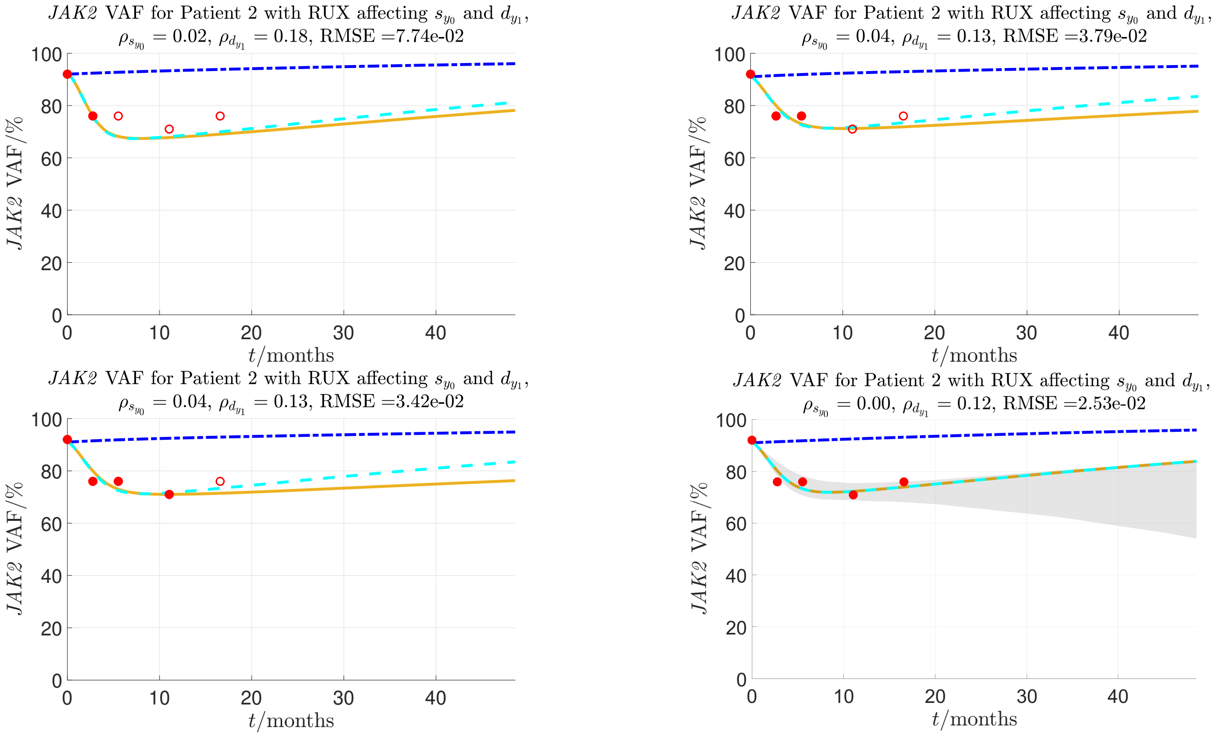
*JAK2* VAF for Patient 2 with RUX affecting $s_{y_0}$ and $d_{y_1}$,
$\rho_{s_{y_0}} = 0.02$, $\rho_{d_{y_1}} = 0.18$, RMSE =7.74e-02
*JAK2* VAF/%
$t$/months
*JAK2* VAF for Patient 2 with RUX affecting $s_{y_0}$ and $d_{y_1}$,
$\rho_{s_{y_0}} = 0.04$, $\rho_{d_{y_1}} = 0.13$, RMSE =3.79e-02
*JAK2* VAF/%
$t$/months
*JAK2* VAF for Patient 2 with RUX affecting $s_{y_0}$ and $d_{y_1}$,
$\rho_{s_{y_0}} = 0.04$, $\rho_{d_{y_1}} = 0.13$, RMSE =3.42e-02
*JAK2* VAF/%
$t$/months
*JAK2* VAF for Patient 2 with RUX affecting $s_{y_0}$ and $d_{y_1}$,
$\rho_{s_{y_0}} = 0.00$, $\rho_{d_{y_1}} = 0.12$, RMSE =2.53e-02
*JAK2* VAF/%
$t$/months

## 6.3 Patient 3

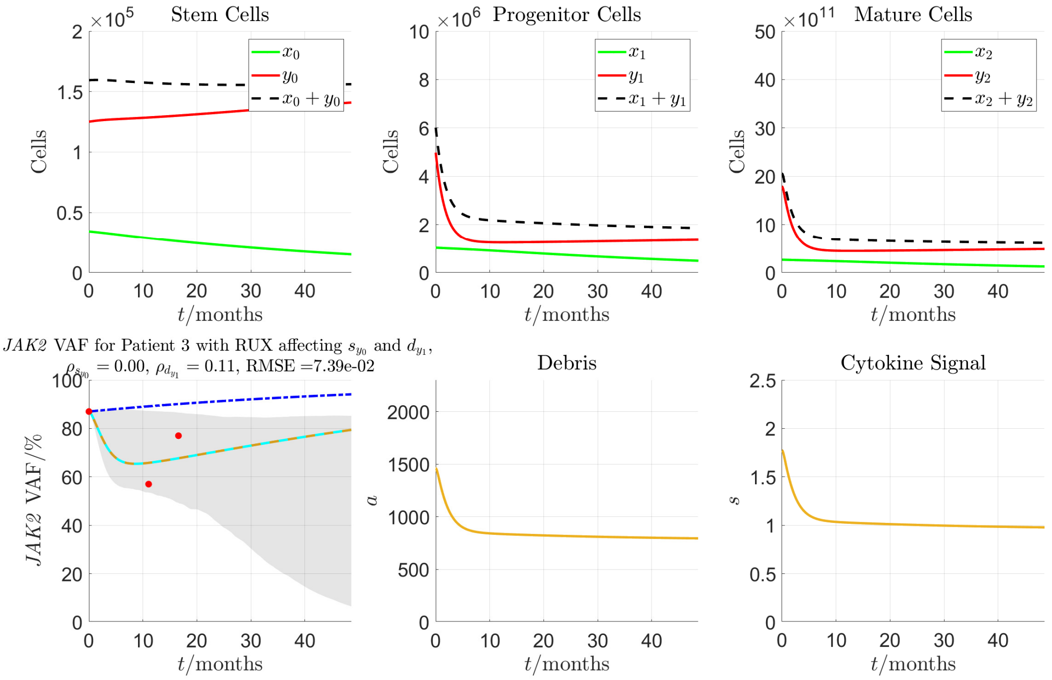

Stem Cells
Progenitor Cells
Mature Cells
Cells
$t$/months
$x_0$
$y_0$
$x_0 + y_0$
$x_1$
$y_1$
$x_1 + y_1$
$x_2$
$y_2$
$x_2 + y_2$
$\times 10^5$
$\times 10^6$
$\times 10^{11}$
*JAK2* VAF for Patient 3 with RUX affecting $s_{y_0}$ and $d_{y_1}$, $\rho_{s_{y_0}} = 0.00$, $\rho_{d_{y_1}} = 0.11$, RMSE =7.39e-02
*JAK2* VAF/%
Debris
$a$
Cytokine Signal
$s$


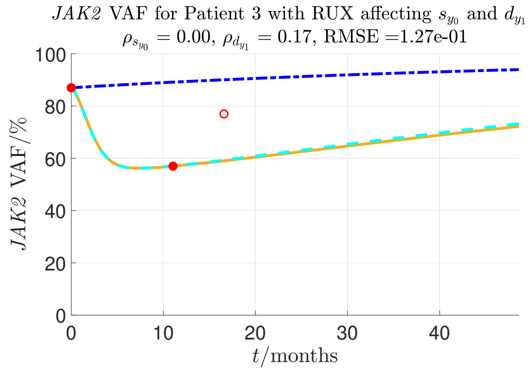

*JAK2* VAF for Patient 3 with RUX affecting $s_{y_0}$ and $d_{y_1}$, $\rho_{s_{y_0}} = 0.00$, $\rho_{d_{y_1}} = 0.17$, RMSE =1.27e-01
*JAK2* VAF/%
$t$/months


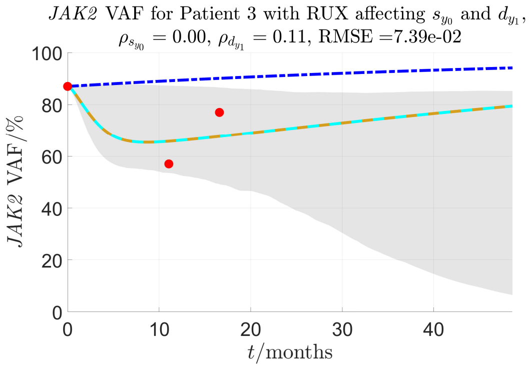

*JAK2* VAF for Patient 3 with RUX affecting $s_{y_0}$ and $d_{y_1}$, $\rho_{s_{y_0}} = 0.00$, $\rho_{d_{y_1}} = 0.11$, RMSE =7.39e-02
*JAK2* VAF/%
$t$/months

## 6.4 Patient 4

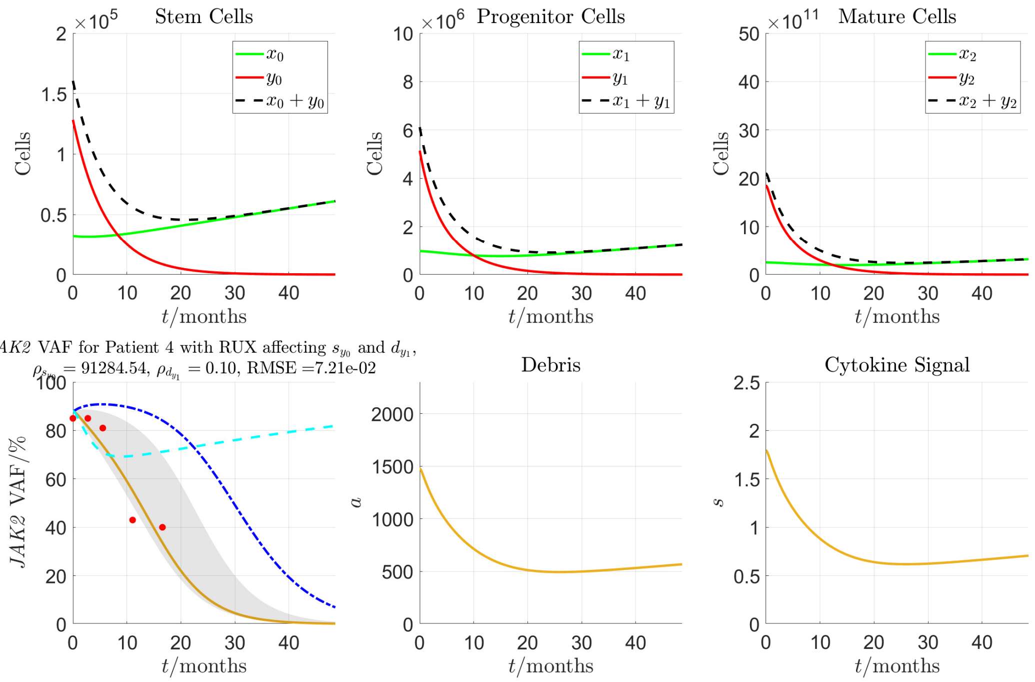
Stem Cells
Progenitor Cells
Mature Cells
$\times 10^5$
$\times 10^6$
$\times 10^{11}$
Cells
$x_0$
$y_0$
$x_0 + y_0$
$x_1$
$y_1$
$x_1 + y_1$
$x_2$
$y_2$
$x_2 + y_2$
$t$/months
*JAK2* VAF for Patient 4 with RUX affecting $s_{y_0}$ and $d_{y_1}$,
$\rho_{s_{y_0}}$ = 91284.54, $\rho_{d_{y_1}}$ = 0.10, RMSE =7.21e-02
*JAK2* VAF/%
Debris
$a$
Cytokine Signal
$s$

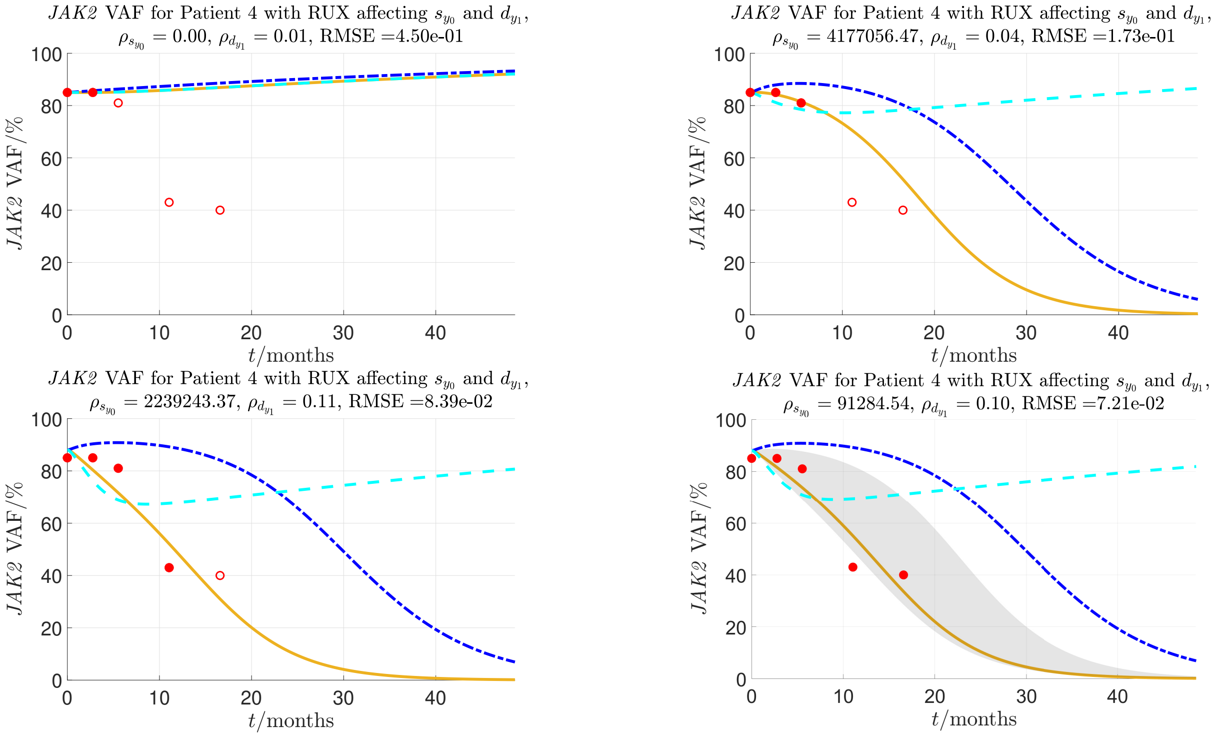
*JAK2* VAF for Patient 4 with RUX affecting $s_{y_0}$ and $d_{y_1}$,
$\rho_{s_{y_0}}$ = 0.00, $\rho_{d_{y_1}}$ = 0.01, RMSE =4.50e-01
*JAK2* VAF for Patient 4 with RUX affecting $s_{y_0}$ and $d_{y_1}$,
$\rho_{s_{y_0}}$ = 4177056.47, $\rho_{d_{y_1}}$ = 0.04, RMSE =1.73e-01
*JAK2* VAF for Patient 4 with RUX affecting $s_{y_0}$ and $d_{y_1}$,
$\rho_{s_{y_0}}$ = 2239243.37, $\rho_{d_{y_1}}$ = 0.11, RMSE =8.39e-02
*JAK2* VAF for Patient 4 with RUX affecting $s_{y_0}$ and $d_{y_1}$,
$\rho_{s_{y_0}}$ = 91284.54, $\rho_{d_{y_1}}$ = 0.10, RMSE =7.21e-02
*JAK2* VAF/%
$t$/months

## 6.5 Patient 5

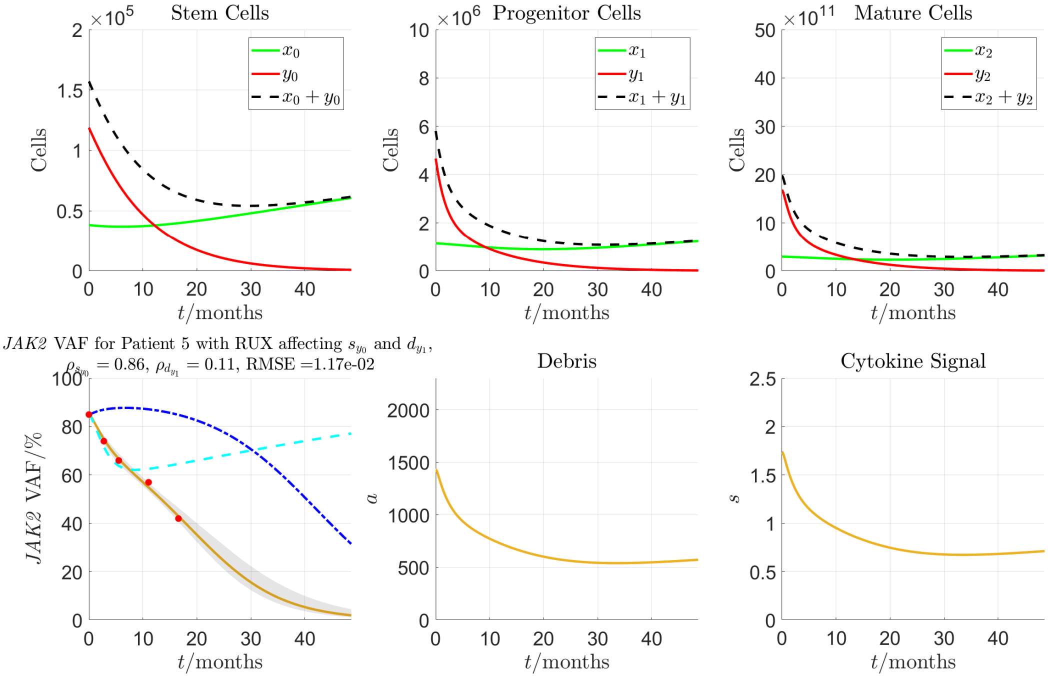
Stem Cells
Progenitor Cells
Mature Cells
Cells
$\times 10^5$
$\times 10^6$
$\times 10^{11}$
$x_0$
$y_0$
$x_0 + y_0$
$x_1$
$y_1$
$x_1 + y_1$
$x_2$
$y_2$
$x_2 + y_2$
$t$/months
*JAK2* VAF for Patient 5 with RUX affecting $s_{y_0}$ and $d_{y_1}$,
$\rho_{s_{y_0}} = 0.86$, $\rho_{d_{y_1}} = 0.11$, RMSE =1.17e-02
*JAK2* VAF/%
Debris
$a$
Cytokine Signal
$s$

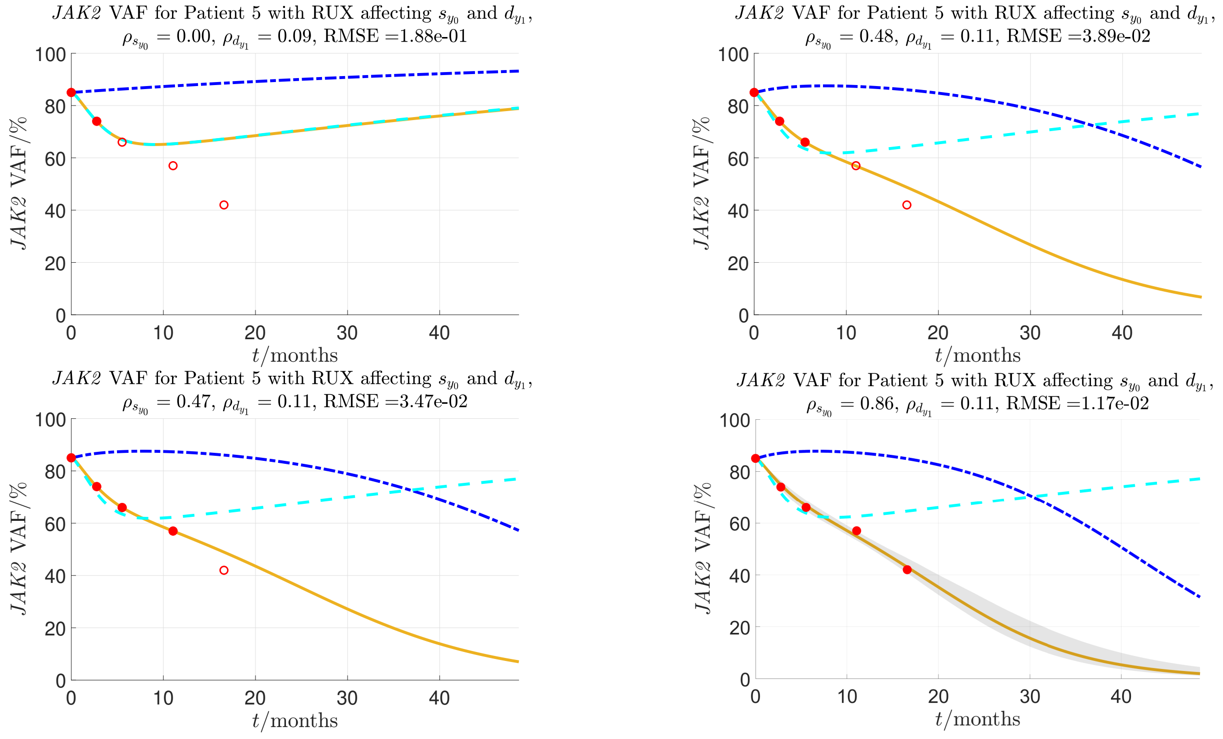
*JAK2* VAF for Patient 5 with RUX affecting $s_{y_0}$ and $d_{y_1}$,
$\rho_{s_{y_0}} = 0.00$, $\rho_{d_{y_1}} = 0.09$, RMSE =1.88e-01
*JAK2* VAF for Patient 5 with RUX affecting $s_{y_0}$ and $d_{y_1}$,
$\rho_{s_{y_0}} = 0.48$, $\rho_{d_{y_1}} = 0.11$, RMSE =3.89e-02
*JAK2* VAF for Patient 5 with RUX affecting $s_{y_0}$ and $d_{y_1}$,
$\rho_{s_{y_0}} = 0.47$, $\rho_{d_{y_1}} = 0.11$, RMSE =3.47e-02
*JAK2* VAF for Patient 5 with RUX affecting $s_{y_0}$ and $d_{y_1}$,
$\rho_{s_{y_0}} = 0.86$, $\rho_{d_{y_1}} = 0.11$, RMSE =1.17e-02
*JAK2* VAF/%
$t$/months

## 6.6 Patient 6

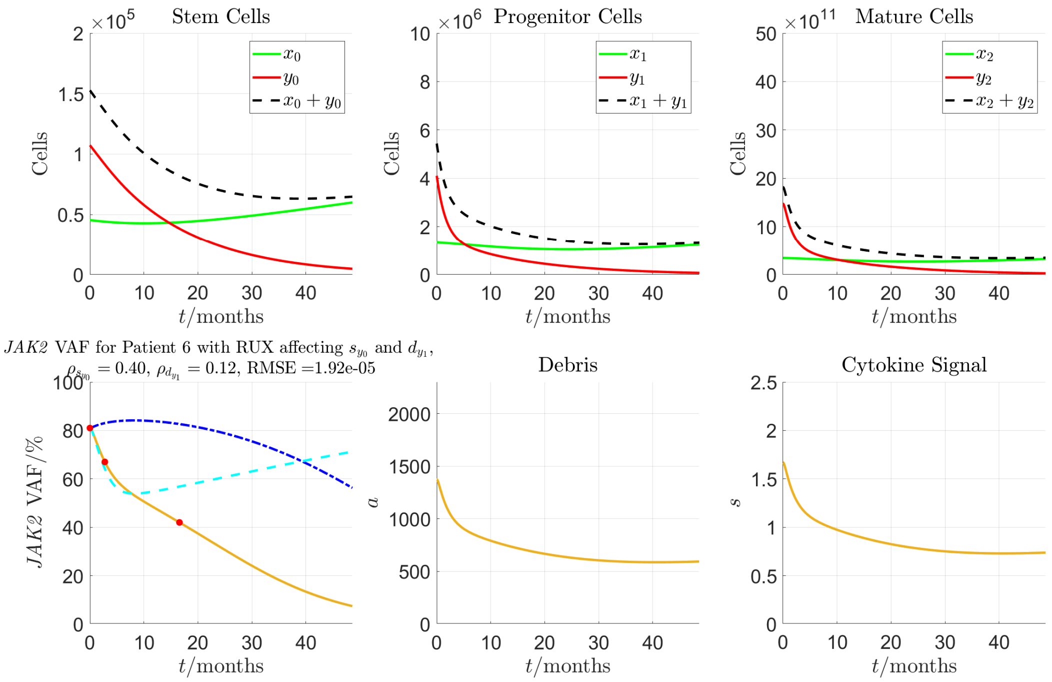
Stem Cells
Progenitor Cells
Mature Cells
Cells
$t$/months
$x_0$
$y_0$
$x_0 + y_0$
$x_1$
$y_1$
$x_1 + y_1$
$x_2$
$y_2$
$x_2 + y_2$
*JAK2* VAF for Patient 6 with RUX affecting $s_{y_0}$ and $d_{y_1}$,
$\rho_{s_{y_0}} = 0.40$, $\rho_{d_{y_1}} = 0.12$, RMSE =1.92e-05
*JAK2* VAF/%
Debris
$a$
Cytokine Signal
$s$

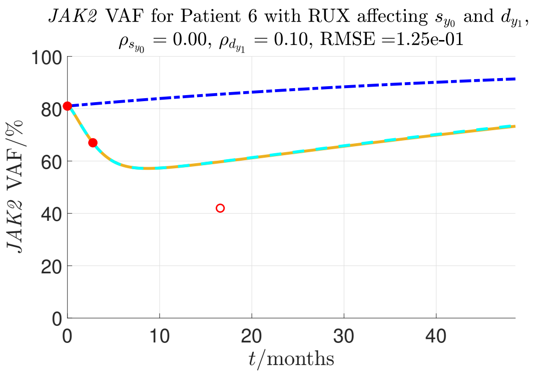
*JAK2* VAF for Patient 6 with RUX affecting $s_{y_0}$ and $d_{y_1}$,
$\rho_{s_{y_0}} = 0.00$, $\rho_{d_{y_1}} = 0.10$, RMSE =1.25e-01
*JAK2* VAF/%
$t$/months

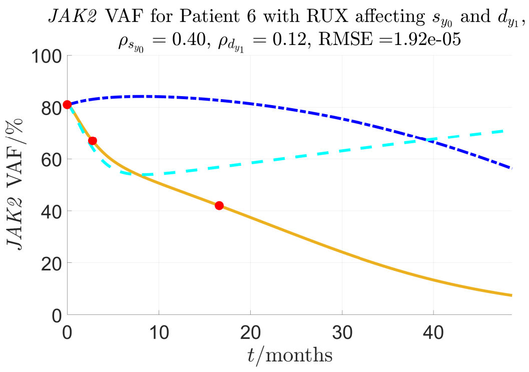
*JAK2* VAF for Patient 6 with RUX affecting $s_{y_0}$ and $d_{y_1}$,
$\rho_{s_{y_0}} = 0.40$, $\rho_{d_{y_1}} = 0.12$, RMSE =1.92e-05
*JAK2* VAF/%
$t$/months

## 6.7 Patient 7

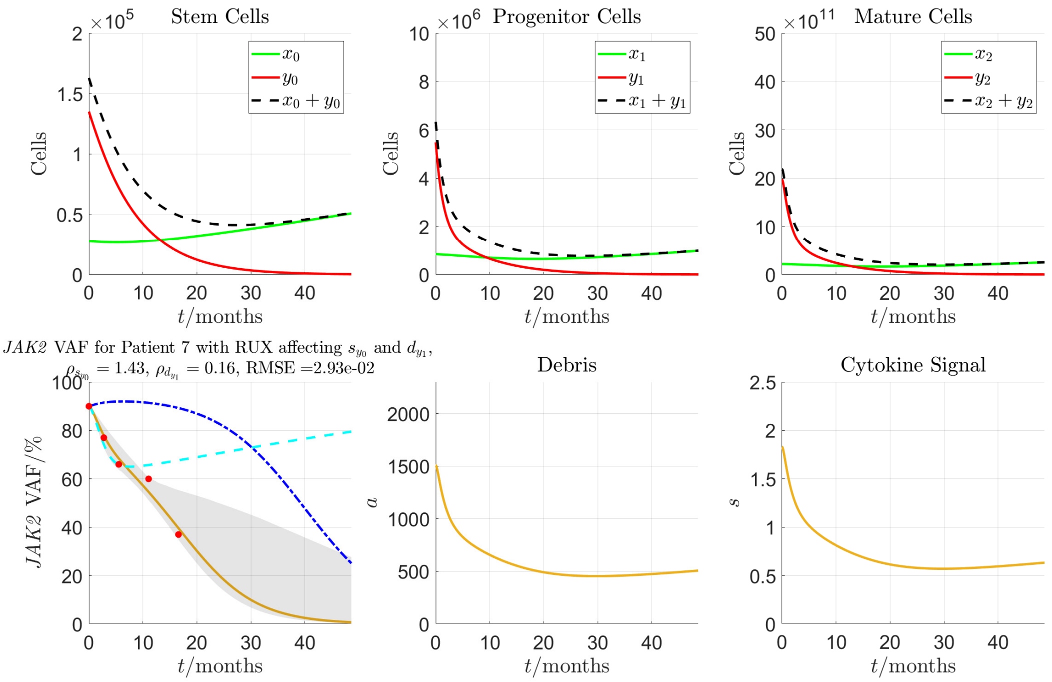
Stem Cells
Progenitor Cells
Mature Cells
Cells
$x_0$
$y_0$
$x_0 + y_0$
$x_1$
$y_1$
$x_1 + y_1$
$x_2$
$y_2$
$x_2 + y_2$
$t$/months
*JAK2* VAF for Patient 7 with RUX affecting $s_{y_0}$ and $d_{y_1}$,
$\rho_{s_{y_0}}$ = 1.43, $\rho_{d_{y_1}}$ = 0.16, RMSE =2.93e-02
*JAK2* VAF/%
Debris
$a$
Cytokine Signal
$s$

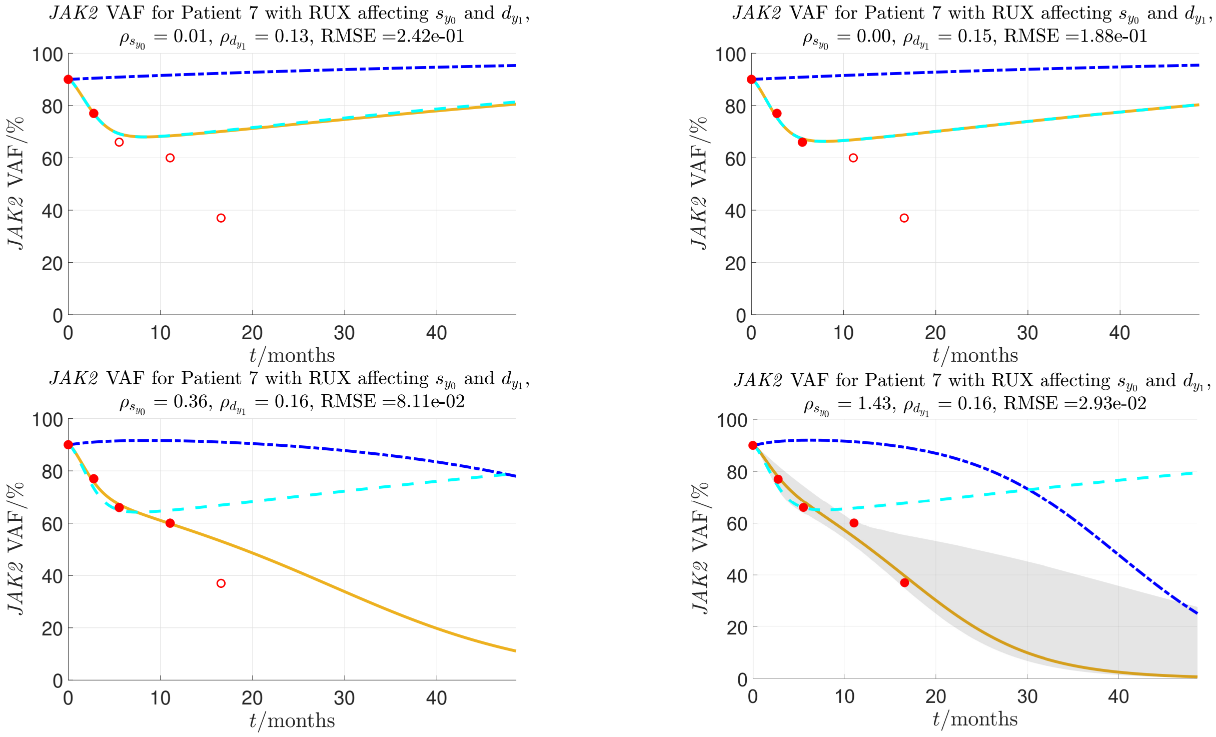
*JAK2* VAF for Patient 7 with RUX affecting $s_{y_0}$ and $d_{y_1}$,
$\rho_{s_{y_0}}$ = 0.01, $\rho_{d_{y_1}}$ = 0.13, RMSE =2.42e-01
*JAK2* VAF for Patient 7 with RUX affecting $s_{y_0}$ and $d_{y_1}$,
$\rho_{s_{y_0}}$ = 0.00, $\rho_{d_{y_1}}$ = 0.15, RMSE =1.88e-01
*JAK2* VAF for Patient 7 with RUX affecting $s_{y_0}$ and $d_{y_1}$,
$\rho_{s_{y_0}}$ = 0.36, $\rho_{d_{y_1}}$ = 0.16, RMSE =8.11e-02
*JAK2* VAF for Patient 7 with RUX affecting $s_{y_0}$ and $d_{y_1}$,
$\rho_{s_{y_0}}$ = 1.43, $\rho_{d_{y_1}}$ = 0.16, RMSE =2.93e-02
*JAK2* VAF/%
$t$/months

## 6.8 Patient 8

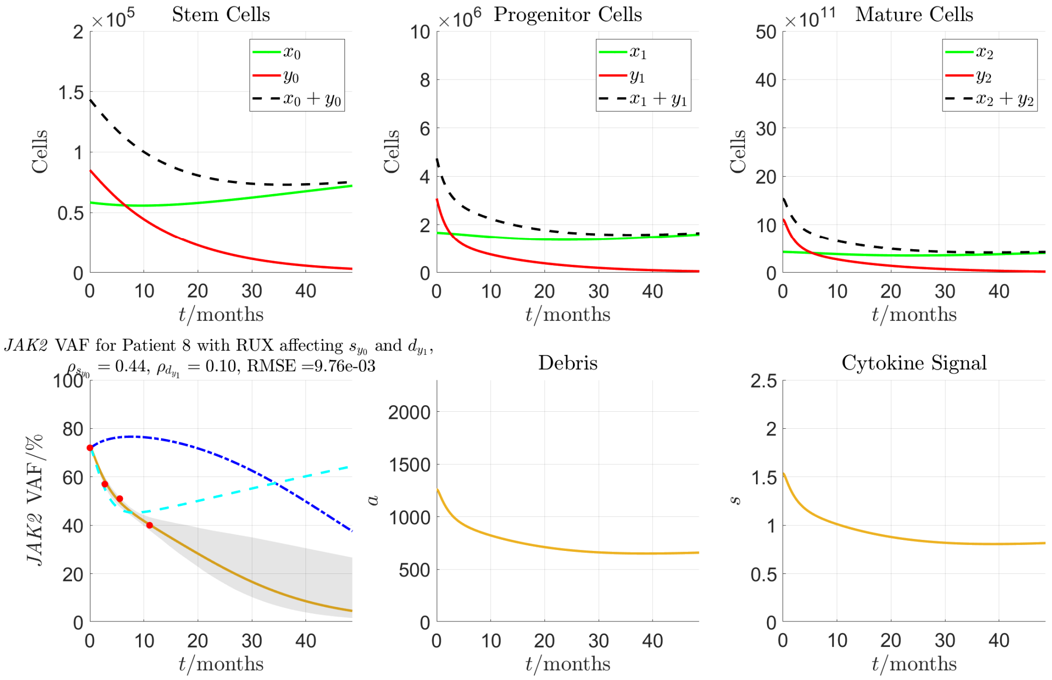
Stem Cells
Progenitor Cells
Mature Cells
Cells
$t$/months
$x_0$
$y_0$
$x_0 + y_0$
$x_1$
$y_1$
$x_1 + y_1$
$x_2$
$y_2$
$x_2 + y_2$
*JAK2* VAF for Patient 8 with RUX affecting $s_{y_0}$ and $d_{y_1}$,
$\rho_{s_{y_0}} = 0.44$, $\rho_{d_{y_1}} = 0.10$, RMSE =9.76e-03
*JAK2* VAF/%
Debris
$a$
Cytokine Signal
$s$

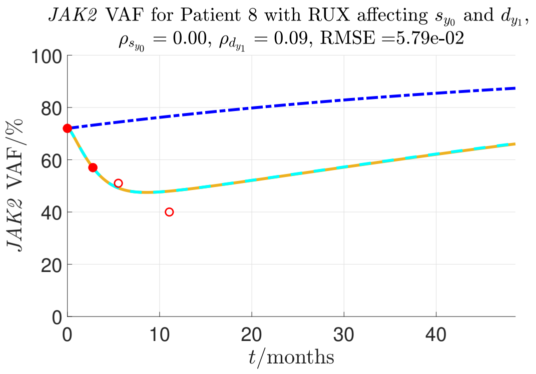
*JAK2* VAF for Patient 8 with RUX affecting $s_{y_0}$ and $d_{y_1}$,
$\rho_{s_{y_0}} = 0.00$, $\rho_{d_{y_1}} = 0.09$, RMSE =5.79e-02
*JAK2* VAF/%
$t$/months

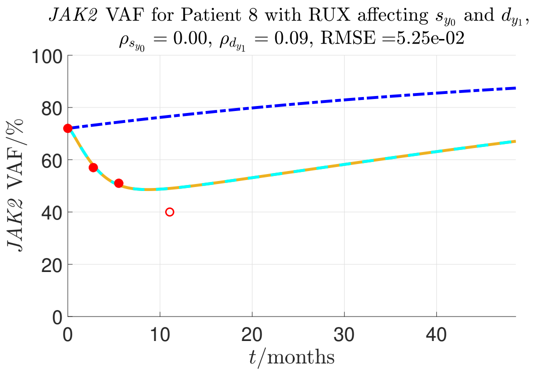
*JAK2* VAF for Patient 8 with RUX affecting $s_{y_0}$ and $d_{y_1}$,
$\rho_{s_{y_0}} = 0.00$, $\rho_{d_{y_1}} = 0.09$, RMSE =5.25e-02
*JAK2* VAF/%
$t$/months

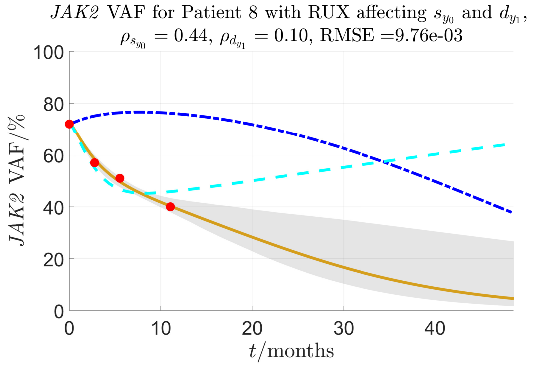
*JAK2* VAF for Patient 8 with RUX affecting $s_{y_0}$ and $d_{y_1}$,
$\rho_{s_{y_0}} = 0.44$, $\rho_{d_{y_1}} = 0.10$, RMSE =9.76e-03
*JAK2* VAF/%
$t$/months

## 6.9 Patient 9

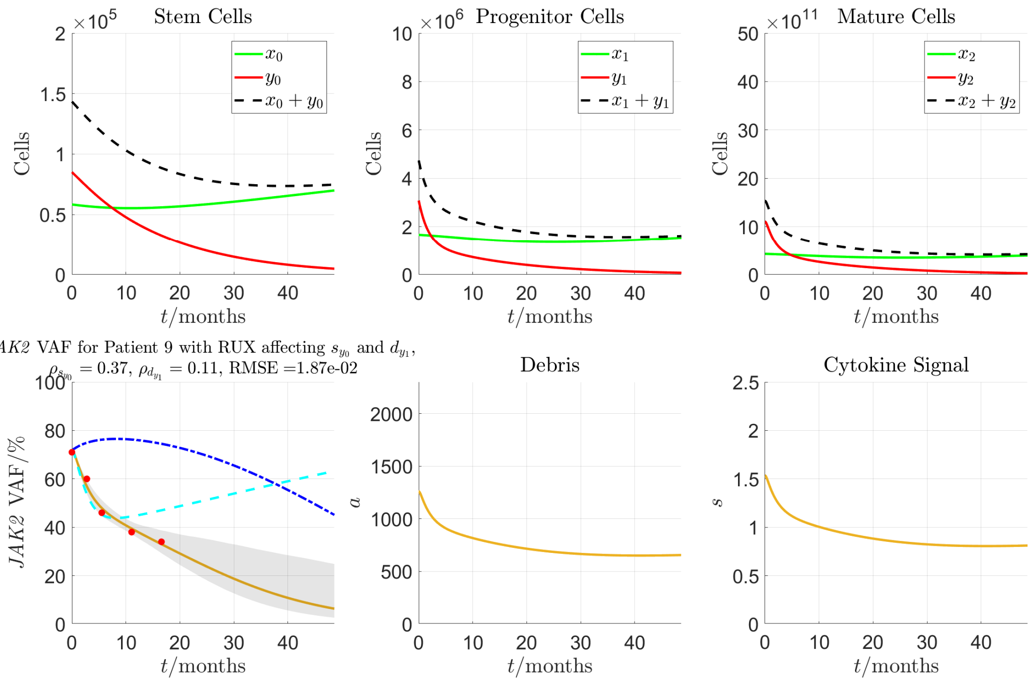

Stem Cells
Progenitor Cells
Mature Cells
Cells
$\times 10^5$
$\times 10^6$
$\times 10^{11}$
$x_0$
$y_0$
$x_0 + y_0$
$x_1$
$y_1$
$x_1 + y_1$
$x_2$
$y_2$
$x_2 + y_2$
$t$/months
*JAK2* VAF for Patient 9 with RUX affecting $s_{y_0}$ and $d_{y_1}$,
$\rho_{s_{y_0}} = 0.37$, $\rho_{d_{y_1}} = 0.11$, RMSE =1.87e-02
*JAK2* VAF/%
Debris
$a$
Cytokine Signal
$s$


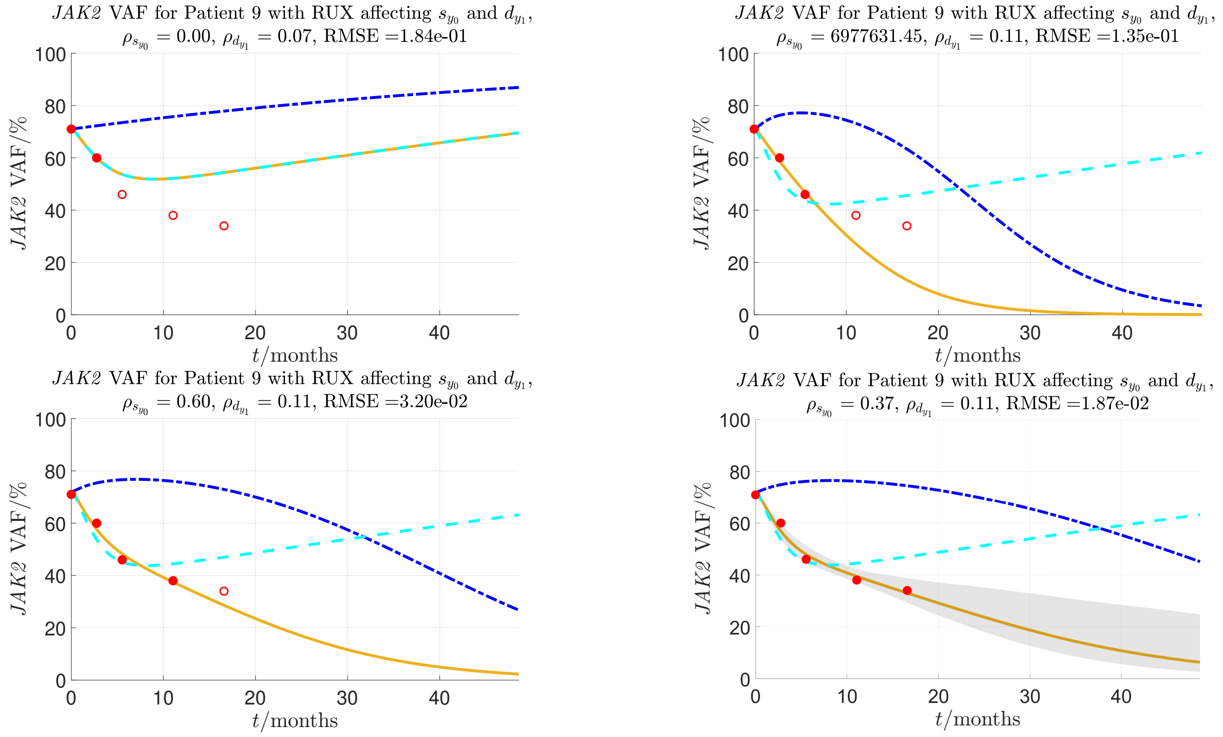

*JAK2* VAF for Patient 9 with RUX affecting $s_{y_0}$ and $d_{y_1}$,
$\rho_{s_{y_0}} = 0.00$, $\rho_{d_{y_1}} = 0.07$, RMSE =1.84e-01
*JAK2* VAF for Patient 9 with RUX affecting $s_{y_0}$ and $d_{y_1}$,
$\rho_{s_{y_0}} = 6977631.45$, $\rho_{d_{y_1}} = 0.11$, RMSE =1.35e-01
*JAK2* VAF for Patient 9 with RUX affecting $s_{y_0}$ and $d_{y_1}$,
$\rho_{s_{y_0}} = 0.60$, $\rho_{d_{y_1}} = 0.11$, RMSE =3.20e-02
*JAK2* VAF for Patient 9 with RUX affecting $s_{y_0}$ and $d_{y_1}$,
$\rho_{s_{y_0}} = 0.37$, $\rho_{d_{y_1}} = 0.11$, RMSE =1.87e-02
*JAK2* VAF/%
$t$/months

## 6.10 Patient 10

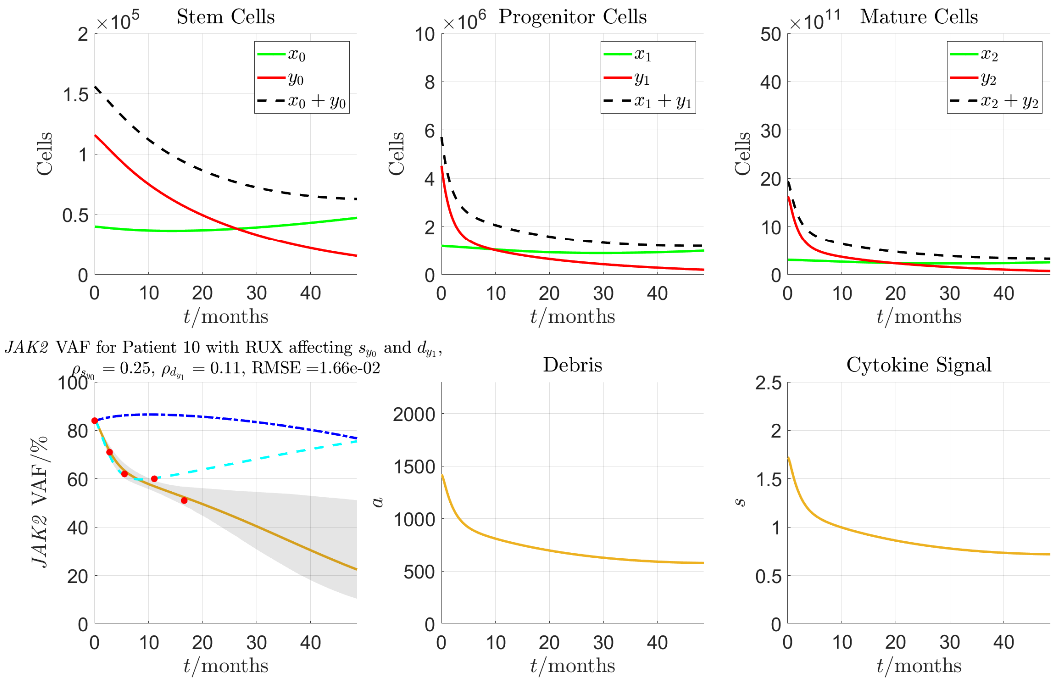


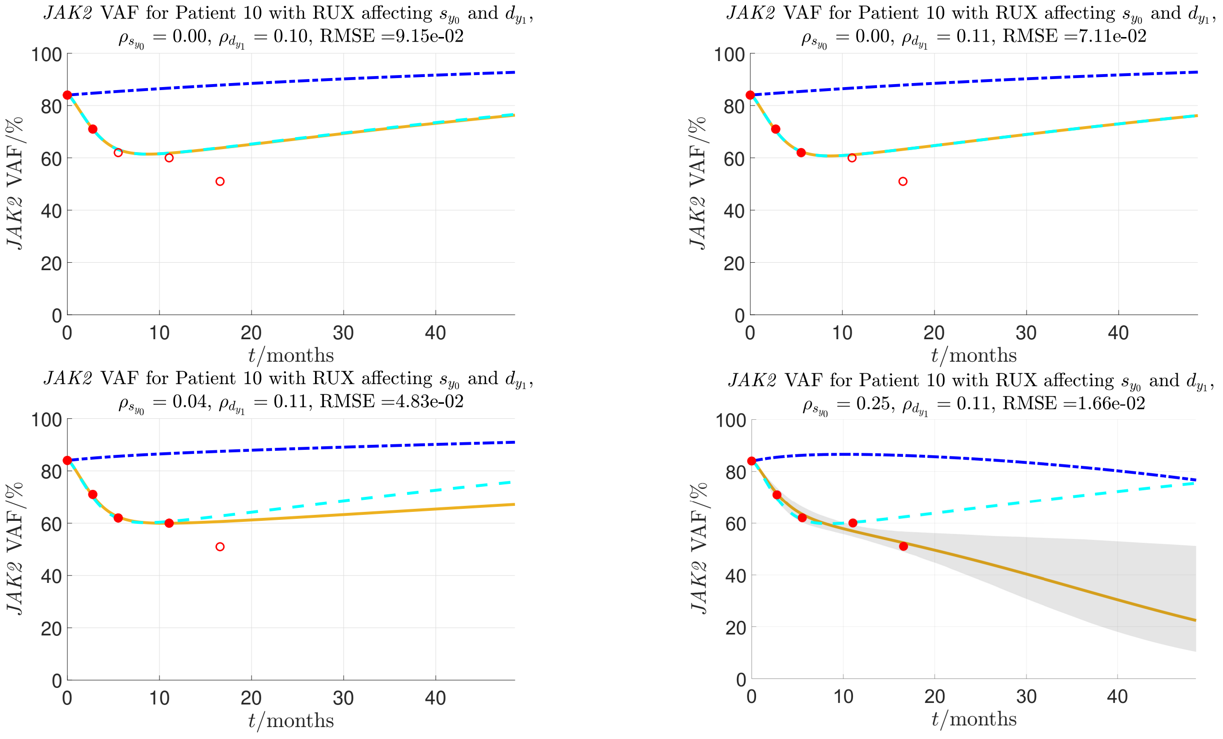

## 6.11 Patient 11

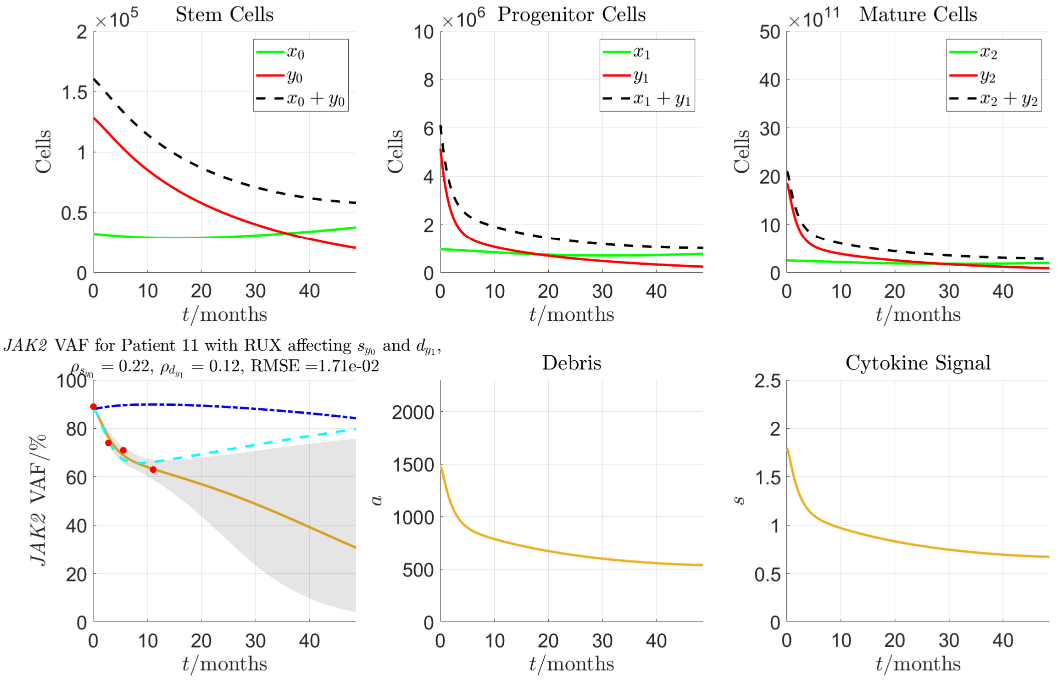


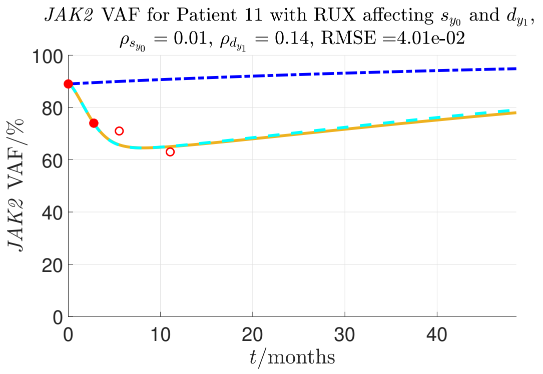


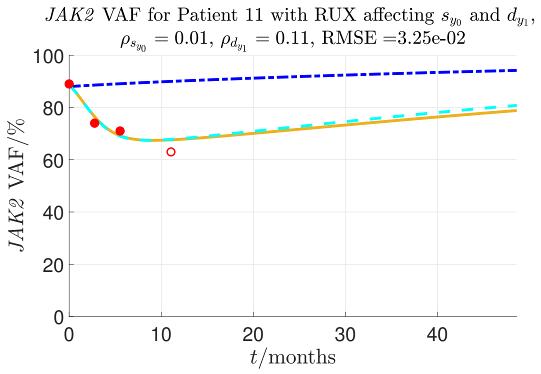


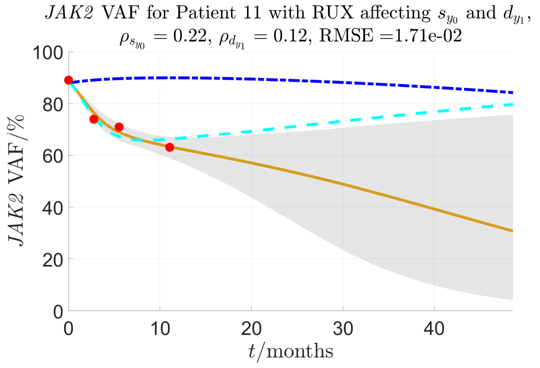

## 6.12 Patient 12

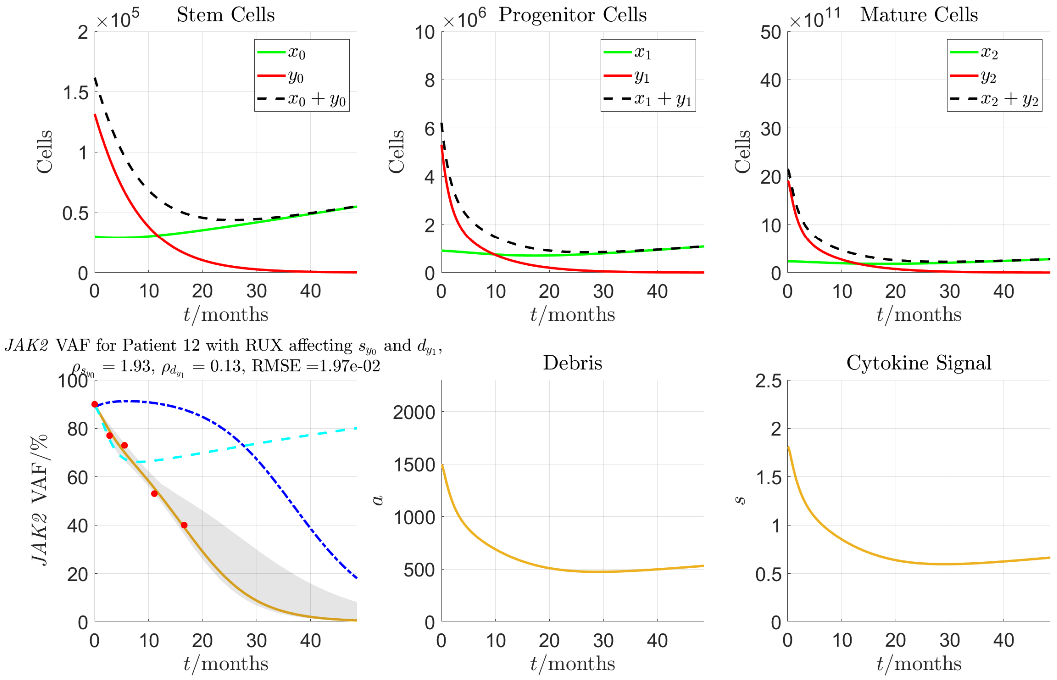


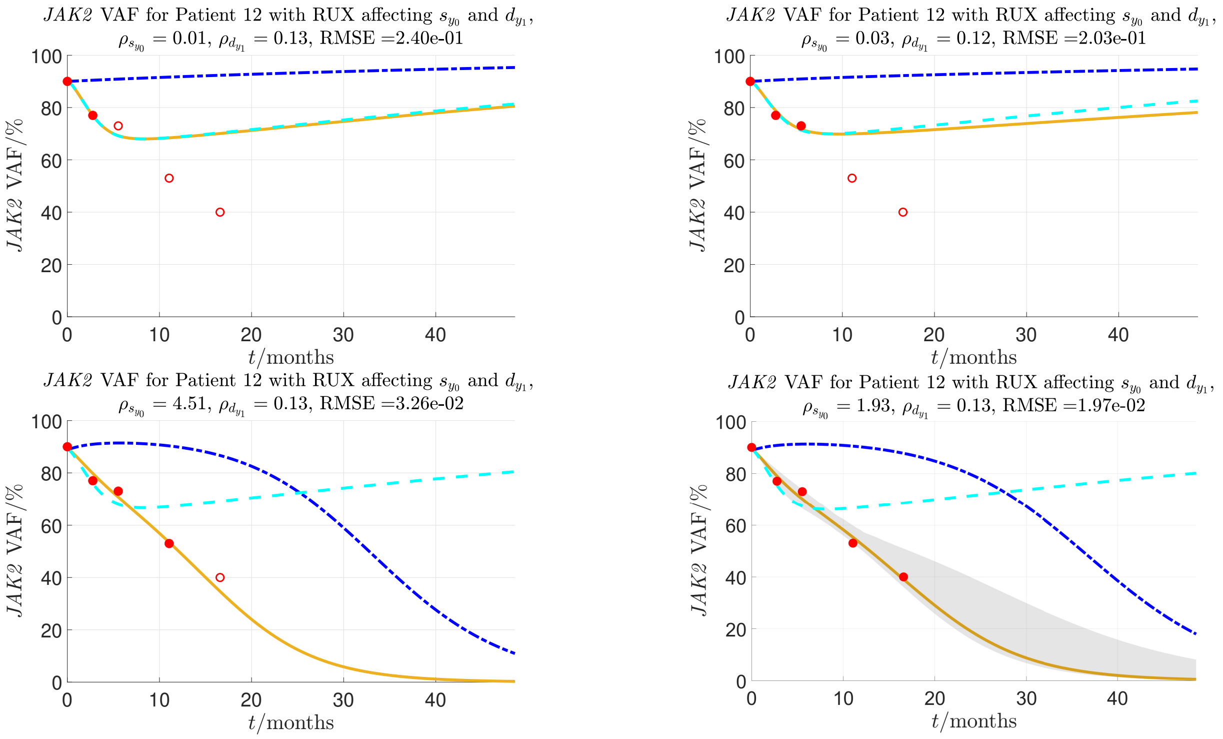

## 6.13 Patient 13

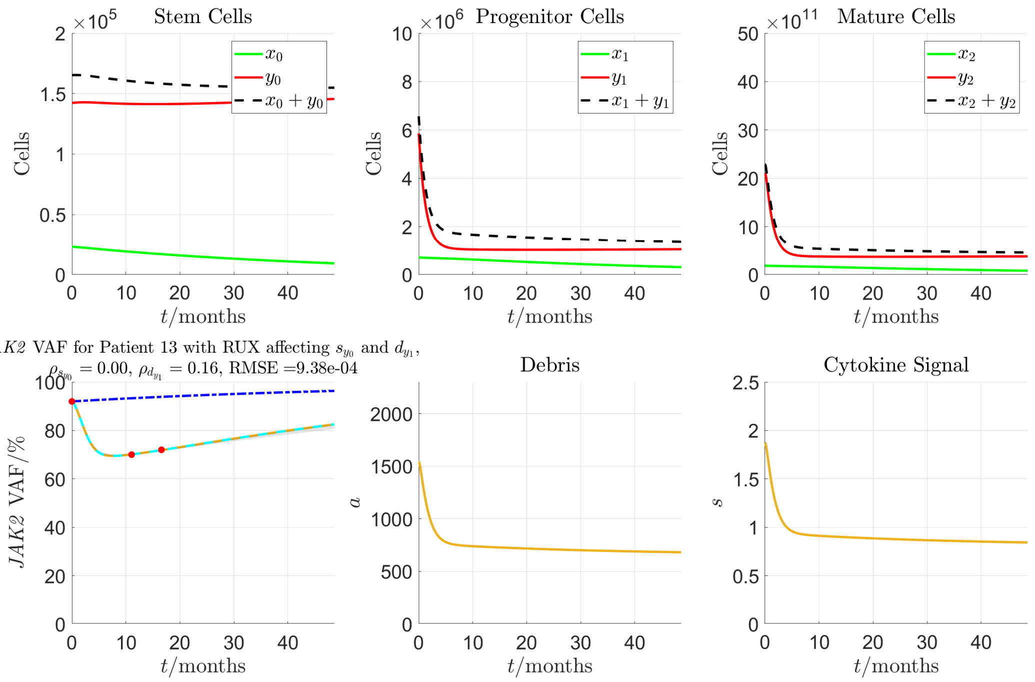


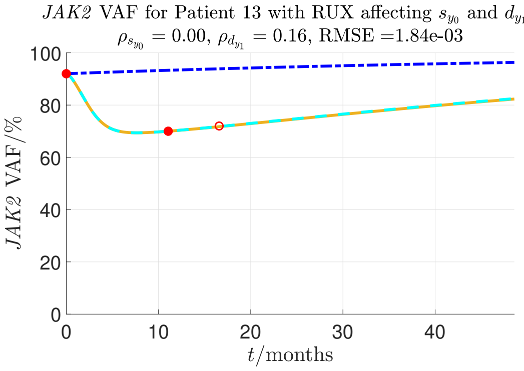


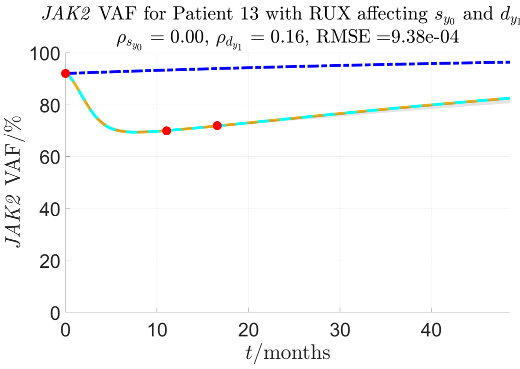

## 6.14 Patient 14

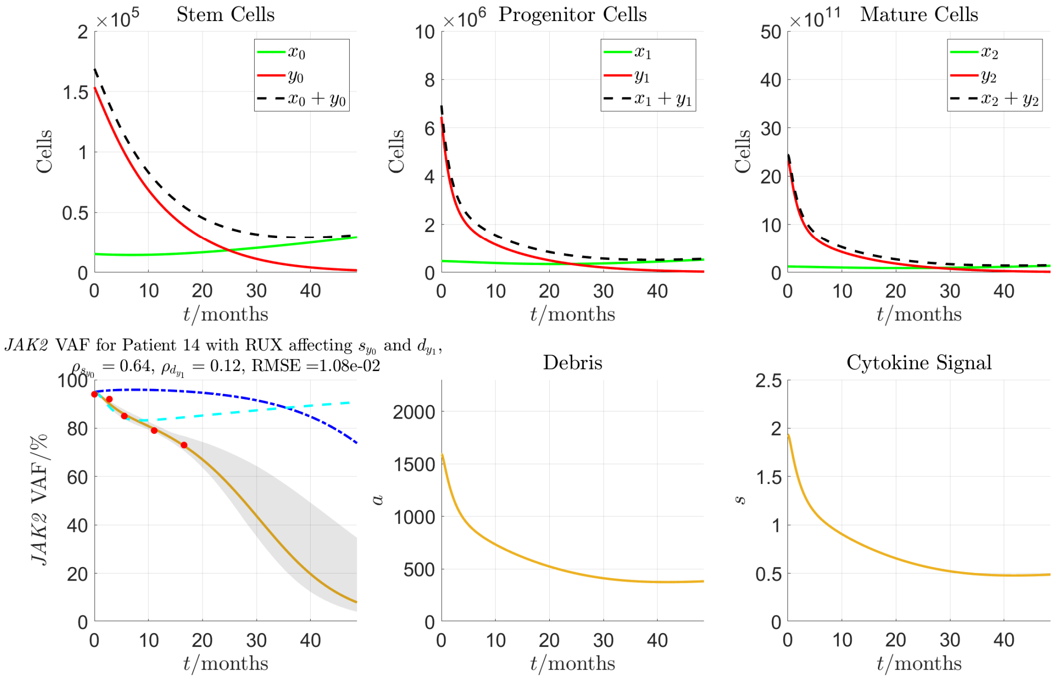


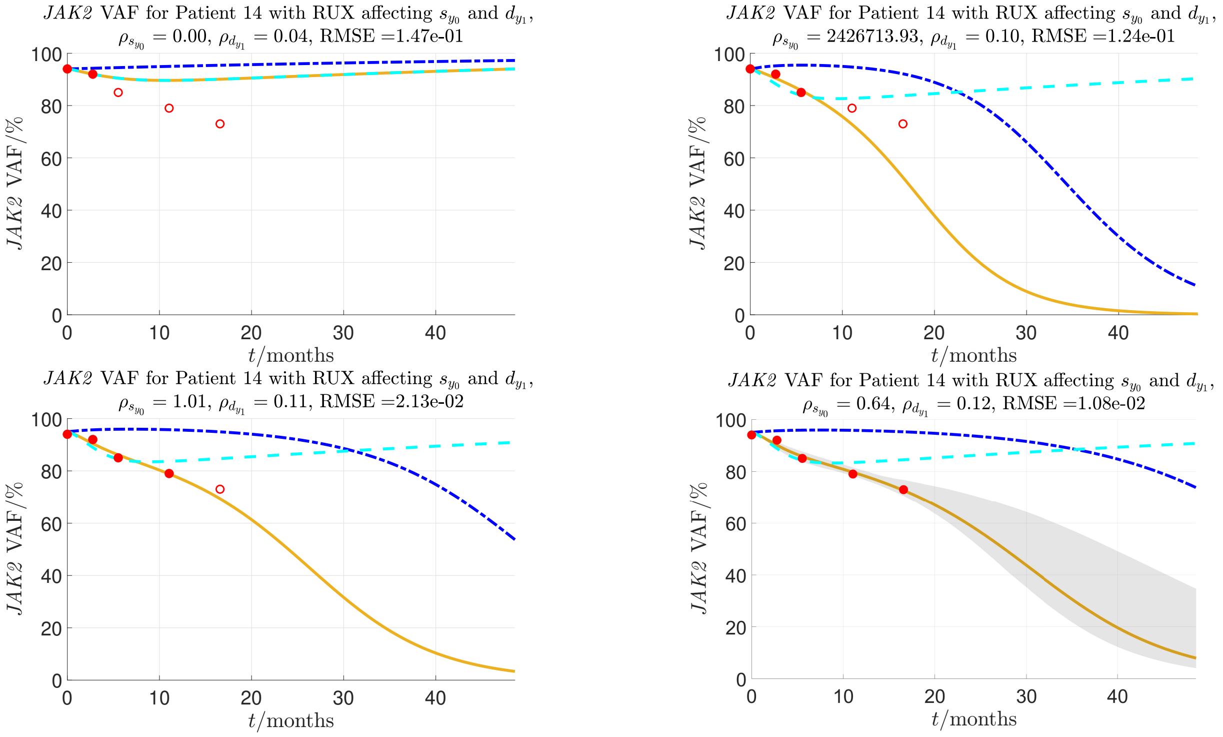

## 6.15 Patient 15

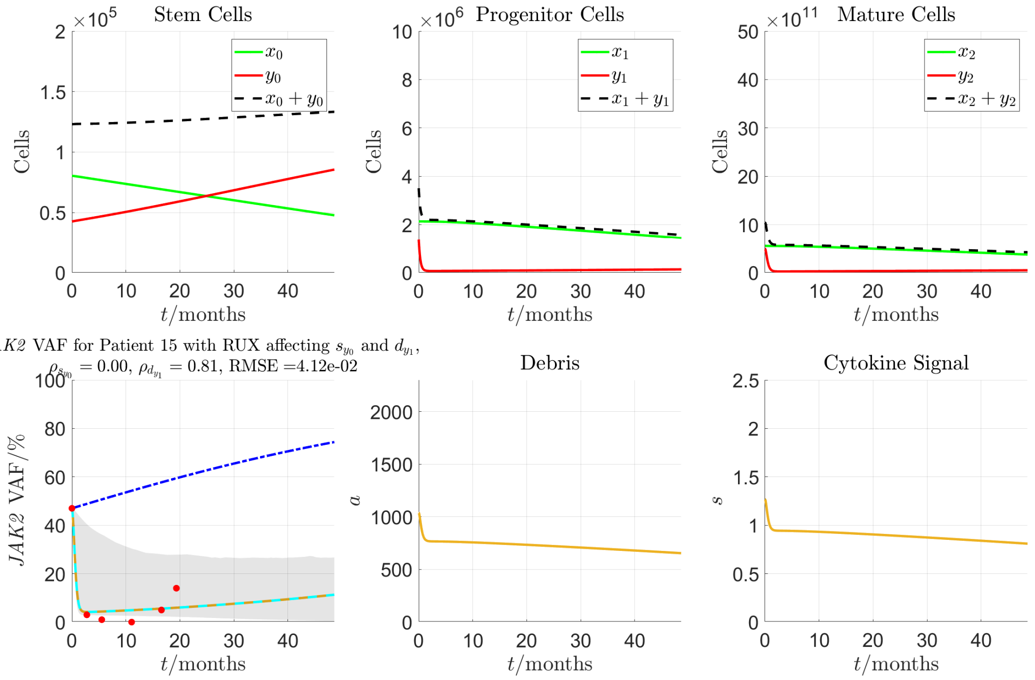

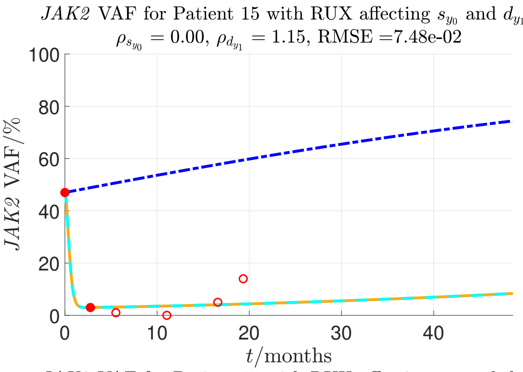
*JAK2* VAF for Patient 15 with RUX affecting $s_{y_0}$ and $d_{y_1}$,
$\rho_{s_{y_0}} = 0.00$, $\rho_{d_{y_1}} = 1.15$, RMSE =7.48e-02
*JAK2* VAF/%
$t$/months

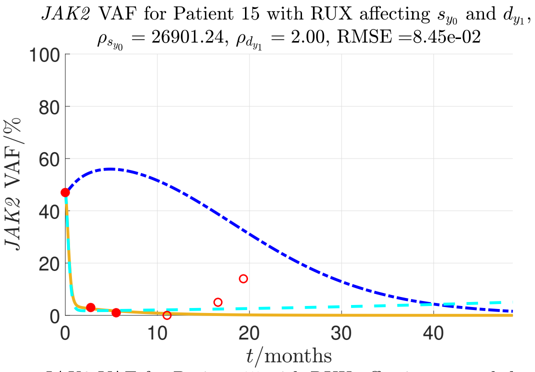
*JAK2* VAF for Patient 15 with RUX affecting $s_{y_0}$ and $d_{y_1}$,
$\rho_{s_{y_0}} = 26901.24$, $\rho_{d_{y_1}} = 2.00$, RMSE =8.45e-02
*JAK2* VAF/%
$t$/months

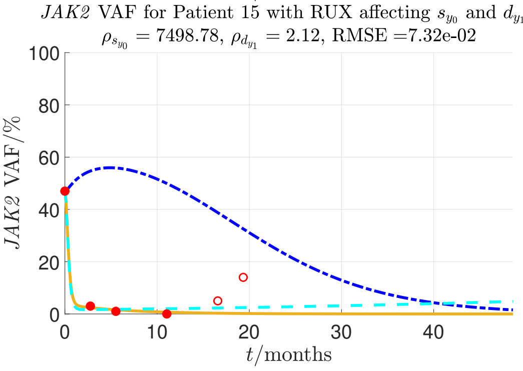
*JAK2* VAF for Patient 15 with RUX affecting $s_{y_0}$ and $d_{y_1}$,
$\rho_{s_{y_0}} = 7498.78$, $\rho_{d_{y_1}} = 2.12$, RMSE =7.32e-02
*JAK2* VAF/%
$t$/months

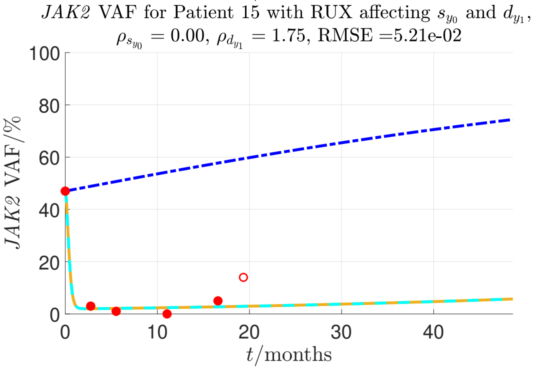
*JAK2* VAF for Patient 15 with RUX affecting $s_{y_0}$ and $d_{y_1}$,
$\rho_{s_{y_0}} = 0.00$, $\rho_{d_{y_1}} = 1.75$, RMSE =5.21e-02
*JAK2* VAF/%
$t$/months

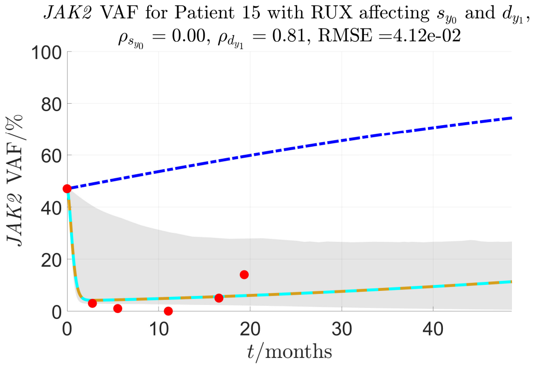
*JAK2* VAF for Patient 15 with RUX affecting $s_{y_0}$ and $d_{y_1}$,
$\rho_{s_{y_0}} = 0.00$, $\rho_{d_{y_1}} = 0.81$, RMSE =4.12e-02
*JAK2* VAF/%
$t$/months

## 6.16 Patient 16

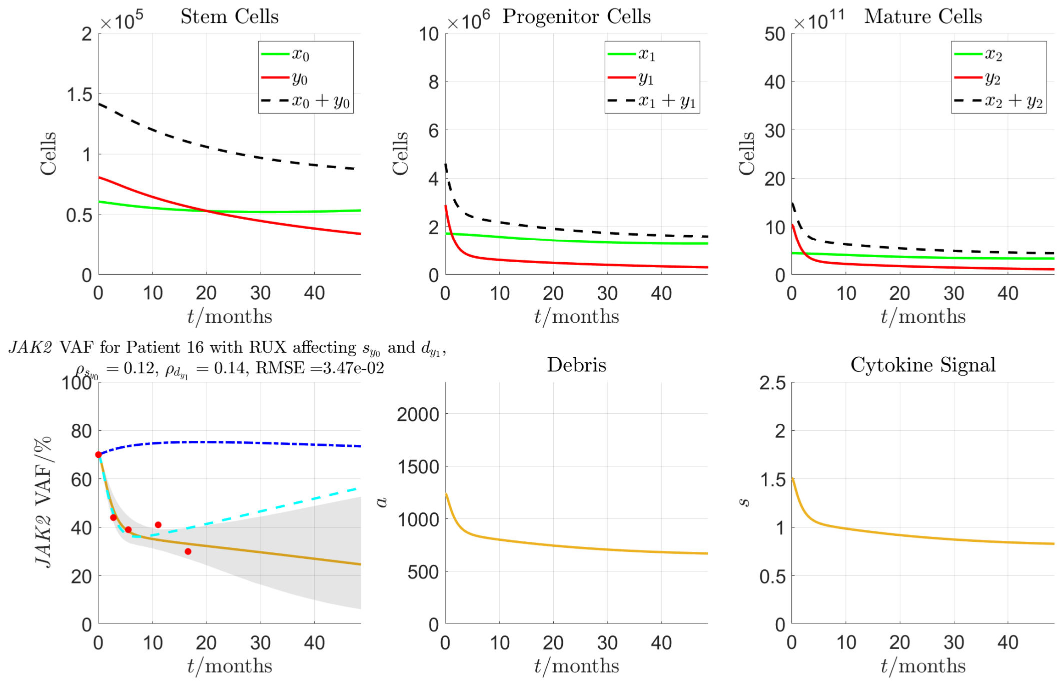


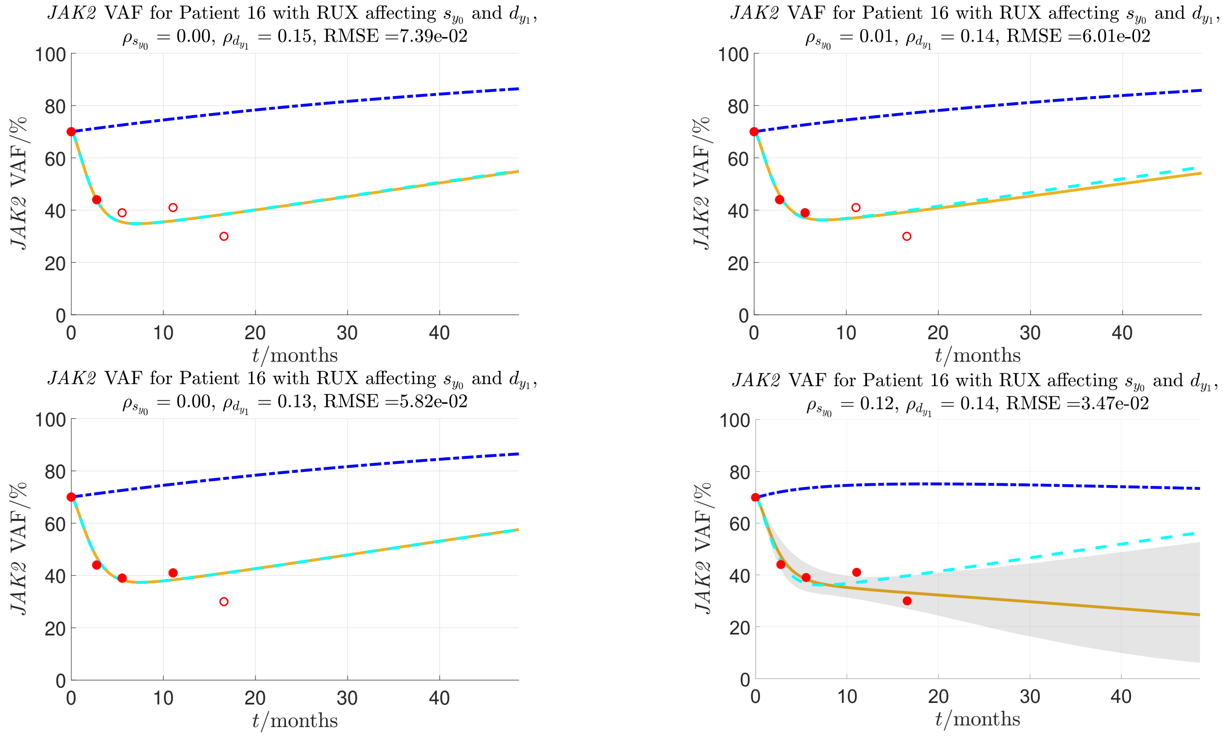

## 6.17 Patient 17

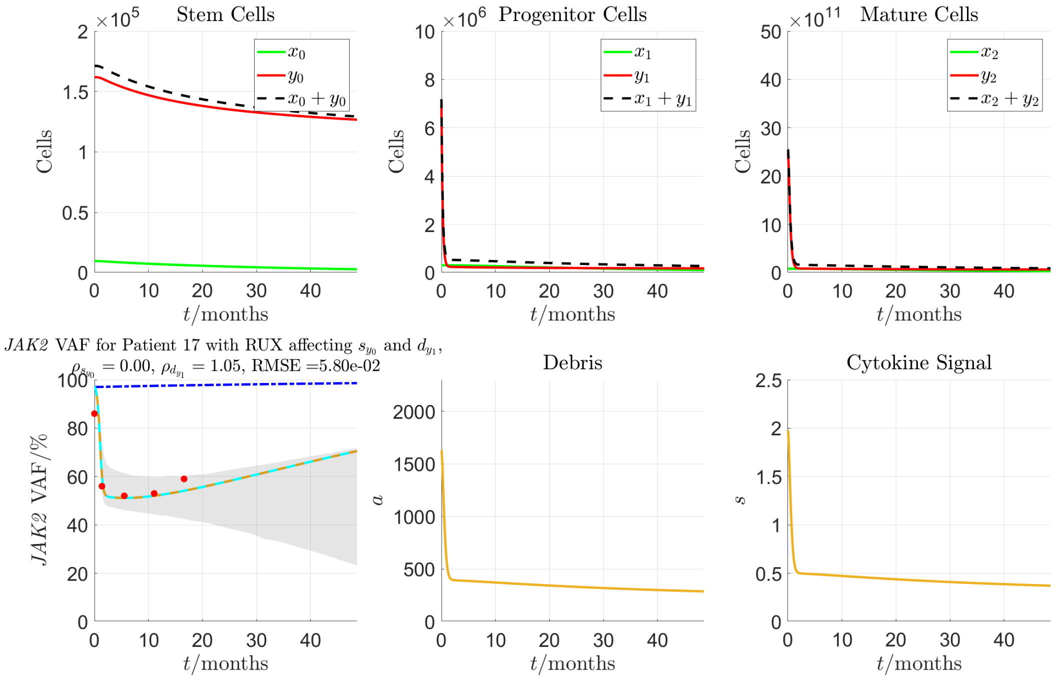


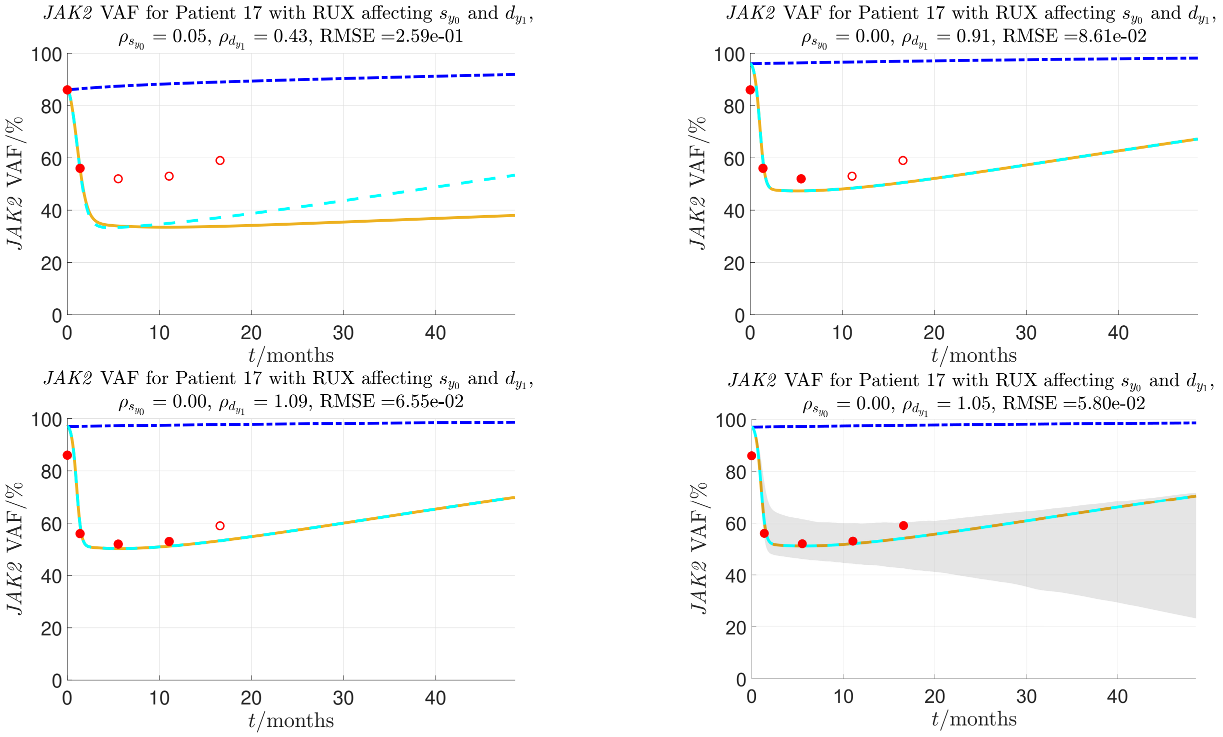

## 6.18 Patient 18

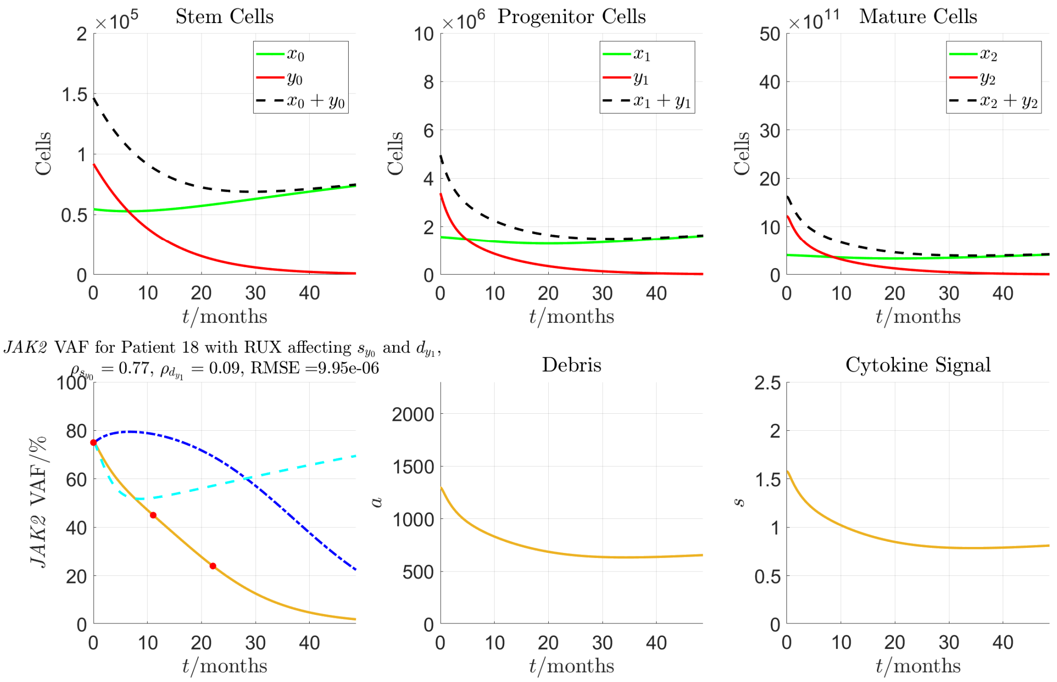


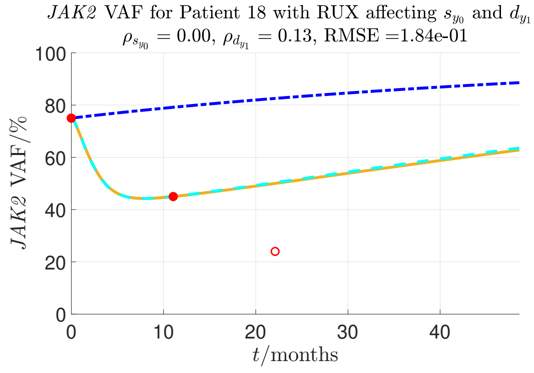


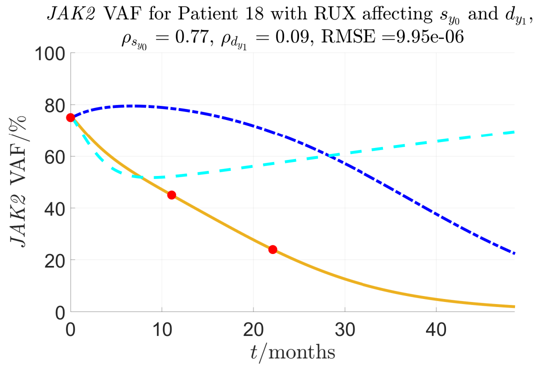

## 6.19 Patient 19

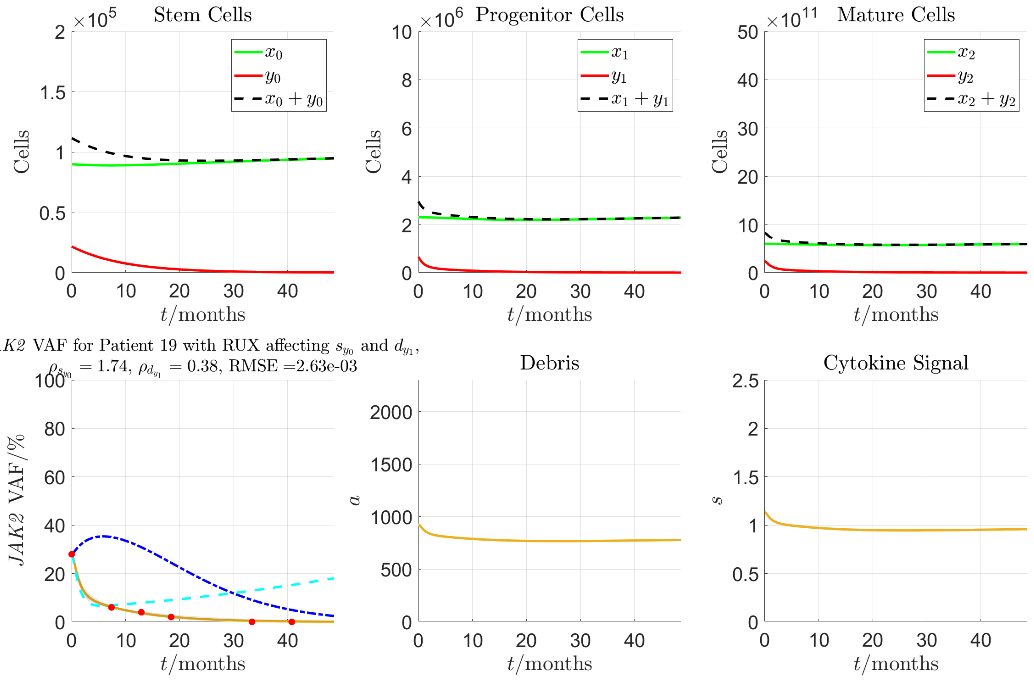

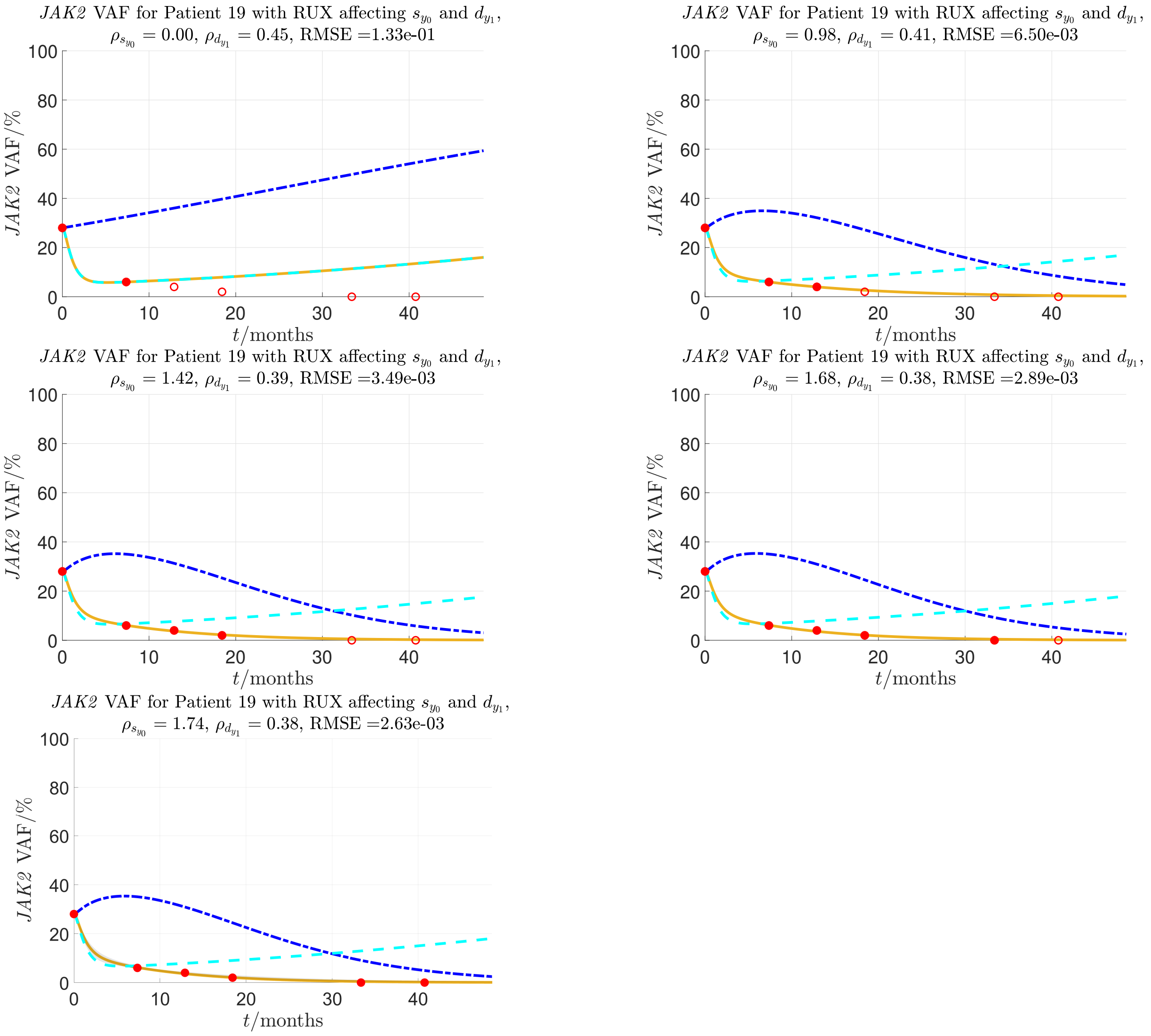
*JAK2* VAF for Patient 19 with RUX affecting $s_{y_0}$ and $d_{y_1}$,
$\rho_{s_{y_0}} = 0.00$, $\rho_{d_{y_1}} = 0.45$, RMSE =1.33e-01
*JAK2* VAF for Patient 19 with RUX affecting $s_{y_0}$ and $d_{y_1}$,
$\rho_{s_{y_0}} = 0.98$, $\rho_{d_{y_1}} = 0.41$, RMSE =6.50e-03
*JAK2* VAF for Patient 19 with RUX affecting $s_{y_0}$ and $d_{y_1}$,
$\rho_{s_{y_0}} = 1.42$, $\rho_{d_{y_1}} = 0.39$, RMSE =3.49e-03
*JAK2* VAF for Patient 19 with RUX affecting $s_{y_0}$ and $d_{y_1}$,
$\rho_{s_{y_0}} = 1.68$, $\rho_{d_{y_1}} = 0.38$, RMSE =2.89e-03
*JAK2* VAF for Patient 19 with RUX affecting $s_{y_0}$ and $d_{y_1}$,
$\rho_{s_{y_0}} = 1.74$, $\rho_{d_{y_1}} = 0.38$, RMSE =2.63e-03
*JAK2* VAF/%
$t$/months

## 6.20 Patient 20

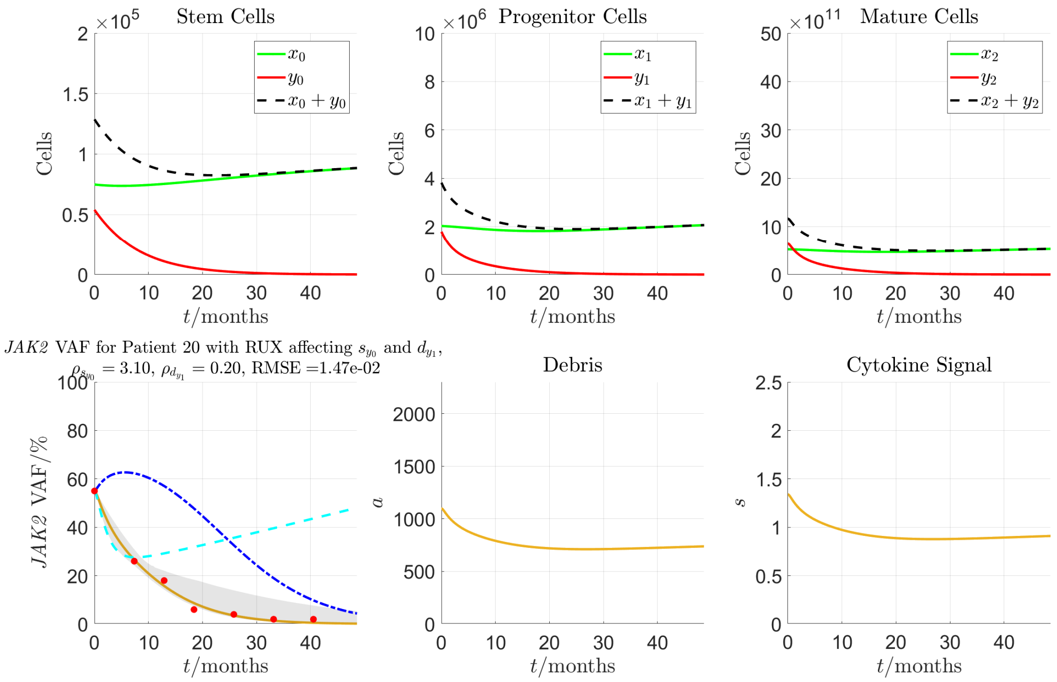

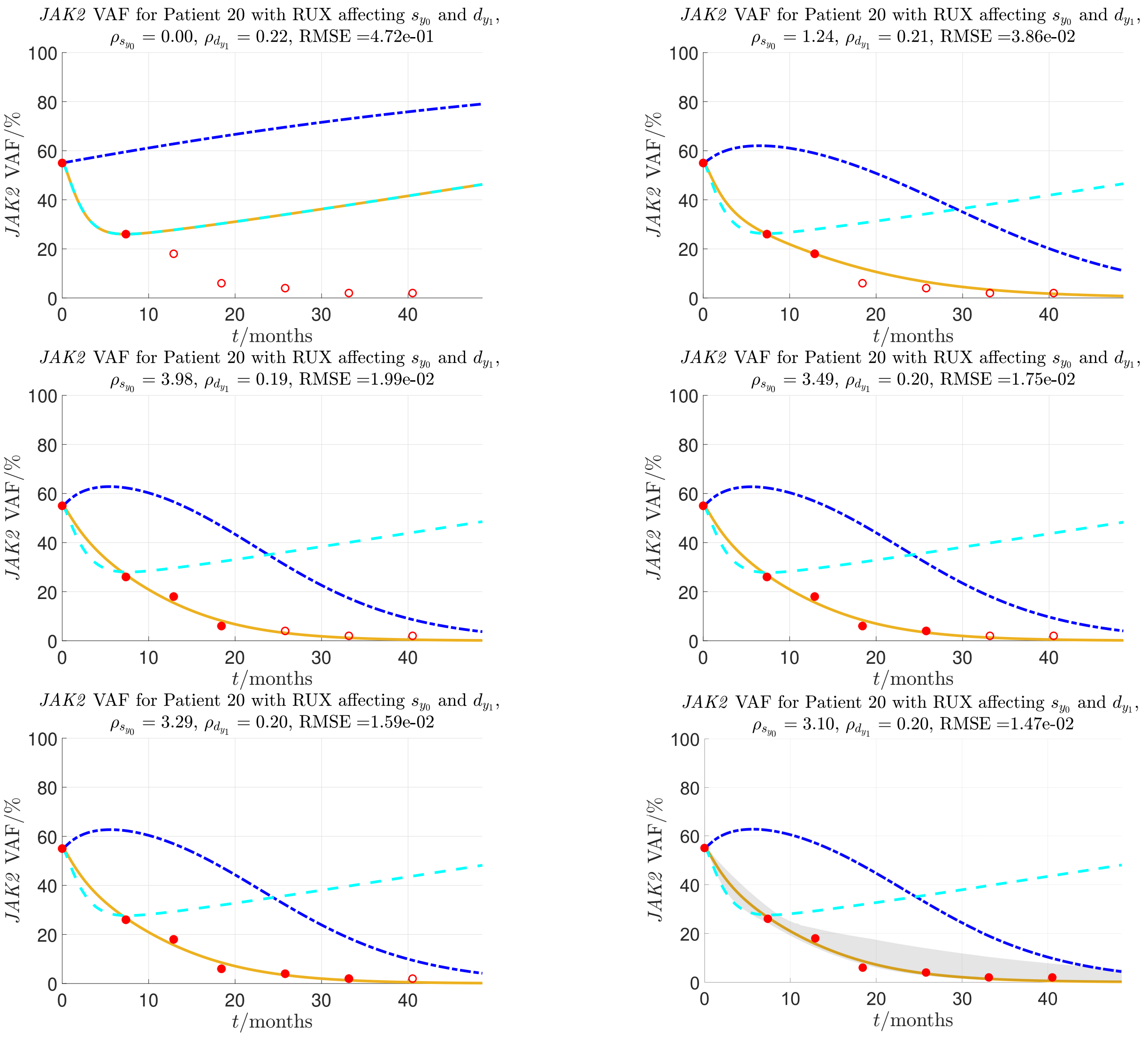
*JAK2* VAF for Patient 20 with RUX affecting $s_{y_0}$ and $d_{y_1}$,
$\rho_{s_{y_0}} = 0.00$, $\rho_{d_{y_1}} = 0.22$, RMSE =4.72e-01
*JAK2* VAF for Patient 20 with RUX affecting $s_{y_0}$ and $d_{y_1}$,
$\rho_{s_{y_0}} = 1.24$, $\rho_{d_{y_1}} = 0.21$, RMSE =3.86e-02
*JAK2* VAF for Patient 20 with RUX affecting $s_{y_0}$ and $d_{y_1}$,
$\rho_{s_{y_0}} = 3.98$, $\rho_{d_{y_1}} = 0.19$, RMSE =1.99e-02
*JAK2* VAF for Patient 20 with RUX affecting $s_{y_0}$ and $d_{y_1}$,
$\rho_{s_{y_0}} = 3.49$, $\rho_{d_{y_1}} = 0.20$, RMSE =1.75e-02
*JAK2* VAF for Patient 20 with RUX affecting $s_{y_0}$ and $d_{y_1}$,
$\rho_{s_{y_0}} = 3.29$, $\rho_{d_{y_1}} = 0.20$, RMSE =1.59e-02
*JAK2* VAF for Patient 20 with RUX affecting $s_{y_0}$ and $d_{y_1}$,
$\rho_{s_{y_0}} = 3.10$, $\rho_{d_{y_1}} = 0.20$, RMSE =1.47e-02
*JAK2* VAF/%
$t$/months

## 6.21 Patient 21

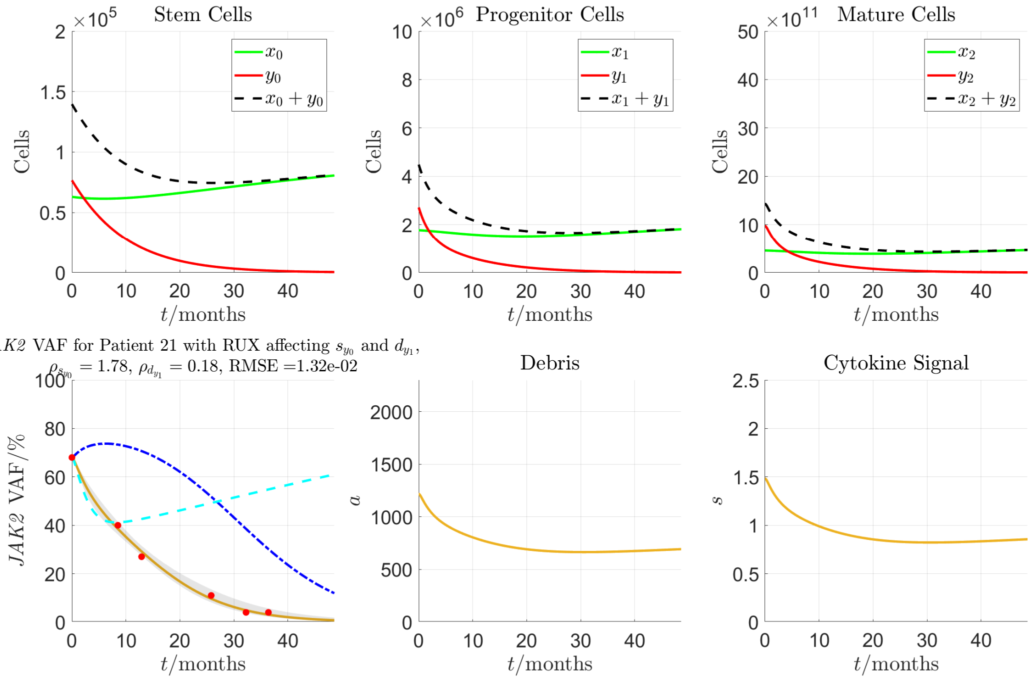

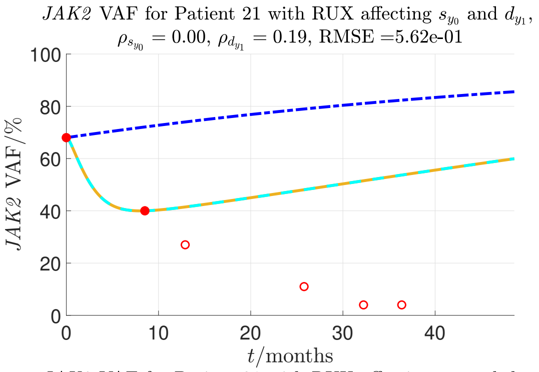
*JAK2* VAF for Patient 21 with RUX affecting $s_{y_0}$ and $d_{y_1}$,
$\rho_{s_{y_0}} = 0.00$, $\rho_{d_{y_1}} = 0.19$, RMSE =5.62e-01
*JAK2* VAF/%
$t$/months

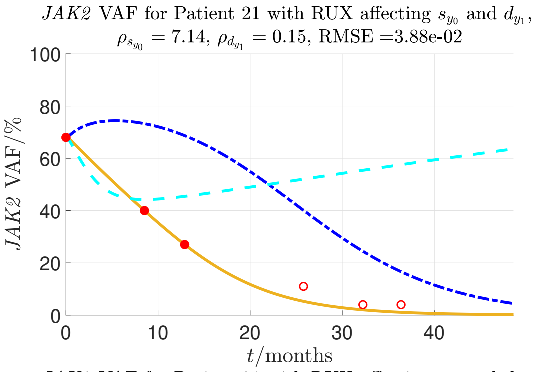
*JAK2* VAF for Patient 21 with RUX affecting $s_{y_0}$ and $d_{y_1}$,
$\rho_{s_{y_0}} = 7.14$, $\rho_{d_{y_1}} = 0.15$, RMSE =3.88e-02
*JAK2* VAF/%
$t$/months

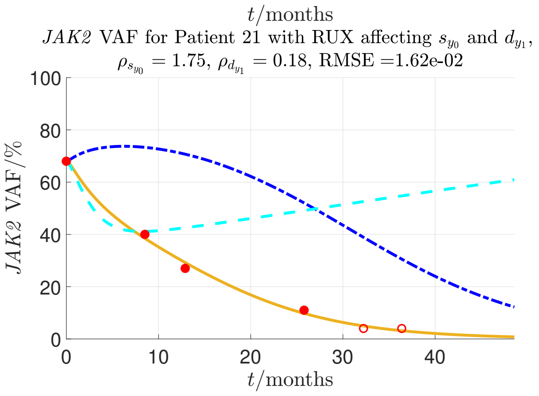
*JAK2* VAF for Patient 21 with RUX affecting $s_{y_0}$ and $d_{y_1}$,
$\rho_{s_{y_0}} = 1.75$, $\rho_{d_{y_1}} = 0.18$, RMSE =1.62e-02
*JAK2* VAF/%
$t$/months

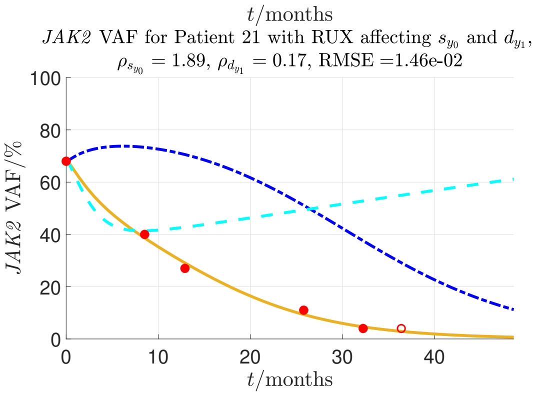
*JAK2* VAF for Patient 21 with RUX affecting $s_{y_0}$ and $d_{y_1}$,
$\rho_{s_{y_0}} = 1.89$, $\rho_{d_{y_1}} = 0.17$, RMSE =1.46e-02
*JAK2* VAF/%
$t$/months

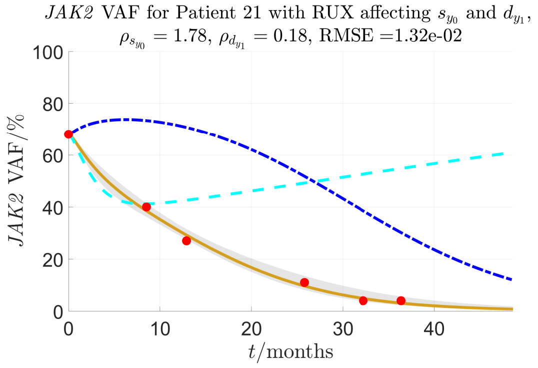
*JAK2* VAF for Patient 21 with RUX affecting $s_{y_0}$ and $d_{y_1}$,
$\rho_{s_{y_0}} = 1.78$, $\rho_{d_{y_1}} = 0.18$, RMSE =1.32e-02
*JAK2* VAF/%
$t$/months

## 6.22 Patient 22

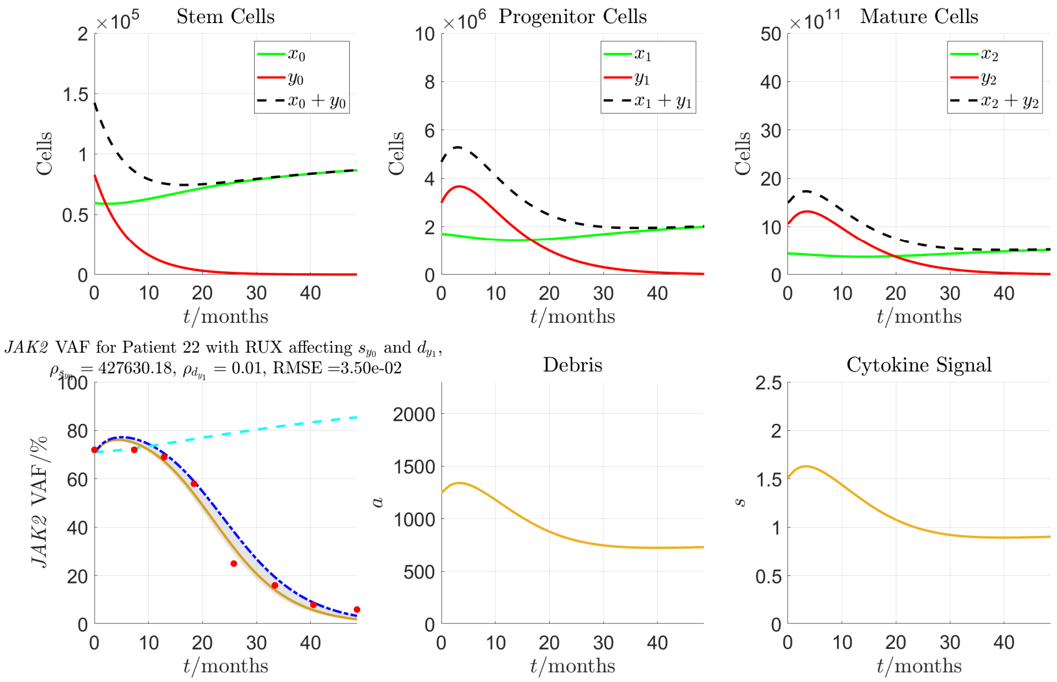

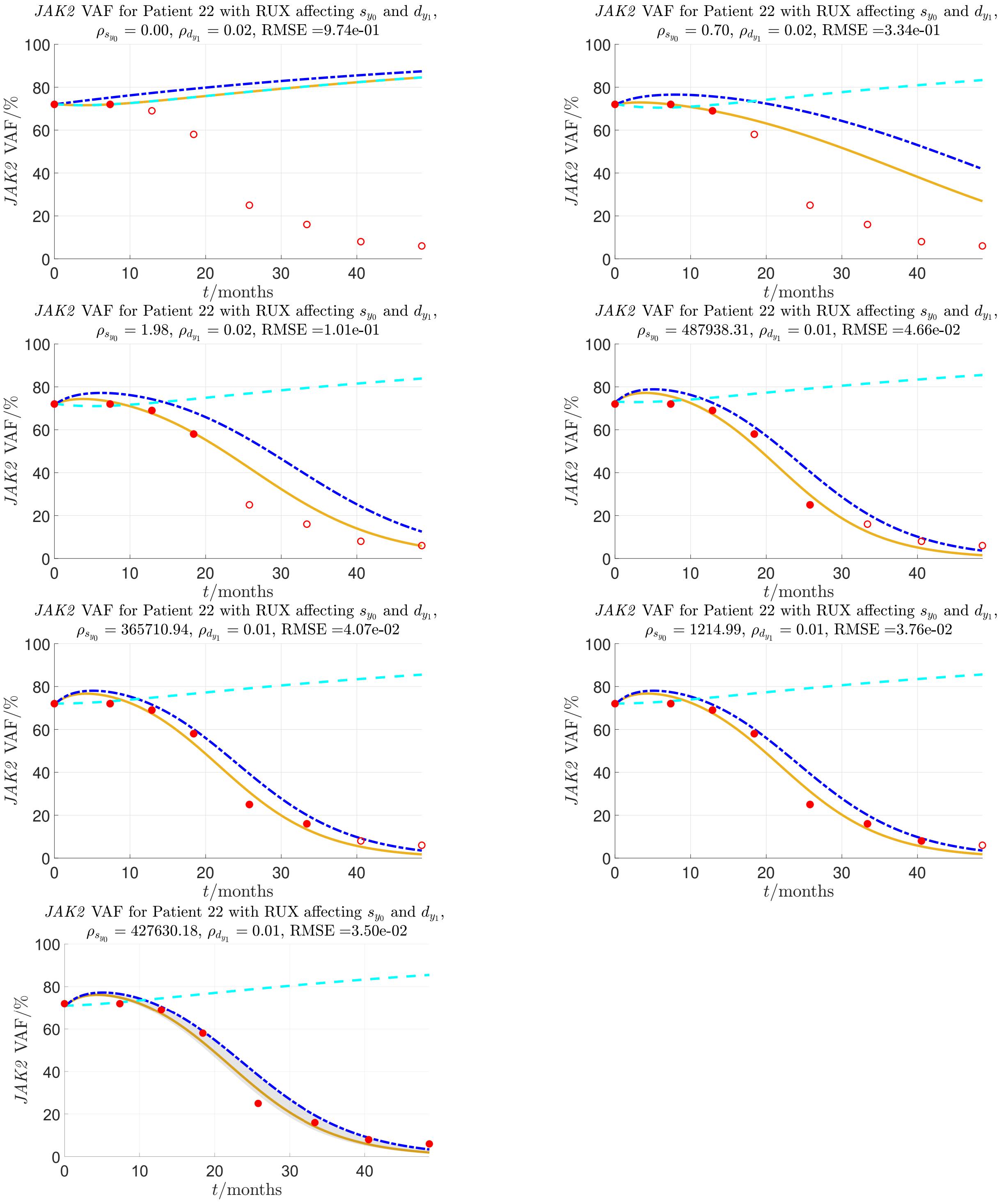

JAK2 VAF for Patient 22 with RUX affecting $s_{y_0}$ and $d_{y_1}$, $\rho_{s_{y_0}} = 0.00$, $\rho_{d_{y_1}} = 0.02$, RMSE =9.74e-01
JAK2 VAF for Patient 22 with RUX affecting $s_{y_0}$ and $d_{y_1}$, $\rho_{s_{y_0}} = 0.70$, $\rho_{d_{y_1}} = 0.02$, RMSE =3.34e-01
JAK2 VAF for Patient 22 with RUX affecting $s_{y_0}$ and $d_{y_1}$, $\rho_{s_{y_0}} = 1.98$, $\rho_{d_{y_1}} = 0.02$, RMSE =1.01e-01
JAK2 VAF for Patient 22 with RUX affecting $s_{y_0}$ and $d_{y_1}$, $\rho_{s_{y_0}} = 487938.31$, $\rho_{d_{y_1}} = 0.01$, RMSE =4.66e-02
JAK2 VAF for Patient 22 with RUX affecting $s_{y_0}$ and $d_{y_1}$, $\rho_{s_{y_0}} = 365710.94$, $\rho_{d_{y_1}} = 0.01$, RMSE =4.07e-02
JAK2 VAF for Patient 22 with RUX affecting $s_{y_0}$ and $d_{y_1}$, $\rho_{s_{y_0}} = 1214.99$, $\rho_{d_{y_1}} = 0.01$, RMSE =3.76e-02
JAK2 VAF for Patient 22 with RUX affecting $s_{y_0}$ and $d_{y_1}$, $\rho_{s_{y_0}} = 427630.18$, $\rho_{d_{y_1}} = 0.01$, RMSE =3.50e-02
JAK2 VAF/%
$t$/months

## 6.23 Patient 23

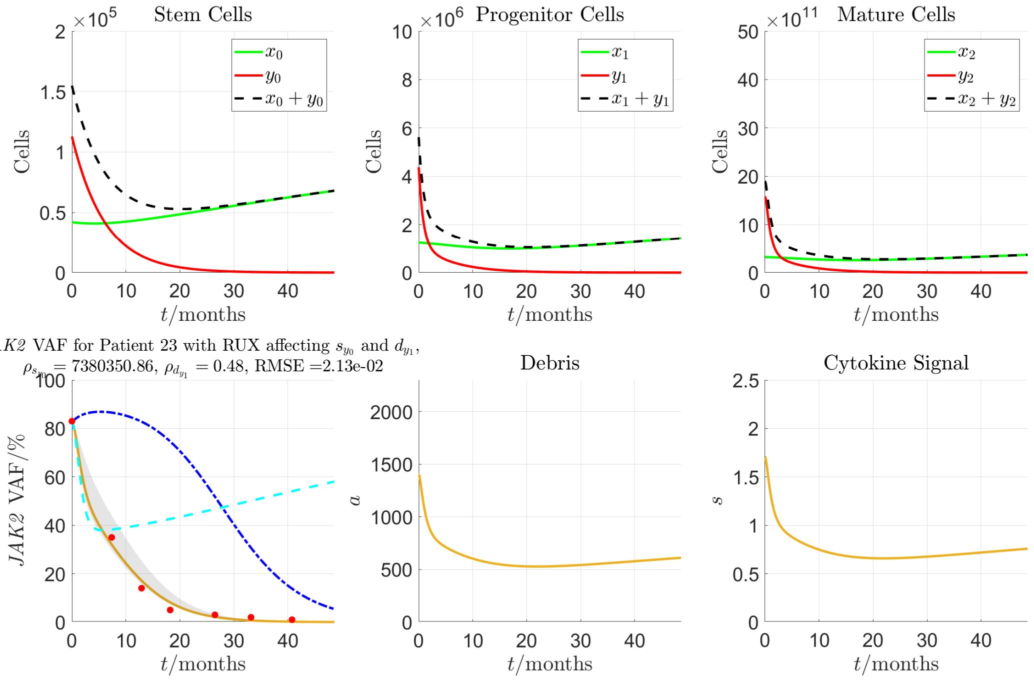

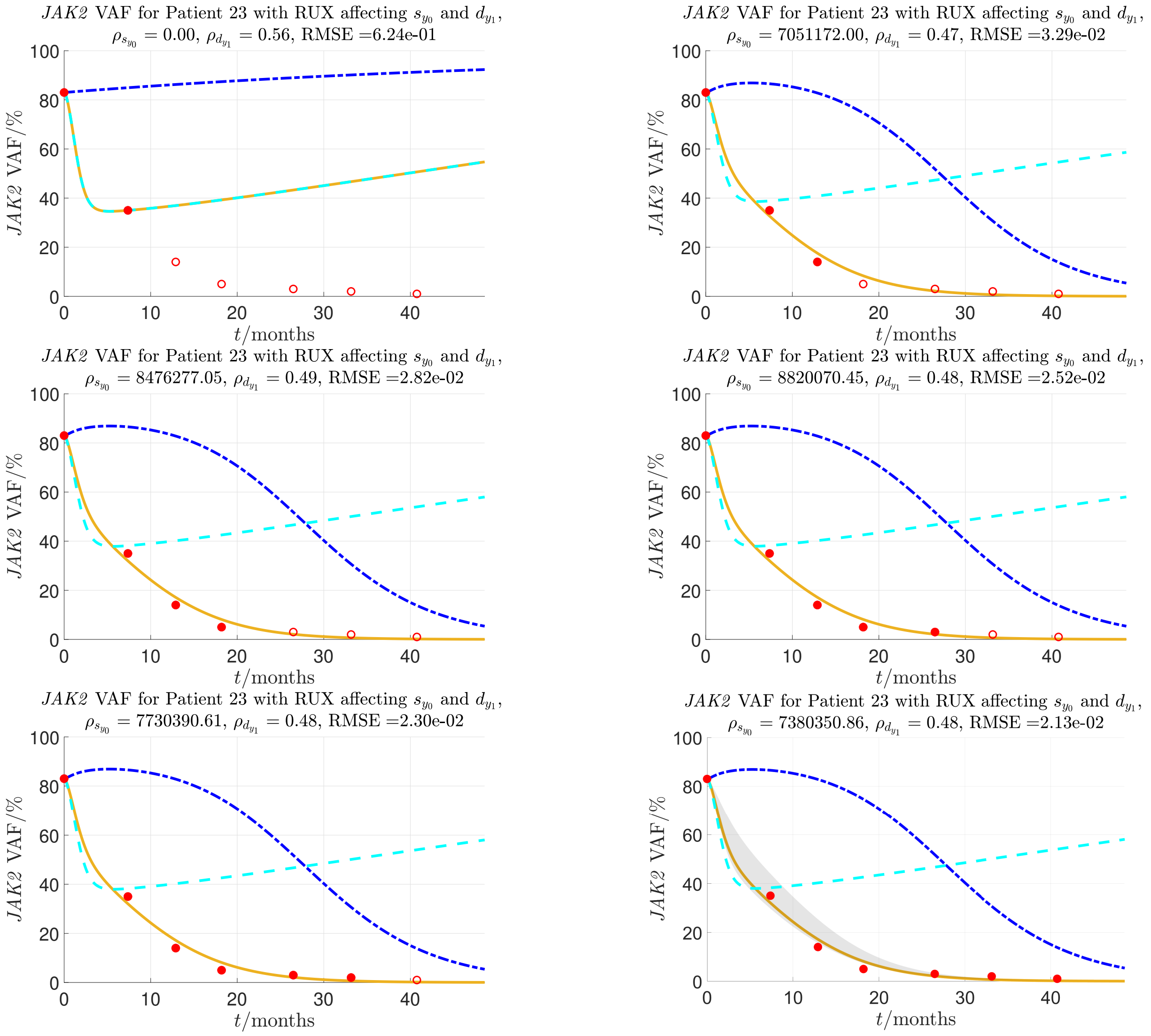
JAK2 VAF for Patient 23 with RUX affecting $s_{y_0}$ and $d_{y_1}$,
$\rho_{s_{y_0}} = 0.00$, $\rho_{d_{y_1}} = 0.56$, RMSE =6.24e-01
JAK2 VAF for Patient 23 with RUX affecting $s_{y_0}$ and $d_{y_1}$,
$\rho_{s_{y_0}} = 7051172.00$, $\rho_{d_{y_1}} = 0.47$, RMSE =3.29e-02
JAK2 VAF for Patient 23 with RUX affecting $s_{y_0}$ and $d_{y_1}$,
$\rho_{s_{y_0}} = 8476277.05$, $\rho_{d_{y_1}} = 0.49$, RMSE =2.82e-02
JAK2 VAF for Patient 23 with RUX affecting $s_{y_0}$ and $d_{y_1}$,
$\rho_{s_{y_0}} = 8820070.45$, $\rho_{d_{y_1}} = 0.48$, RMSE =2.52e-02
JAK2 VAF for Patient 23 with RUX affecting $s_{y_0}$ and $d_{y_1}$,
$\rho_{s_{y_0}} = 7730390.61$, $\rho_{d_{y_1}} = 0.48$, RMSE =2.30e-02
JAK2 VAF for Patient 23 with RUX affecting $s_{y_0}$ and $d_{y_1}$,
$\rho_{s_{y_0}} = 7380350.86$, $\rho_{d_{y_1}} = 0.48$, RMSE =2.13e-02
JAK2 VAF/%
$t$/months

## 6.24 Patient 24

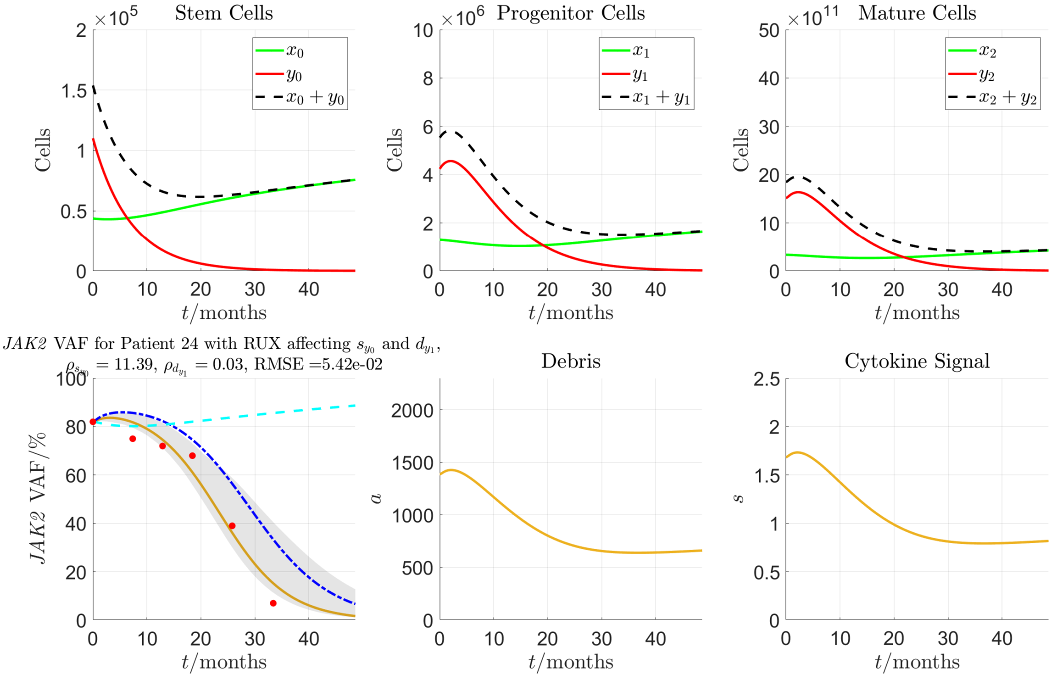

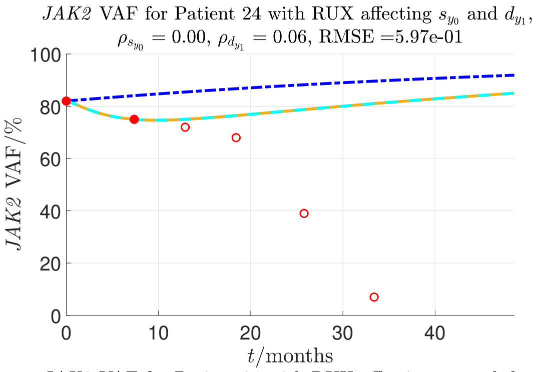
*JAK2* VAF for Patient 24 with RUX affecting $s_{y_0}$ and $d_{y_1}$,
$\rho_{s_{y_0}} = 0.00$, $\rho_{d_{y_1}} = 0.06$, RMSE =5.97e-01
*JAK2* VAF/%
$t$/months

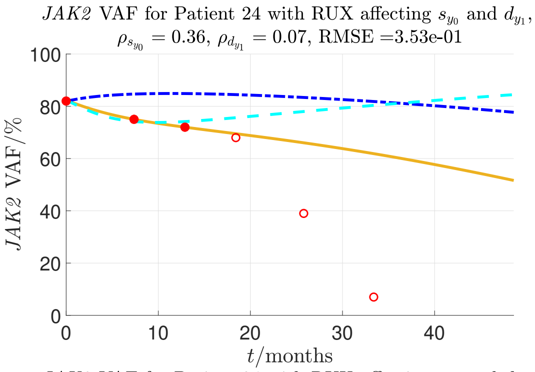
*JAK2* VAF for Patient 24 with RUX affecting $s_{y_0}$ and $d_{y_1}$,
$\rho_{s_{y_0}} = 0.36$, $\rho_{d_{y_1}} = 0.07$, RMSE =3.53e-01
*JAK2* VAF/%
$t$/months

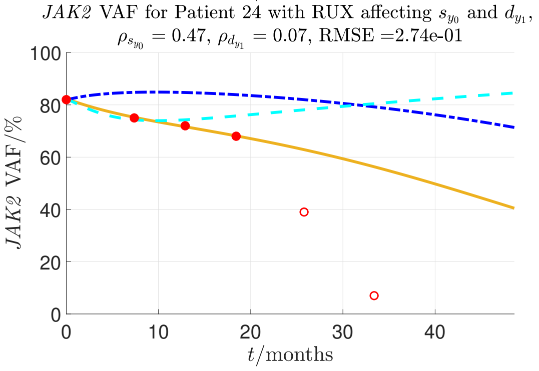
*JAK2* VAF for Patient 24 with RUX affecting $s_{y_0}$ and $d_{y_1}$,
$\rho_{s_{y_0}} = 0.47$, $\rho_{d_{y_1}} = 0.07$, RMSE =2.74e-01
*JAK2* VAF/%
$t$/months

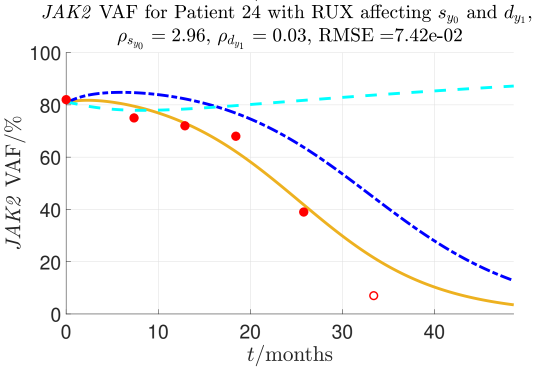
*JAK2* VAF for Patient 24 with RUX affecting $s_{y_0}$ and $d_{y_1}$,
$\rho_{s_{y_0}} = 2.96$, $\rho_{d_{y_1}} = 0.03$, RMSE =7.42e-02
*JAK2* VAF/%
$t$/months

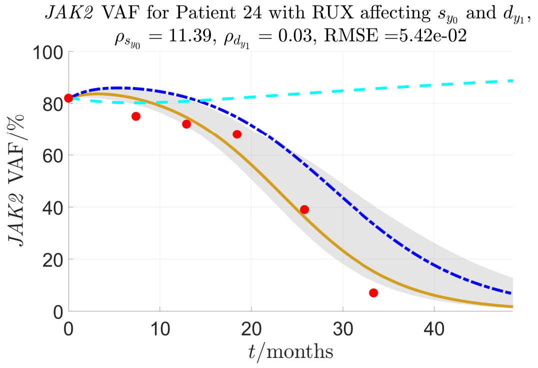
*JAK2* VAF for Patient 24 with RUX affecting $s_{y_0}$ and $d_{y_1}$,
$\rho_{s_{y_0}} = 11.39$, $\rho_{d_{y_1}} = 0.03$, RMSE =5.42e-02
*JAK2* VAF/%
$t$/months

# 7 FITTING WITH ONLY $s_{y_0}$ OR $d_{y_1}$

In this section, we present the plots of the fitting of the model to the individual patient data for all 24 patients when allowing the RUX treatment to affect only $s_{y0}$ or $d_{y1}$ (and not both). The plots with RUX affecting only $s_{y0}$ are shown to the left (in blue), and the plots with RUX affecting only $d_{y1}$ are shown to the right (in cyan). The blue/cyan curves show the model dynamics and the filled red dots are the data points used in the fit.

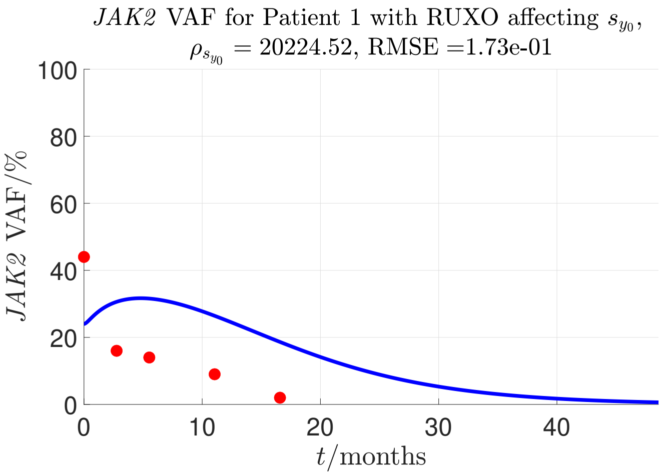


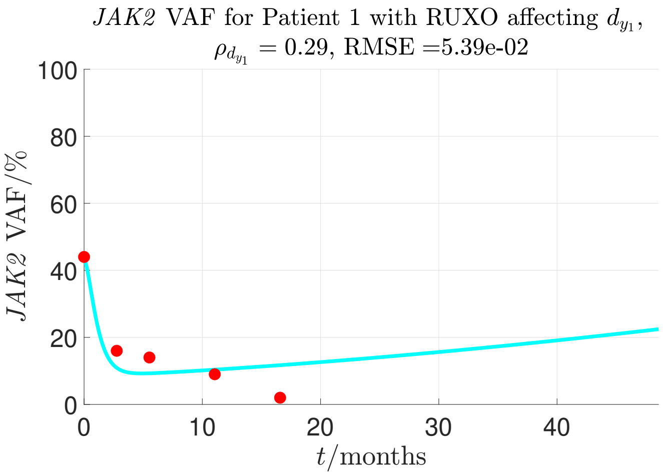


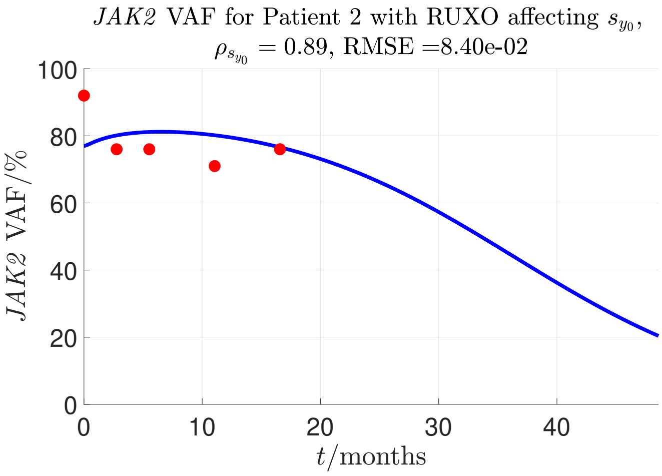


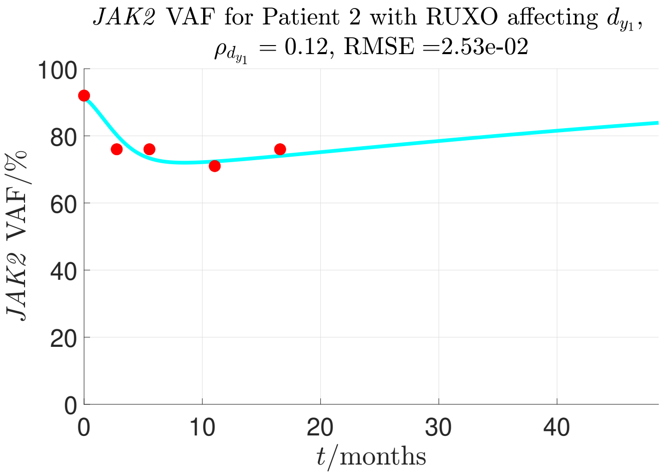

*JAK2* VAF for Patient 3 with RUXO affecting $s_{y_0}$, $\rho_{s_{y_0}} = 0.62$, RMSE =1.42e-01

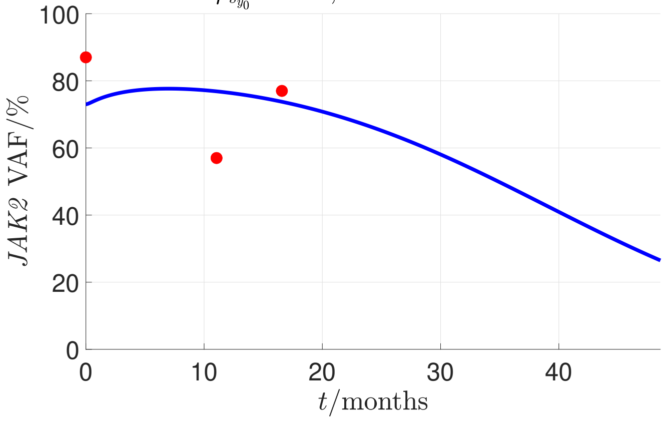


*JAK2* VAF for Patient 3 with RUXO affecting $d_{y_1}$, $\rho_{d_{y_1}} = 0.11$, RMSE =7.39e-02

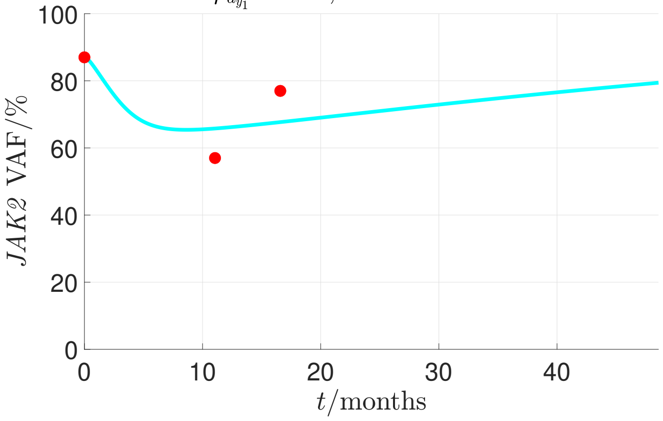


*JAK2* VAF for Patient 4 with RUXO affecting $s_{y_0}$, $\rho_{s_{y_0}} = 30037.35$, RMSE =1.76e-01

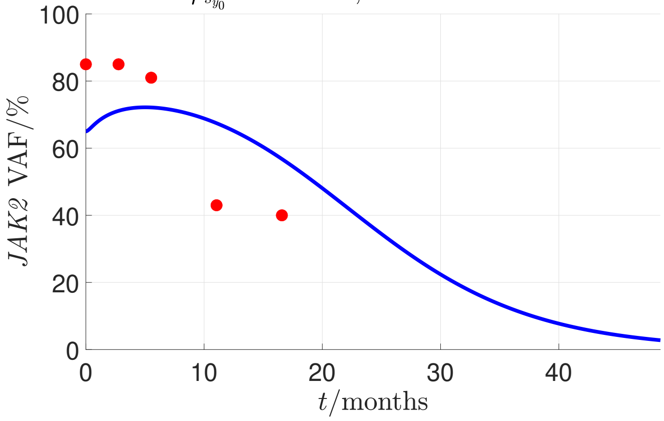


*JAK2* VAF for Patient 4 with RUXO affecting $d_{y_1}$, $\rho_{d_{y_1}} = 0.12$, RMSE =1.69e-01

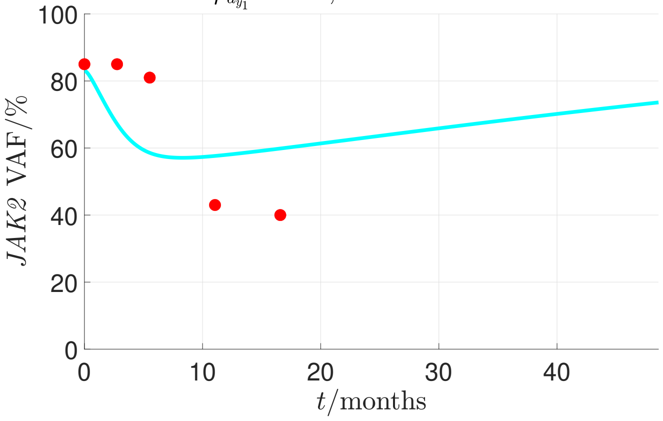


*JAK2* VAF for Patient 5 with RUXO affecting $s_{y_0}$, $\rho_{s_{y_0}} = 25863.81$, RMSE =1.24e-01

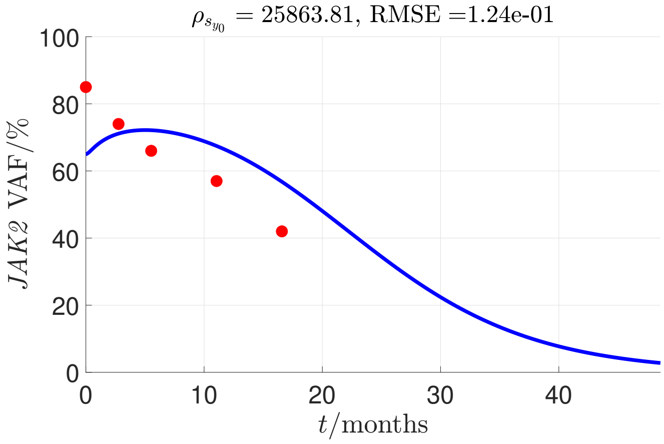


*JAK2* VAF for Patient 5 with RUXO affecting $d_{y_1}$, $\rho_{d_{y_1}} = 550.25$, RMSE =8.14e-02

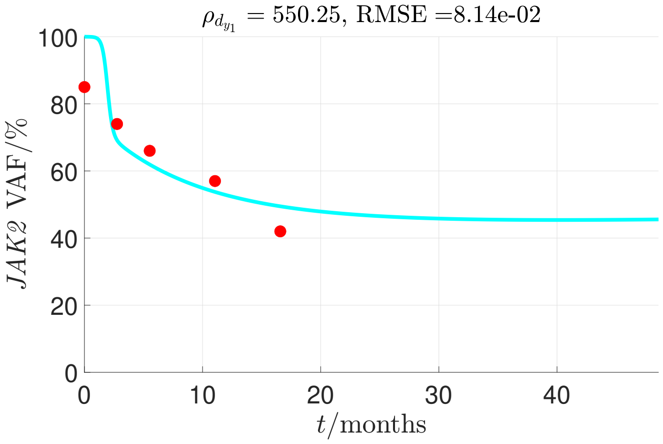

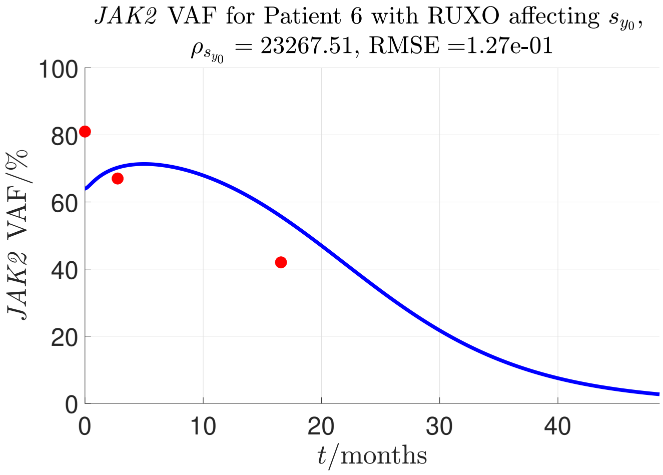
*JAK2* VAF for Patient 6 with RUXO affecting $s_{y_0}$,
$\rho_{s_{y_0}} = 23267.51$, RMSE =1.27e-01
*JAK2* VAF/%
$t$/months

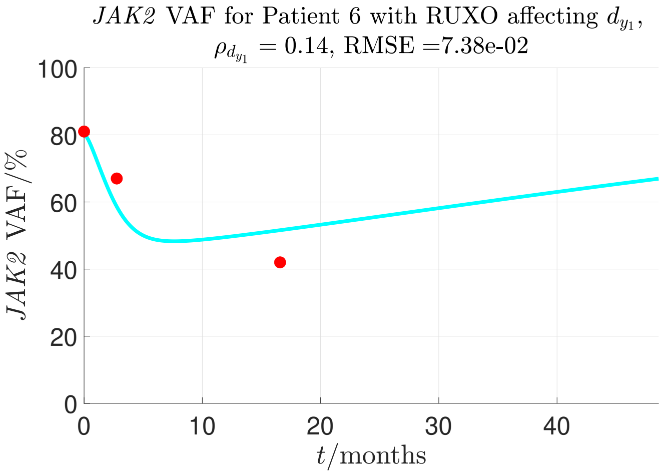
*JAK2* VAF for Patient 6 with RUXO affecting $d_{y_1}$,
$\rho_{d_{y_1}} = 0.14$, RMSE =7.38e-02
*JAK2* VAF/%
$t$/months

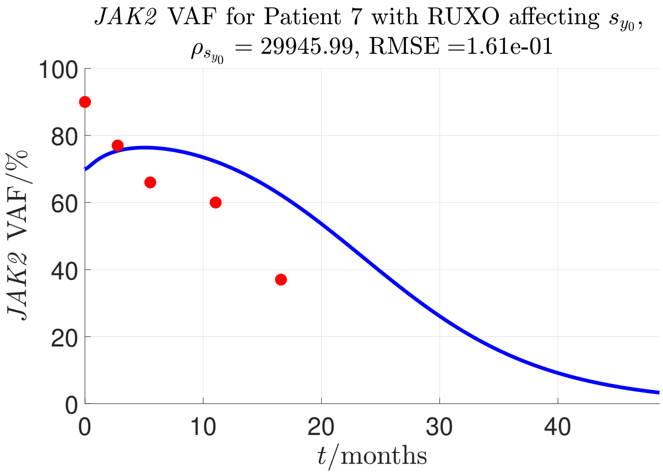
*JAK2* VAF for Patient 7 with RUXO affecting $s_{y_0}$,
$\rho_{s_{y_0}} = 29945.99$, RMSE =1.61e-01
*JAK2* VAF/%
$t$/months

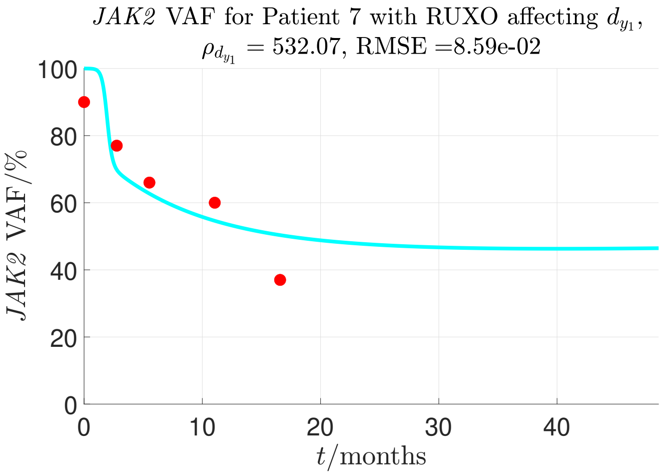
*JAK2* VAF for Patient 7 with RUXO affecting $d_{y_1}$,
$\rho_{d_{y_1}} = 532.07$, RMSE =8.59e-02
*JAK2* VAF/%
$t$/months

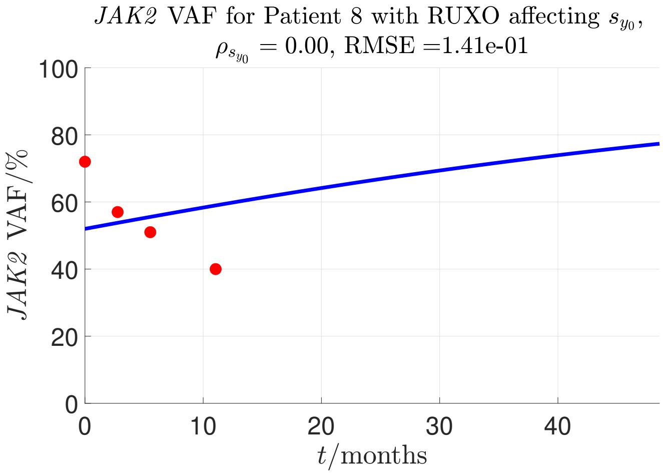
*JAK2* VAF for Patient 8 with RUXO affecting $s_{y_0}$,
$\rho_{s_{y_0}} = 0.00$, RMSE =1.41e-01
*JAK2* VAF/%
$t$/months

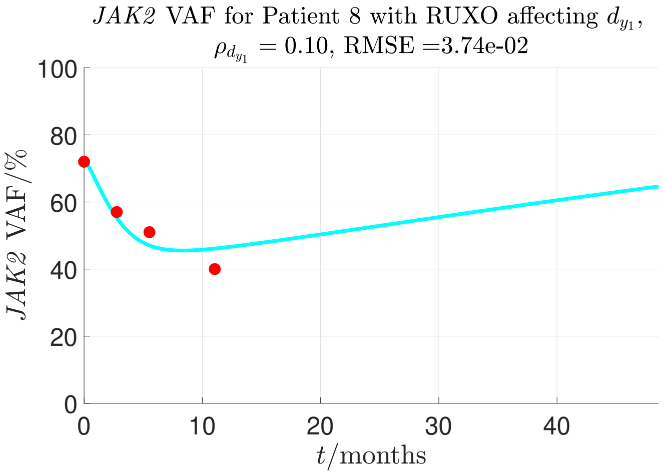
*JAK2* VAF for Patient 8 with RUXO affecting $d_{y_1}$,
$\rho_{d_{y_1}} = 0.10$, RMSE =3.74e-02
*JAK2* VAF/%
$t$/months

*JAK2* VAF for Patient 9 with RUXO affecting $s_{y_0}$, $\rho_{s_{y_0}}$ = 22640.55, RMSE =1.35e-01

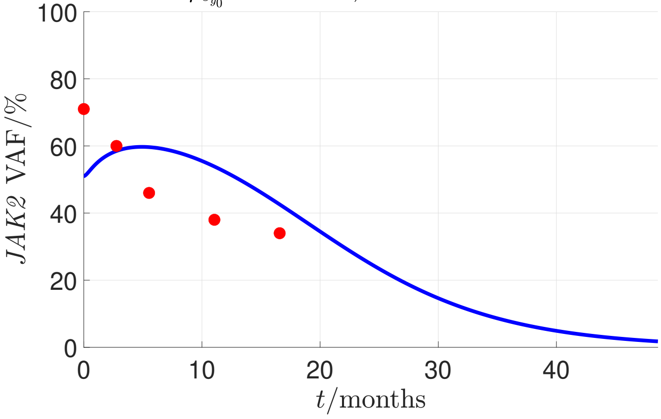


*JAK2* VAF for Patient 9 with RUXO affecting $d_{y_1}$, $\rho_{d_{y_1}}$ = 0.12, RMSE =6.63e-02

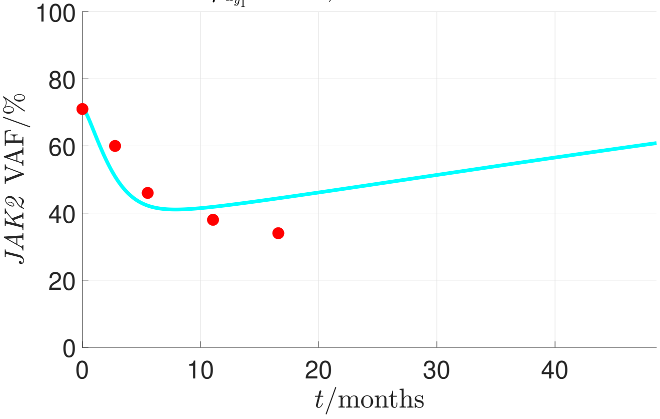


*JAK2* VAF for Patient 10 with RUXO affecting $s_{y_0}$, $\rho_{s_{y_0}}$ = 8270.14, RMSE =1.05e-01

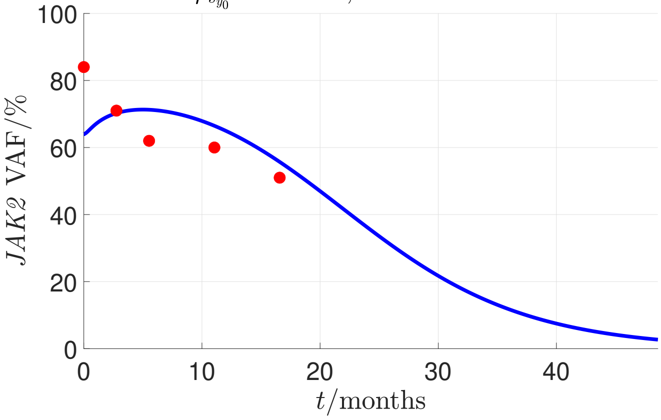


*JAK2* VAF for Patient 10 with RUXO affecting $d_{y_1}$, $\rho_{d_{y_1}}$ = 0.12, RMSE =4.70e-02

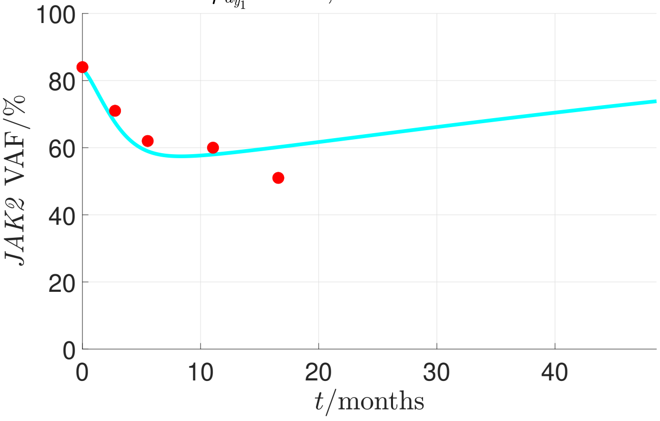


*JAK2* VAF for Patient 11 with RUXO affecting $s_{y_0}$, $\rho_{s_{y_0}}$ = 5584.50, RMSE =1.09e-01

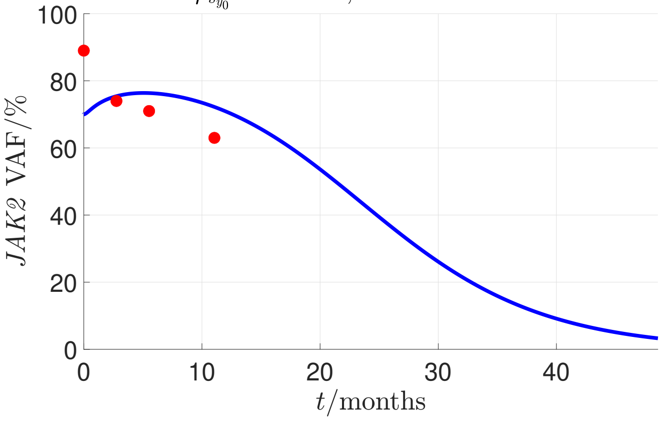


*JAK2* VAF for Patient 11 with RUXO affecting $d_{y_1}$, $\rho_{d_{y_1}}$ = 0.12, RMSE =2.58e-02

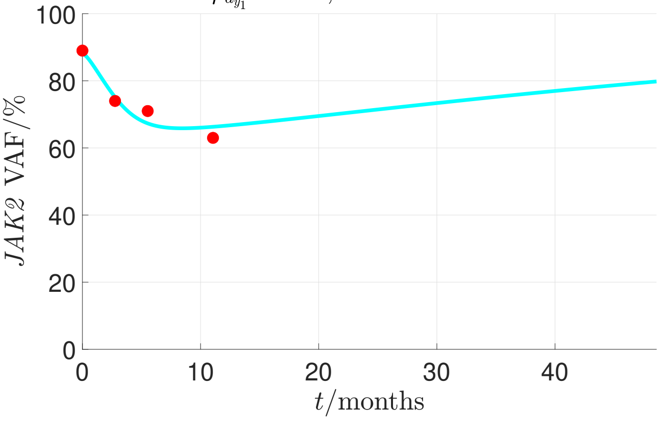

*JAK2* VAF for Patient 12 with RUXO affecting $s_{y_0}$, $\rho_{s_{y_0}} = 29835.31$, RMSE =1.60e-01

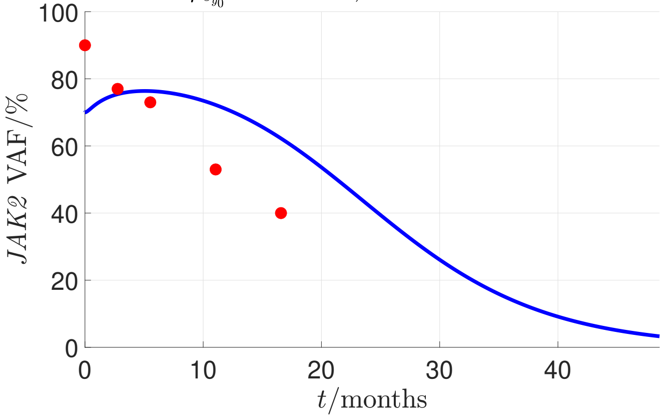


*JAK2* VAF for Patient 12 with RUXO affecting $d_{y_1}$, $\rho_{d_{y_1}} = 516.61$, RMSE =8.54e-02

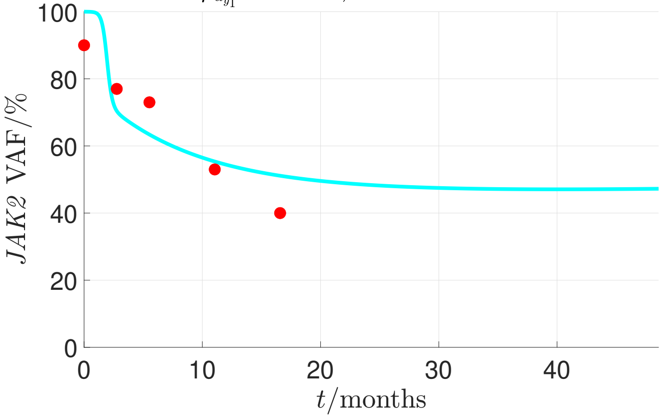


*JAK2* VAF for Patient 13 with RUXO affecting $s_{y_0}$, $\rho_{s_{y_0}} = 7371.66$, RMSE =9.74e-02

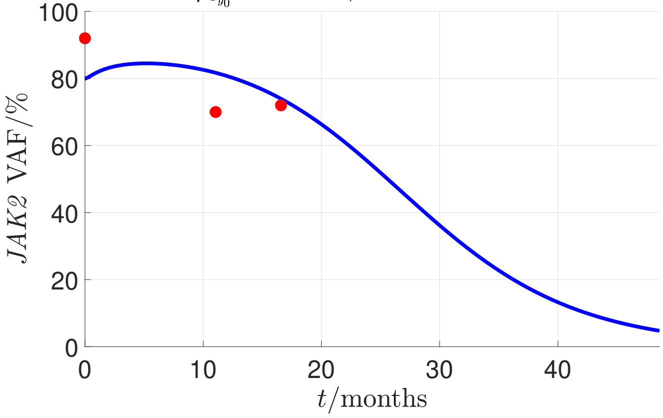


*JAK2* VAF for Patient 13 with RUXO affecting $d_{y_1}$, $\rho_{d_{y_1}} = 0.16$, RMSE =9.38e-04

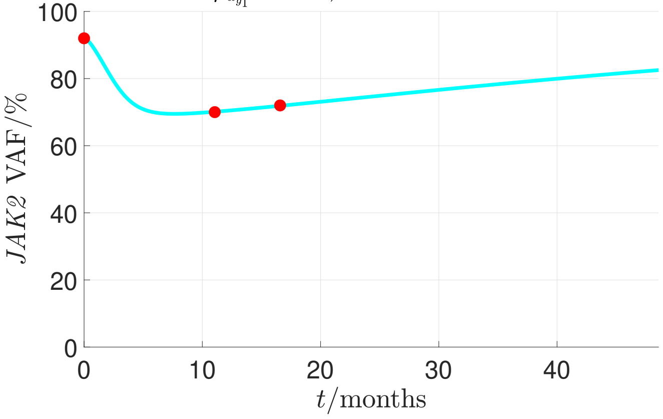


*JAK2* VAF for Patient 14 with RUXO affecting $s_{y_0}$, $\rho_{s_{y_0}} = 8369.53$, RMSE =6.46e-02

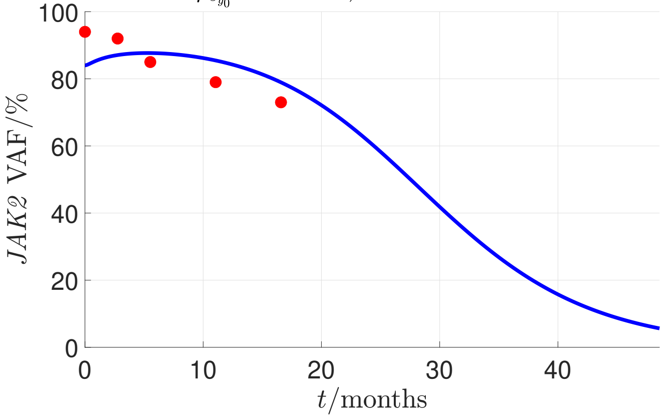


*JAK2* VAF for Patient 14 with RUXO affecting $d_{y_1}$, $\rho_{d_{y_1}} = 176.85$, RMSE =3.94e-02

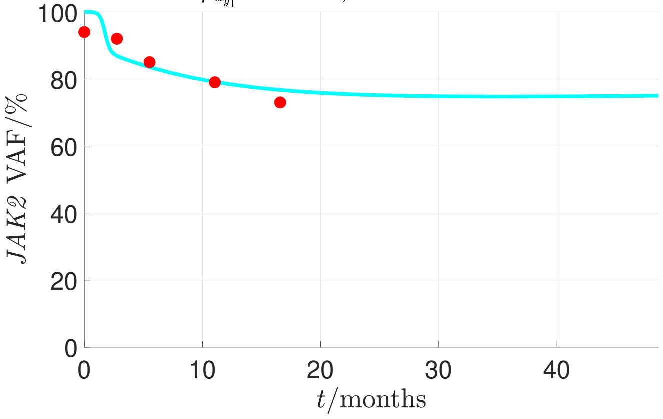

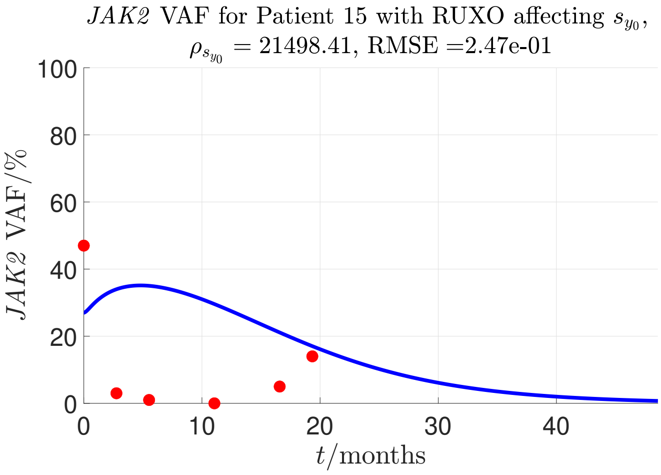
*JAK2* VAF for Patient 15 with RUXO affecting $s_{y0}$,
$\rho_{s_{y0}}$ = 21498.41, RMSE =2.47e-01
*JAK2* VAF/%
$t$/months
0
10
20
30
40
60
80
100

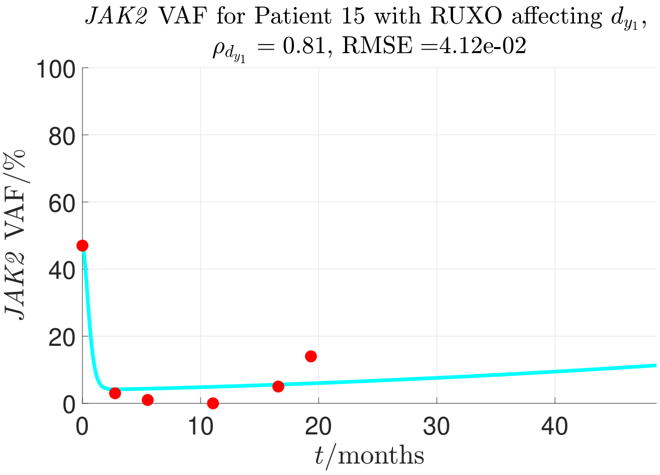
*JAK2* VAF for Patient 15 with RUXO affecting $d_{y1}$,
$\rho_{d_{y1}}$ = 0.81, RMSE =4.12e-02
*JAK2* VAF/%
$t$/months
0
10
20
30
40
60
80
100

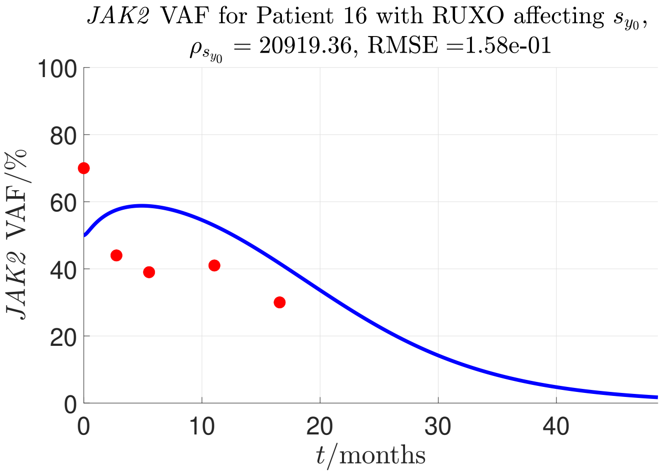
*JAK2* VAF for Patient 16 with RUXO affecting $s_{y0}$,
$\rho_{s_{y0}}$ = 20919.36, RMSE =1.58e-01
*JAK2* VAF/%
$t$/months
0
10
20
30
40
60
80
100

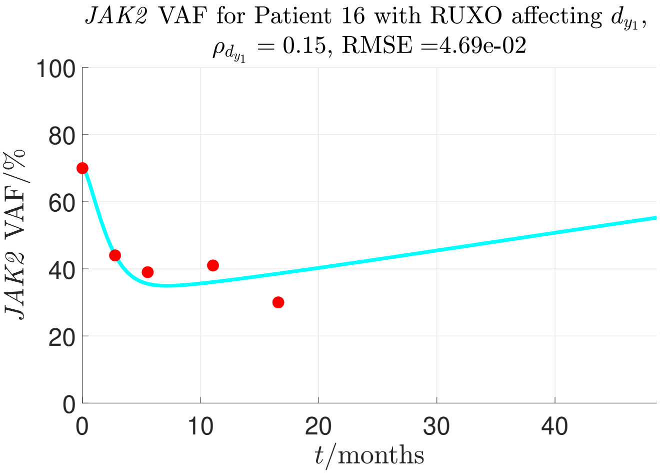
*JAK2* VAF for Patient 16 with RUXO affecting $d_{y1}$,
$\rho_{d_{y1}}$ = 0.15, RMSE =4.69e-02
*JAK2* VAF/%
$t$/months
0
10
20
30
40
60
80
100

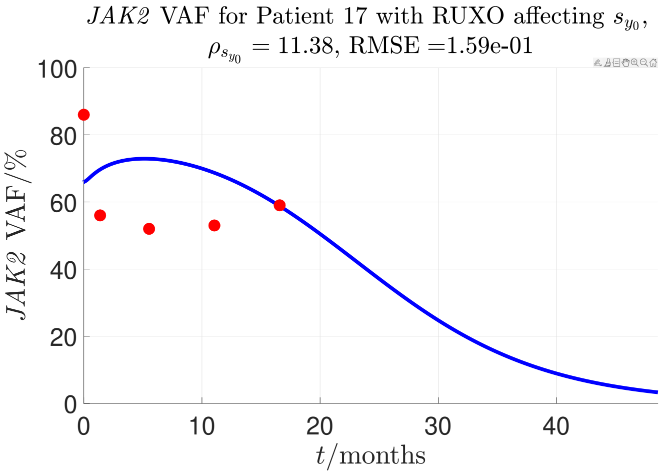
*JAK2* VAF for Patient 17 with RUXO affecting $s_{y0}$,
$\rho_{s_{y0}}$ = 11.38, RMSE =1.59e-01
*JAK2* VAF/%
$t$/months
0
10
20
30
40
60
80
100

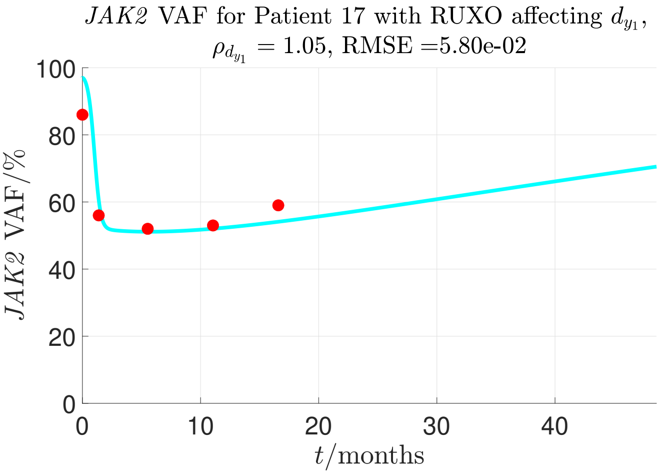
*JAK2* VAF for Patient 17 with RUXO affecting $d_{y1}$,
$\rho_{d_{y1}}$ = 1.05, RMSE =5.80e-02
*JAK2* VAF/%
$t$/months
0
10
20
30
40
60
80
100

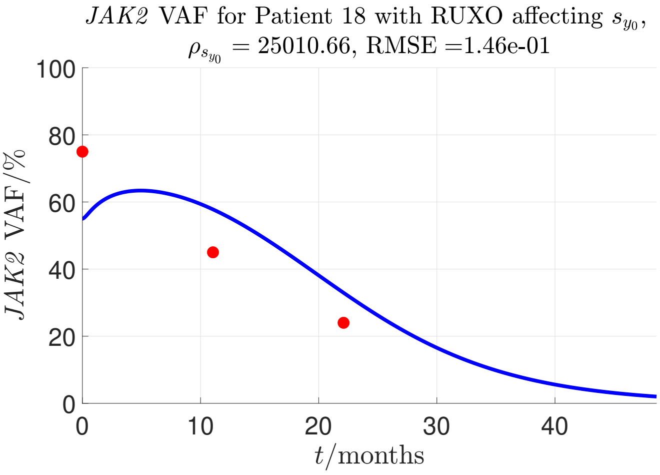
JAK2 VAF for Patient 18 with RUXO affecting $s_{y_0}$,
$\rho_{s_{y_0}}$ = 25010.66, RMSE =1.46e-01
JAK2 VAF/%
t/months
0
10
20
30
40
60
80
100

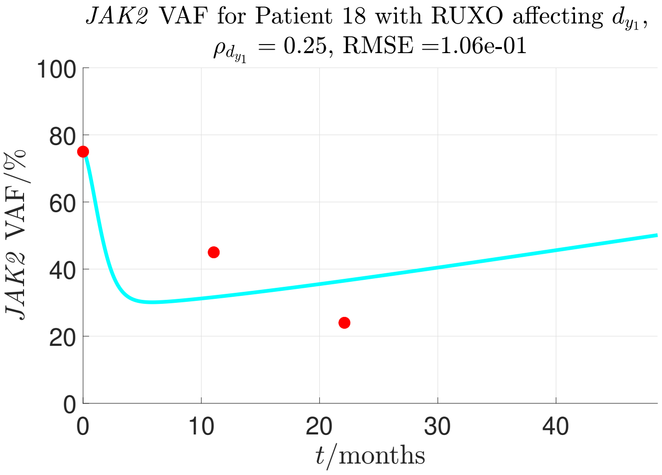
JAK2 VAF for Patient 18 with RUXO affecting $d_{y_1}$,
$\rho_{d_{y_1}}$ = 0.25, RMSE =1.06e-01
JAK2 VAF/%
t/months
0
10
20
30
40
60
80
100

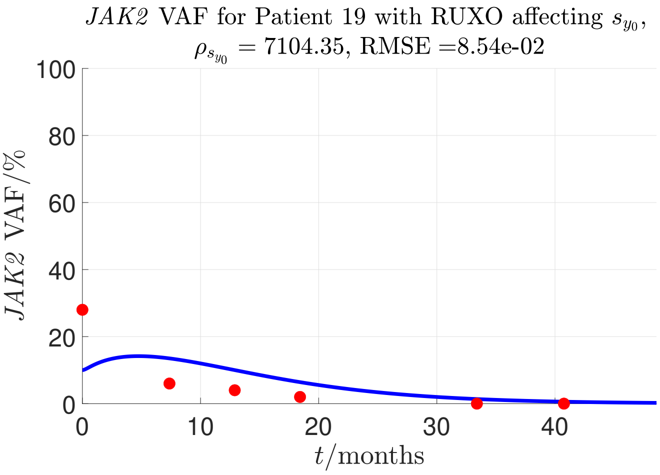
JAK2 VAF for Patient 19 with RUXO affecting $s_{y_0}$,
$\rho_{s_{y_0}}$ = 7104.35, RMSE =8.54e-02
JAK2 VAF/%
t/months
0
10
20
30
40
60
80
100

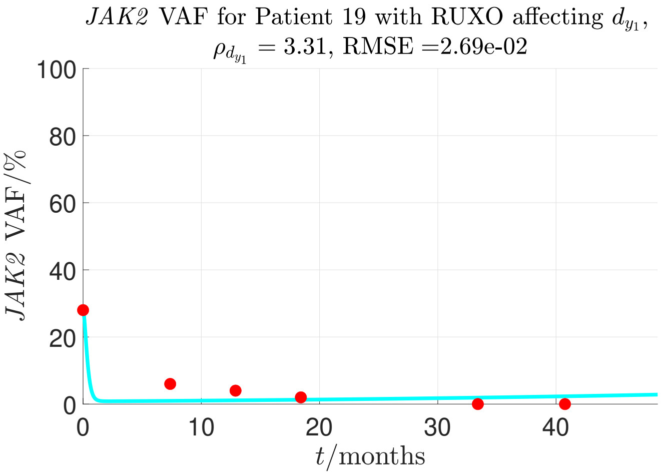
JAK2 VAF for Patient 19 with RUXO affecting $d_{y_1}$,
$\rho_{d_{y_1}}$ = 3.31, RMSE =2.69e-02
JAK2 VAF/%
t/months
0
10
20
30
40
60
80
100

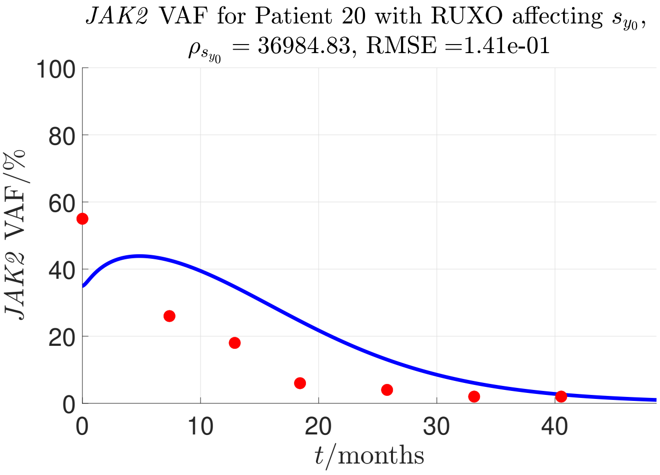
JAK2 VAF for Patient 20 with RUXO affecting $s_{y_0}$,
$\rho_{s_{y_0}}$ = 36984.83, RMSE =1.41e-01
JAK2 VAF/%
t/months
0
10
20
30
40
60
80
100

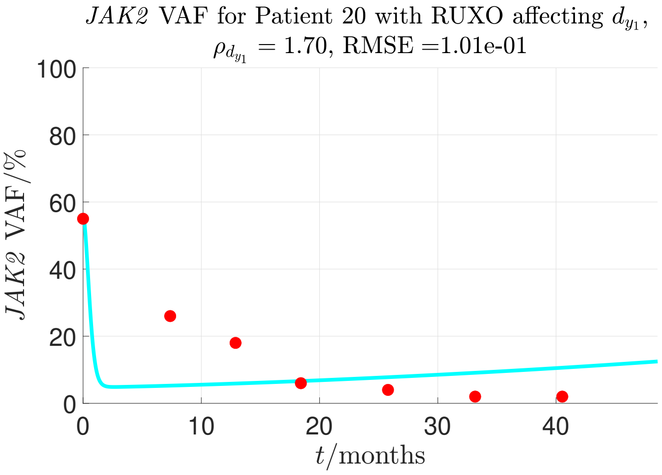
JAK2 VAF for Patient 20 with RUXO affecting $d_{y_1}$,
$\rho_{d_{y_1}}$ = 1.70, RMSE =1.01e-01
JAK2 VAF/%
t/months
0
10
20
30
40
60
80
100

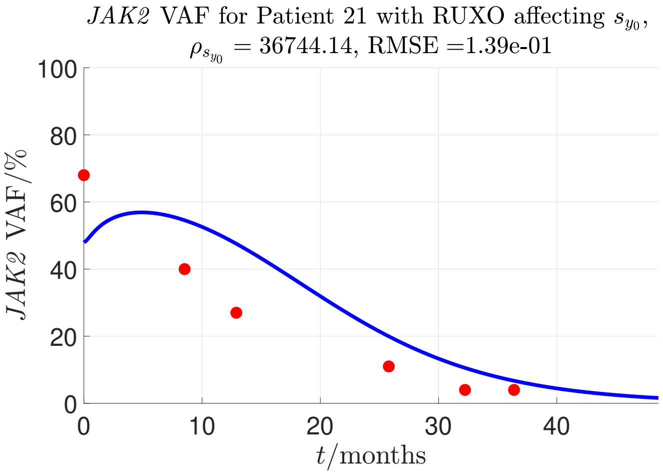
$JAK2$ VAF for Patient 21 with RUXO affecting $s_{y_0}$,
$\rho_{s_{y_0}} = 36744.14$, RMSE =1.39e-01
$JAK2$ VAF/%
$t$/months

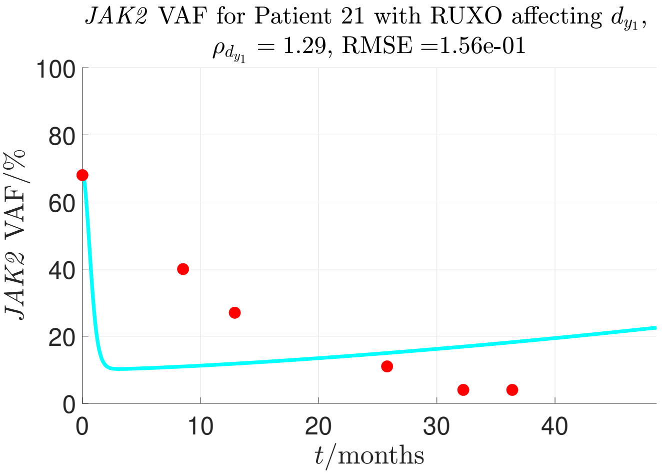
$JAK2$ VAF for Patient 21 with RUXO affecting $d_{y_1}$,
$\rho_{d_{y_1}} = 1.29$, RMSE =1.56e-01
$JAK2$ VAF/%
$t$/months

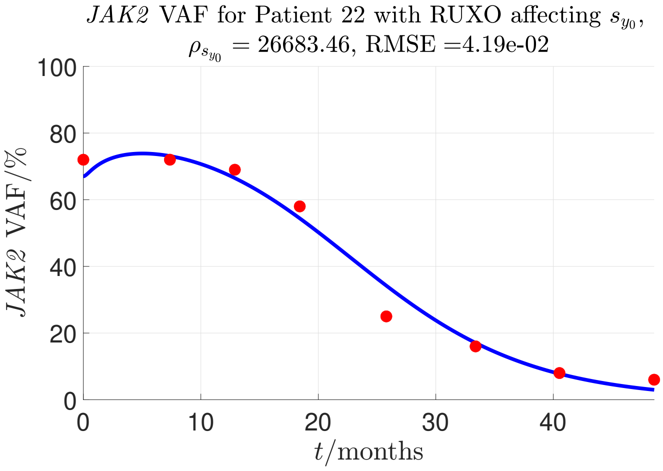
$JAK2$ VAF for Patient 22 with RUXO affecting $s_{y_0}$,
$\rho_{s_{y_0}} = 26683.46$, RMSE =4.19e-02
$JAK2$ VAF/%
$t$/months

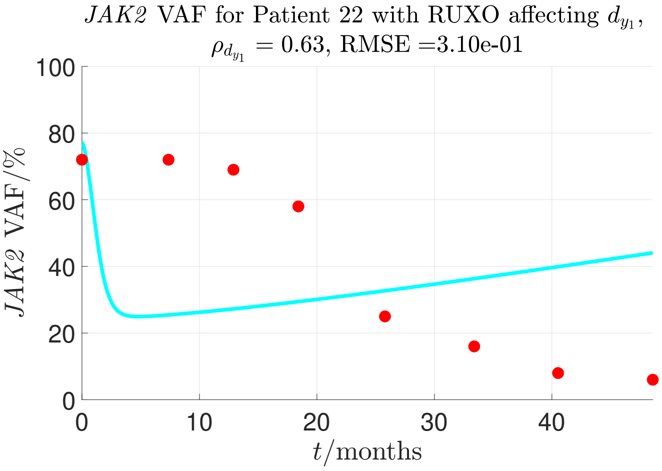
$JAK2$ VAF for Patient 22 with RUXO affecting $d_{y_1}$,
$\rho_{d_{y_1}} = 0.63$, RMSE =3.10e-01
$JAK2$ VAF/%
$t$/months

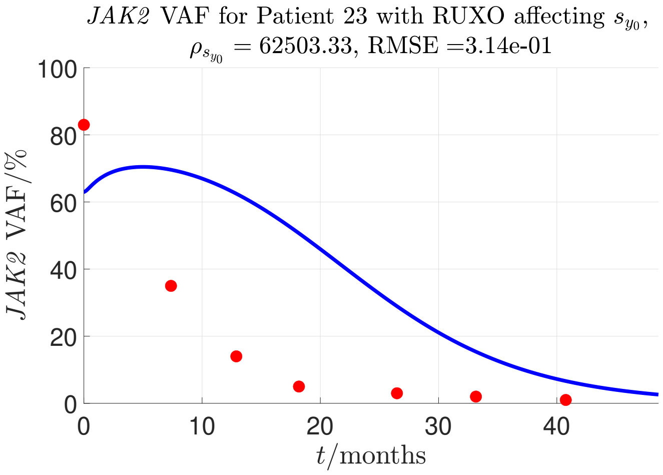
$JAK2$ VAF for Patient 23 with RUXO affecting $s_{y_0}$,
$\rho_{s_{y_0}} = 62503.33$, RMSE =3.14e-01
$JAK2$ VAF/%
$t$/months

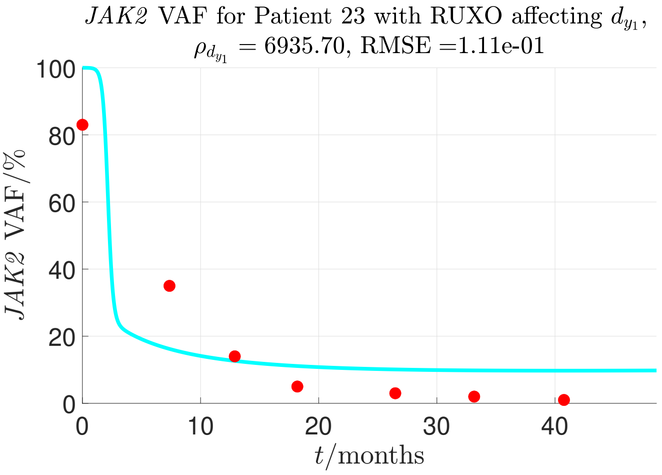
$JAK2$ VAF for Patient 23 with RUXO affecting $d_{y_1}$,
$\rho_{d_{y_1}} = 6935.70$, RMSE =1.11e-01
$JAK2$ VAF/%
$t$/months

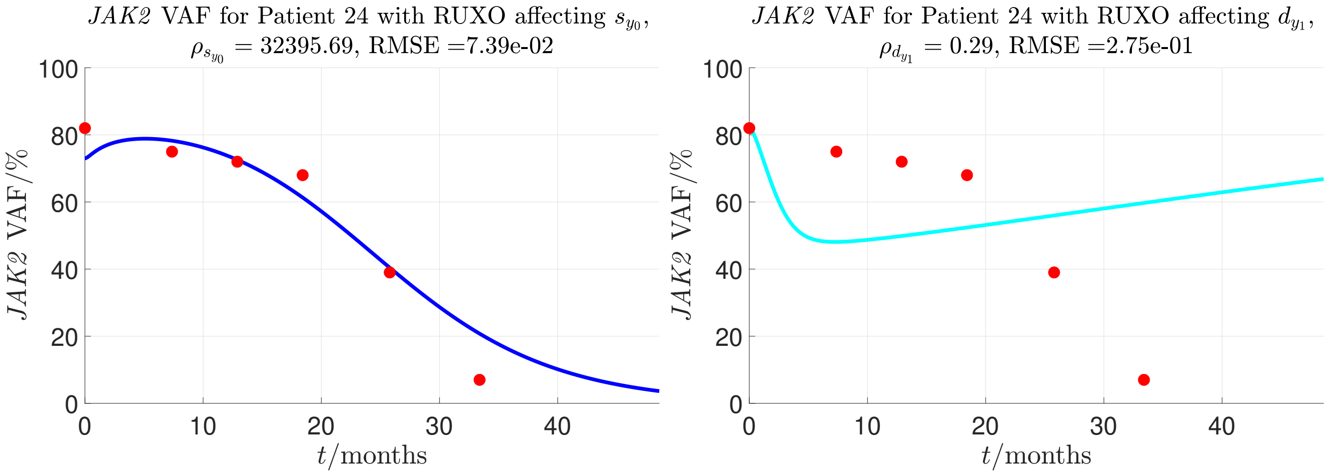

$JAK2$ VAF for Patient 24 with RUXO affecting $s_{y_0}$, $\rho_{s_{y_0}}$ = 32395.69, RMSE =7.39e-02
$JAK2$ VAF/%
$t$/months
0
10
20
30
40
20
60
80
100
$JAK2$ VAF for Patient 24 with RUXO affecting $d_{y_1}$, $\rho_{d_{y_1}}$ = 0.29, RMSE =2.75e-01
$JAK2$ VAF/%
$t$/months

## REFERENCES


**1** .Catlin SN, Busque L, Gale RE, Guttorp P, Abkowitz JL. The replication rate of human hematopoietic stem cells in vivo. *Blood* **117** (2011) 4460–4466. doi:10.1182/blood-2010-08.

**2** .Lee-Six H, Øbro NF, Shepherd MS, Grossmann S, Dawson K, Belmonte M, et al. Population dynamics of normal human blood inferred from somatic mutations. *Nature* **561** (2018) 473–478. doi:10.1038/s41586-018-0497-0.

**3** .Andersen M, Sajid Z, Pedersen RK, Gudmand-Hoeyer J, Ellervik C, Skov V, et al. Mathematical modelling as a proof of concept for MPNs as a human inflammation model for cancer development. *PLOS ONE* **12** (2017) e0183620. doi:10.1371/journal.pone.0183620.

**4** .Ottesen JT, Pedersen RK, Sajid Z, Gudmand-Hoeyer J, Bangsgaard KO, Skov V, et al. Bridging blood cancers and inflammation: The reduced Cancitis model. *Journal of Theoretical Biology* **465** (2019) 90–108. doi:10.1016/j.jtbi.2019.01.001.

**5** .Cosgrove J, Hustin LS, de Boer RJ, Perié L. Hematopoiesis in numbers. *Trends in Immunology* **42** (2021) 1100–1112. doi:10.1016/j.it.2021.10.006.

**6** .Stiehl T, Baran N, Ho AD, Marciniak-Czochra A. Clonal selection and therapy resistance in acute leukaemias: mathematical modelling explains different proliferation patterns at diagnosis and relapse. *Journal of The Royal Society Interface* **11** (2014) 20140079. doi:10.1098/rsif.2014.0079.

**7** .Marciniak-Czochra A, Stiehl T, Ho AD, Jäger W, Wagner W. Modeling of Asymmetric Cell Division in Hematopoietic Stem Cells—Regulation of Self-Renewal Is Essential for Efficient Repopulation. *Stem Cells and Development* **18** (2009) 377–386. doi:10.1089/scd.2008.0143.

**8** .Hasan S, Lacout C, Marty C, Cuingnet M, Solary E, Vainchenker W, et al. JAK2V617F expression in mice amplifies early hematopoietic cells and gives them a competitive advantage that is hampered by IFNa. *Blood* **122** (2013) 1464–1477. doi:10.1182/blood-2013-04-498956.

**9** .Stiehl T, Lutz C, Marciniak-Czochra A. Emergence of heterogeneity in acute leukemias. *Biology Direct* **11** (2016). doi:10.1186/s13062-016-0154-1.

**10** .Pedersen RK, Andersen M, Knudsen TA, Sajid Z, Gudmand-Hoeyer J, Dam MJB, et al. Data-driven analysis of JAK2 V617F kinetics during interferon-alpha2 treatment of patients with polycythemia vera and related neoplasms. *Cancer Medicine* **9** (2020) 2039–2051. doi:10.1002/cam4.2741.

**11** .Fabre MA, de Almeida JG, Fiorillo E, Mitchell E, Damaskou A, Rak J, et al. The longitudinal dynamics and natural history of clonal haematopoiesis. *Nature* **606** (2022) 335–342. doi:10.1038/s41586-022-04785-z.

**12** .Ottesen JT, Pedersen RK, Dam MJB, Knudsen TA, Skov V, Kjær L, et al. Mathematical Modeling of MPNs Offers Understanding and Decision Support for Personalized Treatment. *Cancers* **12** (2020) 2119. doi:10.3390/cancers12082119.

**13** .Hagdrup M, Jørgensen JB. Estimation of parameters in dynamical models. Tech. rep., DTU Compute, Technical University of Denmark (2016).
